# Genome Subtraction and Comparison for the Identification of Novel Drug Targets against *Mycobacterium avium* subsp. *hominissuis*

**DOI:** 10.3390/pathogens9050368

**Published:** 2020-05-12

**Authors:** Reaz Uddin, Bushra Siraj, Muhammad Rashid, Ajmal Khan, Sobia Ahsan Halim, Ahmed Al-Harrasi

**Affiliations:** 1 Dr. Panjwani Center for Molecular Medicine and Drug Research, International Center for Chemical and Biological Sciences, University of Karachi, Karachi 75270, Pakistan; bushrasiraj52@gmail.com (B.S.); m.rashid@iccs.edu (M.R.); 2 Natural and Medical Sciences Research Center, University of Nizwa, P.O. Box 33, Birkat Al Mauz, Nizwa 616, Sultanate of Oman; ajmalkhan@unizwa.edu.om (A.K.); sobia_halim@unizwa.edu.om (S.A.H.)

**Keywords:** *Mycobacterium avium*, tuberculosis, unique metabolic pathways, subtractive genomics, drug target, uncharacterized proteins

## Abstract

*Mycobacterium avium* complex (MAC) is a major cause of non-tuberculous pulmonary and disseminated diseases worldwide, inducing bronchiectasis, and affects HIV and immunocompromised patients. In MAC, *Mycobacterium avium* subsp. *hominissuis* is a pathogen that infects humans and mammals, and that is why it is a focus of this study. It is crucial to find essential drug targets to eradicate the infections caused by these virulent microorganisms. The application of bioinformatics and proteomics has made a significant impact on discovering unique drug targets against the deadly pathogens. One successful bioinformatics methodology is the use of in silico subtractive genomics. In this study, the aim was to identify the unique, non-host and essential protein-based drug targets of *Mycobacterium avium* subsp. *hominissuis* via in silico a subtractive genomics approach. Therefore, an in silico subtractive genomics approach was applied in which complete proteome is subtracted systematically to shortlist potential drug targets. For this, the complete dataset of proteins of *Mycobacterium avium* subsp. *hominissuis* was retrieved. The applied subtractive genomics method, which involves the homology search between the host and the pathogen to subtract the non-druggable proteins, resulted in the identification of a few prioritized potential drug targets against the three strains of *M. avium* subsp. *Hominissuis*, i.e., MAH-TH135, OCU466 and A5. In conclusion, the current study resulted in the prioritization of vital drug targets, which opens future avenues to perform structural as well as biochemical studies on predicted drug targets against *M. avium* subsp. *hominissuis*.

## 1. Introduction

*Mycobacterium* species that do not cause tuberculosis are referred to as non-tuberculous mycobacteria (NTM) and are ubiquitous in nature. NTM cause pulmonary diseases in which organisms of *Mycobacterium avium* complex (MAC) are widely distributed [1]. The incidence rate of infection caused by *M. avium* is found to be higher than that of the other *Mycobacterium* species. For example, a literature survey showed that the pulmonary infection rate in Japan is sevenfold greater by *M. avium* than any other *Mycobacterium* species [2]. MAC consists of two closely linked species, *M. intracellulare* and *M. avium* [3]. Furthermore, *M. avium* is comprised of four subspecies: *M. avium* subsp. *paratuberculosis* (MAP), *M. avium* subsp. *avium* (MAA), *M. avium* subsp. *silvaticum* (MAS) and *M. avium* subsp. *hominissuis* (MAH); and each one is host specific. The first two subspecies cause avian infection, while the third causes diseases in wild livestock and the last one is the most common pathogen in humans and other mammals, including pigs, and therefore has huge economic impact [4].

Opportunistic MAH is responsible for causing disseminated and pulmonary infections that affect immunocompromised patients who are suffering from AIDS, leukemia, lung diseases or chemotherapy [5,6]. The bacterial virulence factor and host-related risk factor contribute to MAC pulmonary diseases. The prevalence of the disease is relatively high in women; however, much of the information about the bacterial virulence factor is still unknown [7]. Environmental risk factors also arise when patients with MAC pulmonary disease are exposed to soil at home or in soil pots [8]. The disease is characterized by adherence to the respiratory mucosa, formation of biofilms [9] and lesions in the linings of epithelial cells of the lungs [7].

MAC pulmonary diseases are controlled by treatment with antibiotics that include macrolide-based multidrug therapy, comprising macrolides (clarithromycin or azithromycin) in combination with rifampin, ethambutol, aminoglycosides (streptomycin or amikacin) and ciprofloxacin [10,11]. However, emerging virulent strains are found to be resistant to these antibiotics [12]. Consequently, these life-threatening microbial pathogens pose an alarming threat for scientists to combat emerging antibiotics resistance. In fact, the emerging strains are capable of becoming more virulent and tolerant to existing drugs [13]. However, the application of genomics has brought about a revolution in the field of drug discovery by providing increased information about the microbial as well as the human genome [14]. This genomic information unveils the mechanism through which pathogens cause the infection. Finding novel and unique drug targets is one of the possible and alternative approaches to overcoming the infections caused by such drug-resistant pathogens. Similarly, finding therapeutic drugs to combat infections of lethal organisms is the most widely applied method albeit with limited success with respect to drug-resistant pathogens [15]. In this scenario, advancements in the fields of computational biology and bioinformatics tools paved the way to propose new and unique drug targets using the subtractive genomics strategy. In the subtractive genomics approach, the genomes of the host and the pathogen are compared, and the non-host pathogen’s unique and essential proteins are proposed as drug targets that are vital to the pathogen’s survival [16,17]. This strategy recognizes genes that are absent in the host, so called “non-host” genes; however, these genes must be present in the pathogen for its survival, replication and sustainability. Additionally, these non-host genes play crucial roles in unique metabolic pathways and mechanisms. Therefore, when the pathogen’s metabolic targets are ideally hit by therapeutic compounds, the therapy must affect the function of the pathogen without altering the host biology [18,19]. The disruption of the essential genes will eventually overcome the pathogen’s infection. Recently, several studies applied the same approach for the identification of potential drug targets of *Acinetobacter baumannii* [20], *Helicobacter pylori* [21], *Mycobacterium* species [22], *Pseudomonas aeruginosa* [23] and others [24,25,26,27,28]. Such computational studies help to minimize experimental efforts with high-speed performance for the prioritization of drug targets. For example, by using the information retrieved from such computational studies, a life scientist can express only the prioritized target gene (which is predicted as a potential drug target), resulting in saving the cost of extra experiments and fostering the research.

## 2. Results and Discussion

With the aim to identify unique and potential druggable targets of *M. avium* subsp. *hominissuis* (MAH), the subtractive genomics method was used, which is the most applicable approach to prioritize potential drug targets [18,29,30,31].

### 2.1. Removal of Duplicate Sequences after Proteome Retrieval

Three strains of MAH, i.e., MAH-TH135, OCU466 and A5, were selected from the available non-redundant strains of *M. avium* subsp. *hominissuis* in the UniProt database. Their complete proteomes were downloaded in FASTA format in February 2019. On applying CD-HIT algorithm with 80% identity, 20 sequences were identified as paralogous out of 4614 proteins in MAH-TH135, 54 out of 5165 in MAH-OCU466 and 14 out of 4502 proteins of A5 strain. The CD-HIT clustered the paralogous sequences and, hence, reduced the total number of sequences of each strain. The sequence dataset was comprised of 4596, 5111 and 4488 protein sequences for the MAH-TH135, OCU466 and A5 strains, respectively.

### 2.2. Searching of Essential, Non-Homologous and Druggable Proteins

In this step, protein sequences that were only present in the pathogens were segregated. Thus, by applying a subtractive approach, sequences were excluded that showed similarity to the human host. The remaining orthologous sequences, retrieved from the previous step, were subjected to BLASTp against the complete human proteome, and the resultant file was parsed. The only sequences that were retained were those that showed “no hits found”, and a total of 3151, 3619 and 3072 non-homologous sequences were found in the MAH-TH135, OCU466 and A5 strains, respectively.

The Database of Essential Genes (DEG) provides information on essential genes of Gram-positive and Gram-negative bacteria determined from experimental methods (http://www.essentialgene.org/). Homology with the sequences found in the DEG database is the basis of essentiality of non-homologous proteins. To do this, the parsed results of each strain from the last step were subjected to BLASTp against the DEG with a 10^−5^ threshold. The BLASTp results depict 1360, 1451 and 1352 essential protein sequences in MAH-TH135, OCU466 and A5, respectively. These identified sequences were considered viable for the pathogen’s life cycle. These sequences include functional, non-functional or uncharacterized proteins, and they were dealt with using different bioinformatics tools for further characterization.

### 2.3. Characterization of Essential Non-Homologous Proteins

#### 2.3.1. Subcellular Localization

**Figure 1:**
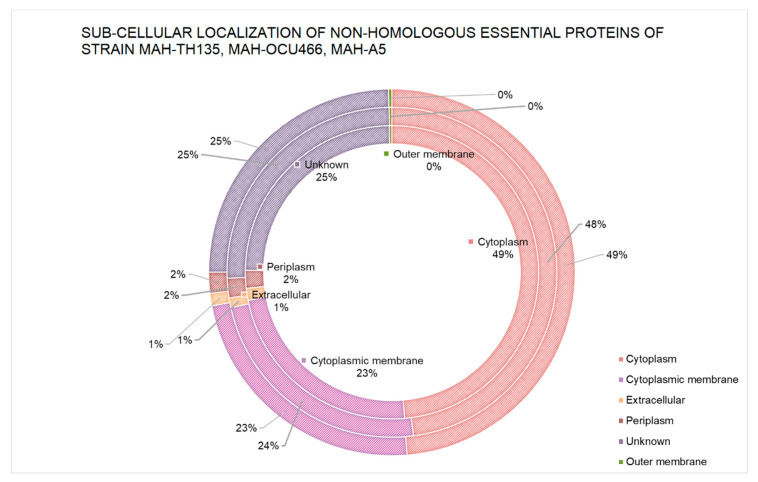
Sub-cellular localization of non-homologous essential proteins. The outermost circle refers to strain MAH-TH135, the middle circle represents strain OCU-466 and the inner circle denotes strain A5.

The tracing of the location of essential proteins is an important facet to understand the functions of proteins in their suitable cell compartments. It is important to know the localization of a drug target in order to optimize the mode of action of the drug for its specific target. The prediction of sub-cellular localization of the essential non-homologous protein sequences was achieved by a computational tool called PSORTb. The results depict that approximately 48% of proteins resided in the cytoplasm of each strain. A proportion of 23% was distributed in the cytoplasmic membrane. The rest of the proteins were present in different regions, including ~1% of proteins in the extracellular region, > 1.5% proteins in the periplasm and very few proteins in the outer membrane of each of the strains. Despite these results, some fractions were considered “unknown” due to the tool’s prediction of proteins in multiple sites simultaneously. The distribution of proteins by PSORTb is graphically shown for each strain in Figure 1.

#### 2.3.2. Functional Family Classification

**Figure 2:**
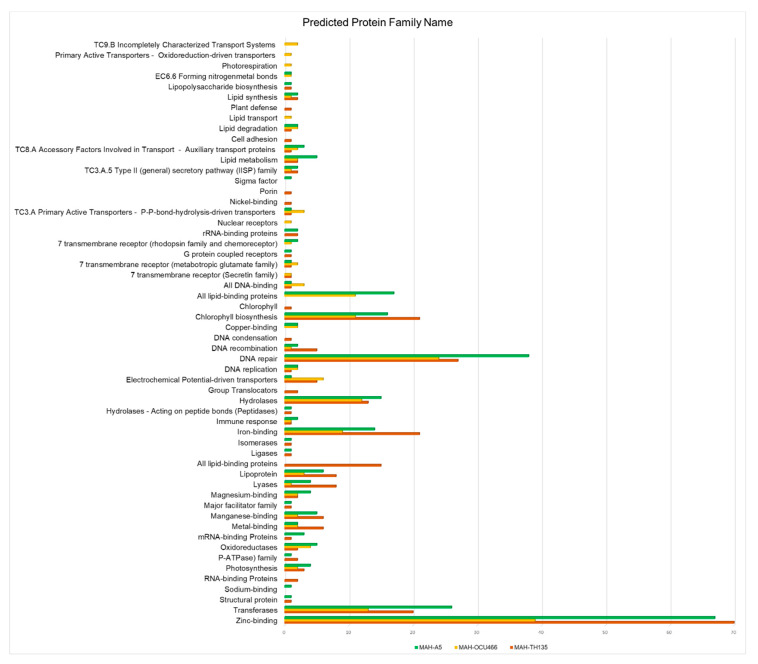
Functional family prediction of *M. avium* subsp. *Hominissuis* (MAH) strains by the SVM-Prot method. The x-axis reports the frequency of each protein family.

The functional families of protein sequences were also determined using the Support Vector Machine of Proteins (SVM-Prot) tool. Only the sequences whose functions were not known earlier were submitted to this tool. Hence, only uncharacterized sequences were retrieved from the non-homologous essential proteins’ sequences. About 193, 119 and 187 uncharacterized sequences of TH135, OCU466 and A5 strains, respectively, were predicted by the SVM-Prot method. The results of the SVM-Prot tool are depicted in Figure 2. The proteins were broadly classified based on their molecular and biological functions and were further sub-divided into several protein classes, i.e., enzymes, transporters, trans-membranes, zinc or magnesium binding or other elements, DNA condensation, repair, etc. Complete information on classes with their strains is summarized in Supplementary Table S1.

#### 2.3.3. Metabolic Pathway Analysis via KEGG

**Figure 3:**
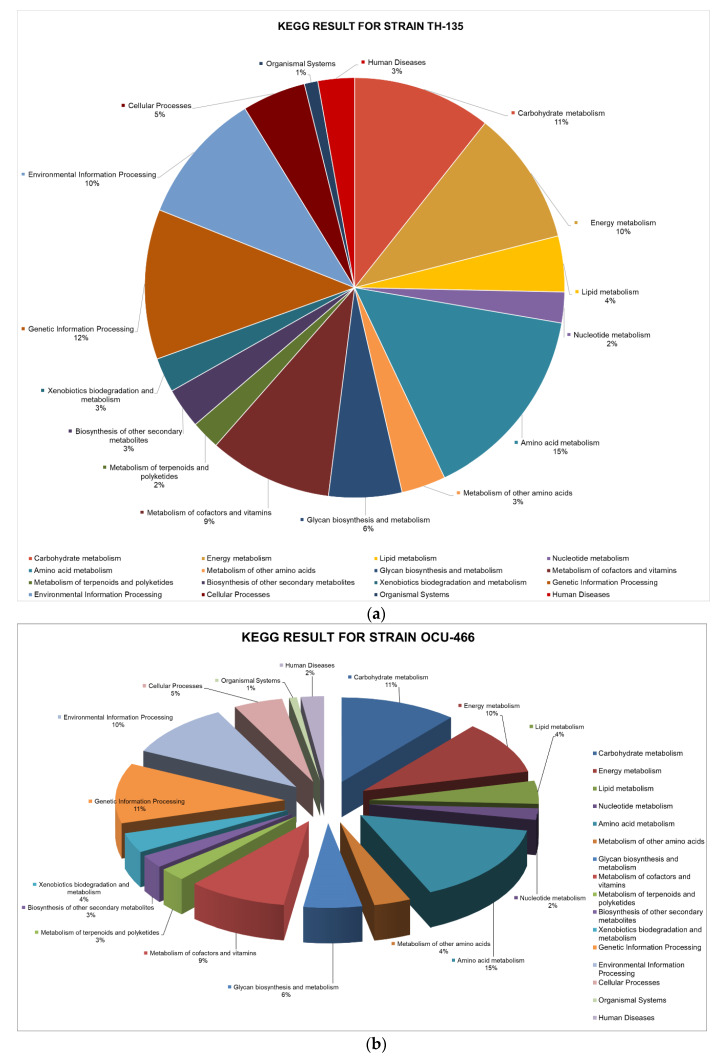

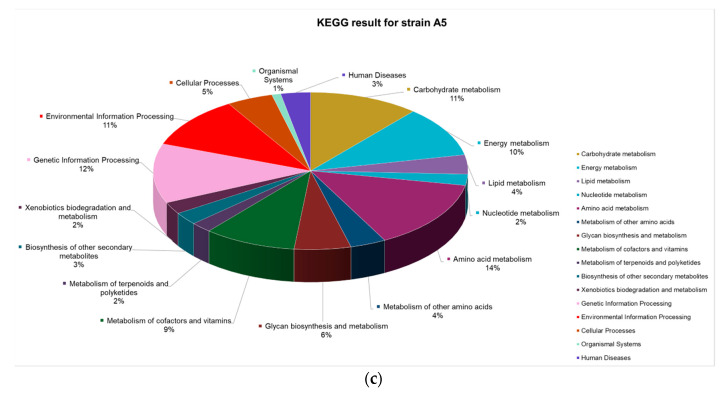
Percent distribution of non-homologous essential proteins involved in different metabolic pathways of the (**a**) MAH-TH135, (**b**) MAH-OCU466 and (**c**) MAH-A5 strains.

The KEGG database provides a network of metabolic pathways with their complete annotation. It helps to predict which protein sequences are essential in playing a unique role in metabolism. This step predicts the potential drug target based on the pathogen’s unique metabolism. Metabolic pathways analysis was carried out for the essential protein sequences using the KEGG database. The DEG’s results were subjected to the KEGG database via the KEGG Automated Annotation Server (KAAS). Briefly, out of 675 protein sequences of the MAH-135 strain, 72, 70, 29, 16 and 103 proteins were found to take part in carbohydrate metabolism, energy metabolism, lipid metabolism, nucleotide metabolism and amino acid metabolism, respectively. For OCU-466, 76 were involved in carbohydrate metabolism, while 69, 30 and 15 took part in energy metabolism, lipid metabolism and nucleotide metabolism, respectively, whereas the A5 strain possessed 93 proteins that majorly contributed to amino acid metabolism. The distribution of proteins in different metabolisms is presented in Figure 3a–c. Details are provided in Supplementary Tables S2–S4.

### 2.4. Discussion of Significant Unique Metabolic Pathways (UMPs) of the Pathogens

Bacterial metabolism refers to the collection of the biochemical reactions required for bacterial survival and growth, which mainly includes respiration (aerobic and anaerobic) and fermentation. Bacteria, as a pathogen to humans, conduct all the same types of basic biochemical reactions a human cell performs. However, bacteria may have several types of energy generating metabolisms that do not exist in human or eukaryotic cells. This diversity of energy generation and metabolism allows bacteria to survive in a variety of habitats and flourish in otherwise not-suitable conditions. On the other hand, these differential metabolic pathways make bacteria susceptible by serving as an ideal target for antibiotics. Metabolic pathways that exist only in pathogens are called unique metabolic pathways (UMP). These UMPs are listed in Supplementary Table S5. We provide brief information on some bacterial UMPs and their significance as an antibiotic target. 

#### 2.4.1. Energy Metabolism

Energy is a potential, needed to perform work and maintain life, usually acquired by breaking a chemical bond and stored by making another chemical bond, very often in the form of ATP. Methane metabolism is one of the UMPs by which bacteria can obtain energy by oxidizing one-carbon compounds (e.g., methanol, methane). Methanotrophic bacteria are generally considered environmentally friendly organisms, as they contribute to oxidizing environmental methane, thereby mitigating the effects of global warming [32]. Methane monooxygenases are the main enzymes to catalyze methane oxidation [33]. There are several UMPs in bacteria, which are related to photosynthesis and carbon fixation and can be exploited for the purpose of drug target identification. 

#### 2.4.2. Biosynthesis of Secondary Metabolites

Secondary metabolites are molecules not essentially required for the survival of an organism. A large portion of bacterial metabolism deals with the biosynthesis of secondary metabolites. However, these pathways have a minimal role in bacterial growth and viability and are not considered a suitable target for antibiotics. Even though secondary metabolites are not considered to be ideal as drug targets, many of these pathways are manipulated by researchers for valuable purposes such as penicillin and cephalosporin biosynthesis, carbapenem biosynthesis and streptomycin biosynthesis. 

#### 2.4.3. Amino Acid Metabolism

Amino acid metabolism in bacteria is diverse in nature and performs a pivotal role in maintaining bacterial growth. Amino acid metabolism has emerged as a potential target for new antibiotics, and a number of new drug targets have been proposed in recent years [34,35,36,37]. Some of these drug targets have shown promising results. Lysine biosynthesis, an essential pathway in bacteria for survival and growth, is reported to be a potential target for antibiotics [38,39]. Similarly, *D*-alanine metabolism is a significant target; an antibiotic *D*-cycloserine targeting *D*-alanine metabolism is already in clinical use against *Mycobacterium tuberculosis* [40,41]. The heterogeneity of amino acid metabolism implies an enormous scope for discovering new antibiotic targets using modern computational tools. 

Other types of metabolic activities in bacteria, such as terpenoids and polyketides, glycan biosynthesis and drug resistance, also perform supportive functions for bacterial growth and survival; however, these metabolic routes are not prioritized targets for anti-bacterial drugs. Rather, these metabolic routes are often manipulated for advantageous purposes [42]. 

### 2.5. Shortlisting of Proteins Sequences as Druggable

The potential drug targets were shortlisted based on obtained information from earlier successful literature reports. The druggability of non-host uncharacterized protein sequences was determined by performing BLASTp against the druggable protein sequences present in the DrugBank Database. For this purpose, the earlier shortlisted, non-host, uncharacterized proteins, which are essential in metabolic pathways, were analyzed for druggability by comparing their sequences with the DrugBank Database. In this search, only one protein was prioritized in TAH-135, whereas four and seven potential drug targets emerged with the OCU-466 and A5 strains, respectively (Table 1). All these potential drug targets were similar to the FDA-approved drug target sequences in the DrugBank Database, including the DNA polymerase III subunit ε of the TH-135 strain, Inter-α-trypsin inhibitor heavy chain H4, exopolyphosphatase, DNA polymerase III subunit ε, mannoside ABC transport system and sugar-binding protein of the OCU-466 strain. In addition to all the proteins from the OCU-466 strain, diacylglycerol acyltransferase/mycolyltransferase, Ag85C and nickel-binding periplasmic protein were found for the A5 strain.

It is noteworthy that all the proposed drug targets could be analyzed for 3D structural information to prioritize novel drug targets against pathogens. Therefore, BLASTp was performed for the target proteins against the Protein Data Bank (PDB) database, which revealed that 12 protein sequences had no 3D structure available yet in the PDB. Therefore, this study offers those 12 proteins’ sequences to not only consider as a potential druggable genome, but also for future studies of 3D structure determination either by homology modeling (template-based) or by ab initio (template-free) methods [43].

## 3. Materials and Methods

**Figure 4:**
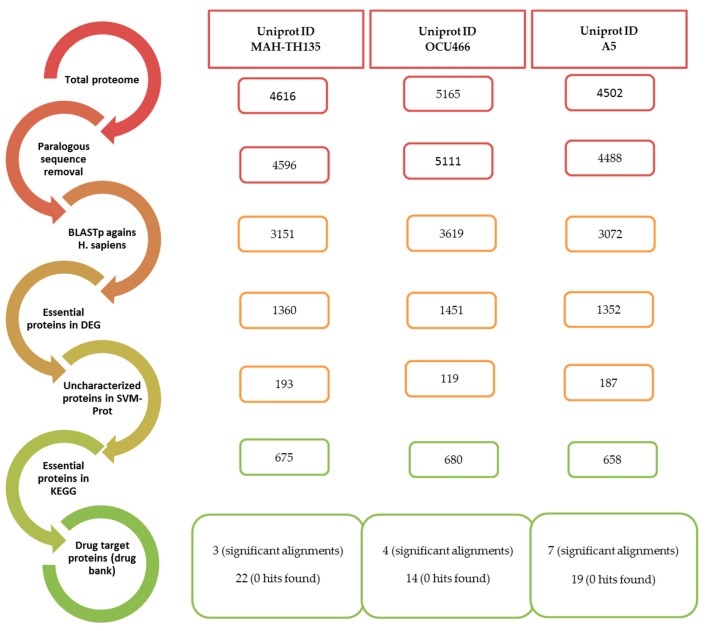
Workflow of the subtractive genomics approach.

An overview of the subtractive genomics approach is illustrated in Figure 4.

### 3.1. Extraction of the Host–Pathogen Proteome

The whole proteome of the host, i.e., *Homo sapiens*, and pathogen, i.e., *Mycobacterium avium* subsp. *Hominissuis,* were downloaded from the UniProt KB database [44] to retrieve protein sequences. The drug target identification approach was carried out on the pathogenic MAH-TH135, MAH-OCU466 and A5 strains. 

### 3.2. Grouping of Common Proteins in All Strains

The CD-HIT tool [45] clusters the protein or nucleotide sequences and reduces redundancy and manual efforts in sequence analysis. This tool was used as a standalone command line tool to remove paralogous or duplicated sequences of all strains with a threshold value of 80%. The remaining set of proteins was grouped as orthologous sequences. 

### 3.3. Identification of Non-Homologous Proteins

Standalone BLAST version 2.8.1 was downloaded from the NCBI FTP server [46]. The orthologous sequences were subjected to BLASTp against the *H. sapiens* database with an expectation value (e-value) of 10^−3^ [47]. The output was obtained with keywords of “no hits found” for unique proteins and “significant alignments” for the sequences having similarity with the human (host) proteome. The results were analyzed, and only protein sequences “with no homology with the human host” were retained, while the rest were removed. Those proteins were further labelled as non-homologous proteins, and finally, they were extracted using our in-house scripts.

### 3.4. Finding of Essential Genes

The genes required to sustain the life cycle of bacteria are called essential genes. The Database of Essential Genes (DEG) contains lists of genes with their corresponding sequences, which are essential for the survival of bacterial life. [48]. Therefore, the DEG was used to find the sequences that are essential to the bacterial pathogen studied here (i.e., *M. avium* subsp. *hominissuis*). The non-homologous proteins were aligned with the DEG database using BLASTp, and the expectation value was set to 10^−5^. As a result, the non-homologous essential genes, which may have hypothetical or uncharacterized proteins, were obtained.

### 3.5. Information about Metabolic Pathways

The metabolic pathways of the identified non-homologous essential proteins were searched in the Kyoto Encyclopedia of Genes and Genomes (KEGG) [49] through the KAAS server. KAAS [50] uses BLASTp for the comparison of query proteins against the KEGG database and annotates functions. KAAS provides the KEGG Orthology (KO) identifiers and information on the metabolic pathways of the proteins. 

### 3.6. Annotation of the Curated Proteins

Annotation of proteins includes information about the location of proteins in various regions of the cell and the family to which it belongs. PSORTb version 3.0 [51] is well known to predict the subcellular localization (SCL) of proteins. The SCL includes different compartments, such as cytoplasmic membrane, cytoplasm, cell wall and extracellular and unknown regions of the cell where the proteins reside. All the non-homologous essential, as well as hypothetical, proteins were subjected to the protein databases with known functions using SCL BLAST by the web-based server. SVM-Prot [52] is an online tool for the classification of protein functional families. It applies the machine-learning method and predicts a diverse set of molecular and biological functions covering all major classes of enzymes, channels, transporters, receptors, DNA/RNA binding proteins, etc. and covering 192 functional families of proteins. Those proteins whose functions are still unknown were labeled as non-homologous, hypothetical/uncharacterized proteins and passed through the server of SVM-Prot to classify them into functional families.

### 3.7. Druggability of the Shortlisted Sequences

In order to detemine the novel drug targets, standalone BLASTp was run between hypothetical non-homologous essential proteins, and drug target sequences were taken from the DrugBank Database [53] with an e-value cutoff 10^−3^. The DrugBank Database provides detailed information on drugs and drug targets. A large database shows up to 8261 drugs, including FDA-approved drugs; experimental and nutraceutical drugs are available in the DrugBank Database.

## 4. Conclusions

Different bioinformatics tools were applied in this study to identify vital drug targets of *Mycobacterium avium* subsp. *hominissuis*. Protein sequences of *M. avium* subsp. *hominissuis* were parsed using multiple steps of the subtractive genomics approach, and a few of them were shortlisted as possible drug targets because they fulfilled the druggability criteria. The shortlisted sequences were non-homologous to the human host; thus, these can be proposed as ideal drug targets. All the identified drug targets of different strains of MAH have never been characterized before as drug targets, and we proposed them here as potential drug targets against which new drug compounds can be designed. Therefore, the study is significant to the scientific community, as it provides a prioritized list of possible drug targets sorted by the computational subtractive genomics method, and it has the potential to lead to the discovery of new and novel drug targets against *M. avium* subsp. *hominissuis*. 

## Figures and Tables

**Table 1:** Protein drug targets of *M. avium* subsp. *hominissuis.*

**UNIPROT STRAIN ID MAH-TH135**				
**S. No.**	**Protein ID**	**DrugBank target name**	**DrugBank ID**	**Localization Site**
1.	T2GUW6	DNA polymerase III subunit epsilon (DB01643)	P03007	Cytoplasmic
**UNIPROT STRAIN ID MAH-OCU466**				
**S. No.**	**Protein ID**	**DrugBank target name**	**DrugBank ID**	**Localization Site**
1.	A0A2A3L1J8	Inter-alpha-trypsin inhibitor heavy chain H4 (DB01593; DB14487; DB14533) Inter-alpha-trypsin inhibitor heavy chain H4 (DB01593; DB14487; DB14533)	Q14624 Q06033	Cytoplasmic
2.	A0A2A3L805	O67040 Exopolyphosphatase (DB03382)	O67040	Cytoplasmic
3.	A0A2A3L3Y2	DNA polymerase III subunit epsilon (DB01643)	P03007	Cytoplasmic
4.	A0A2A3LDY9	Mannoside ABC transport system, sugar-binding protein (DB01942)	Q9X0V0	Unknown
**UNIPROT STRAIN ID MAH-A5**				
**S. No.**	**Protein ID**	**DrugBank target name**	**DrugBank ID**	**Localization Site**
1.	A0A0E2W125	Exopolyphosphatase (DB03382)	O67040	Cytoplasmic
2.	A0A0E2W9K2	Inter-alpha-trypsin inhibitor heavy chain H4 (DB01593; DB14487; DB14533) Inter-alpha-trypsin inhibitor heavy chain H4 (DB01593; DB14487; DB14533)	Q14624 Q06033	Cytoplasmic
3.	A0A0E2W6U1	Diacylglycerol acyltransferase/mycolyltransferase Ag85C (DB02811; DB08558)	P9WQN9	Unknown (This protein may have multiple localization sites.)
4.	A0A0E2W8I5	Diacylglycerol acyltransferase/mycolyltransferase Ag85C (DB02811; DB08558)	P9WQN9	Extracellular
5.	A0A0E2W8U0	DNA polymerase III subunit epsilon (DB01643)	P03007	Cytoplasmic
6.	A0A0E2WAR7	Mannoside ABC transport system, sugar-binding protein (DB01942) Nickel-binding periplasmic protein (DB03374)	Q9X0V0 P33590	Unknown
7.	A0A0E2WQA2	Mannoside ABC transport system, sugar-binding protein (DB01942) Nickel-binding periplasmic protein (DB03374) Periplasmic oligopeptide-binding protein (DB07365) ABC transporter, periplasmic substrate-binding protein (DB02078)	Q9X0V0 P33590 P06202 Q5LRQ9	Periplasmic

## Supplementary Materials

The following are available online at https://www.mdpi.com/2076-0817/9/5/368/s1, Table S1; Table S2; Table S3; Table S4; Table S5.

## References

[1] Daley C. (2017). Mycobacterium avium Complex Disease. Microbiol. Spectr..

[2] Iwamoto T., Nakajima C., Nishiuchi Y., Kato T., Yoshida S., Nakanishi N., Tamaru A., Tamura Y., Suzuki Y., Nasu M. (2012). Genetic diversity of *Mycobacterium avium* subsp. *hominissuis* strains isolated from humans, pigs, and human living environment. Infect. Genet. Evol..

[3] Uchiya K.-I., Takahashi H., Yagi T., Moriyama M., Inagaki T., Ichikawa K., Nakagawa T., Nikai T., Ogawa K. (2013). Comparative genome analysis of *Mycobacterium avium* revealed genetic diversity in strains that cause pulmonary and disseminated disease. PLoS ONE.

[4] Mijs W., De Haas P., Rossau R., Van Der Laan T., Rigouts L., Portaels F., Van Soolingen D. (2002). Molecular evidence to support a proposal to reserve the designation *Mycobacterium avium* subsp. avium for bird-type isolates and ‘*M. avium* subsp. *hominissuis*’ for the human/porcine type of *M. avium*. Int. J. Syst. Evol. Micr..

[5] Porvaznik I., Solovič I., Mokrý J. (2016). Non-tuberculous mycobacteria: Classification, diagnostics, and therapy. Respiratory Treatment and Prevention.

[6] Bruffaerts N., Vluggen C., Roupie V., Duytschaever L., Van den Poel C., Denoël J., Wattiez R., Letesson J.-J., Fretin D., Rigouts L. (2017). Virulence and immunogenicity of genetically defined human and porcine isolates of *M. avium* subsp. *hominissuis* in an experimental mouse infection. PLoS ONE.

[7] Uchiya K.-I., Takahashi H., Nakagawa T., Yagi T., Moriyama M., Inagaki T., Ichikawa K., Nikai T., Ogawa K. (2015). Characterization of a novel plasmid, pMAH135, from *Mycobacterium avium* subsp. *hominissuis*. PLoS ONE.

[8] Maekawa K., Ito Y., Hirai T., Kubo T., Imai S., Tatsumi S., Fujita K., Takakura S., Niimi A., Iinuma Y. (2011). Environmental risk factors for pulmonary *Mycobacterium avium-*intracellulare complex disease. Chest.

[9] Weiss C., Glassroth J. (2012). Pulmonary disease caused by nontuberculous mycobacteria. Expert. Rev. Respir. Med..

[10] Uchiya K.-I., Asahi S., Futamura K., Hamaura H., Nakagawa T., Nikai T., Ogawa K. (2018). Antibiotic susceptibility and genotyping of *Mycobacterium avium* strains that cause pulmonary and disseminated infection. Antimicrob. Agents Chemother..

[11] Blanchard J.D., Elias V., Cipolla D., Gonda I., Bermudez L.E. (2018). Effective Treatment of *Mycobacterium avium* subsp. hominissuis and *Mycobacterium abscessus* Species Infections in Macrophages, Biofilm, and Mice by Using Liposomal Ciprofloxacin. Antimicrob. Agents Chemother..

[12] Griffith D.E., Brown-Elliott B.A., Langsjoen B., Zhang Y., Pan X., Girard W., Nelson K., Caccitolo J., Alvarez J., Shepherd S. (2006). Clinical and molecular analysis of macrolide resistance in *Mycobacterium avium* complex lung disease. Am. J. Respir. Crit. Care Med..

[13] Nicolle L. (2006). Community-acquired MRSA: A practitioner's guide. CMAJ.

[14] Rathi B., Sarangi A.N., Trivedi N. (2009). Genome subtraction for novel target definition in *Salmonella typhi*. Bioinformation.

[15] Butt A.M., Tahir S., Nasrullah I., Idrees M., Lu J., Tong Y. (2012). Mycoplasma genitalium: A comparative genomics study of metabolic pathways for the identification of drug and vaccine targets. Infect. Genet. Evol..

[16] Barh D., Tiwari S., Jain N., Ali A., Santos A.R., Misra A.N., Azevedo V., Kumar A. (2011). *In silico* subtractive genomics for target identification in human bacterial pathogens. Drug Develop. Res..

[17] Bottacini F., Motherway M.O.C., Kuczynski J., O’Connell K.J., Serafini F., Duranti S., Milani C., Turroni F., Lugli G.A., Zomer A. (2014). Comparative genomics of the Bifidobacterium breve taxon. BMC Genom..

[18] Uddin R., Saeed K. (2014). Identification and characterization of potential drug targets by subtractive genome analyses of methicillin resistant *Staphylococcus aureus*. Comput. Biol. Chem..

[19] Galperin M.Y., Koonin E.V. (1999). Searching for drug targets in microbial genomes. Curr. Opin. Biotechnol..

[20] Uddin R., Masood F., Azam S.S., Wadood A. (2019). Identification of putative non-host essential genes and novel drug targets against *Acinetobacter baumannii* by *in silico* comparative genome analysis. Microb. Pathog..

[21] Dutta A., Singh S.K., Ghosh P., Mukherjee R., Mitter S., Bandyopadhyay D. (2006). *In silico* identification of potential therapeutic targets in the human pathogen *Helicobacter pylori*. Silico Biol..

[22] Marri P.R., Bannantine J.P., Golding G.B. (2006). Comparative genomics of metabolic pathways in Mycobacterium species: Gene duplication, gene decay and lateral gene transfer. FEMS Microbiol. Rev..

[23] Uddin R., Jamil F. (2018). Prioritization of potential drug targets against *P. aeruginosa* by core proteomic analysis using computational subtractive genomics and protein-Protein interaction network. Comput. Biol. Chem..

[24] Ahmad S., Navid A., Akhtar A.S., Azam S.S., Wadood A., Pérez-Sánchez H. (2019). Subtractive Genomics, Molecular Docking and Molecular Dynamics Simulation Revealed LpxC as a Potential Drug Target Against Multi-Drug Resistant *Klebsiella pneumoniae*. Interdiscipl. Sci. Comput. Life Sci..

[25] Asalone K.C., Nelson M.M., Bracht J.R. (2019). Novel Sequence Discovery by Subtractive Genomics. J. Vis. Exp..

[26] Nayak S., Pradhan D., Singh H., Reddy M.S. (2019). Computational screening of potential drug targets for pathogens causing bacterial pneumonia. Microb. Pathog..

[27] Prabha R., Singh D.P., Ahmad K., Kumar S.P.J., Kumar P. (2019). Subtractive genomics approach for identification of putative antimicrobial targets in *Xanthomonas oryzae* pv. oryzae KACC10331. Arch. Phytopath. Plant Protect..

[28] Auster L., Sutton M., Gwin M.C., Nitkin C., Bonfield T.L. (2019). Optimization of *In Vitro Mycobacterium avium* and Mycobacterium intracellulare Growth Assays for Therapeutic Development. Microorganisms.

[29] Shoukat K., Rasheed N., Sajid M. (2012). Subtractive genome analysis for *In silico* identification and characterization of novel drug targets IN C. trachomatis STRAIN D/UW-3/Cx. Int. J. Curr. Res..

[30] Koteswara R.G., Nagamalleswara R.K., Phani R., Krishna B., Aravind S. (2010). *In silico* identification of potential therapeutic targets *inclostridium botulinum* by the approach subtractive genomics. Int. J. Bioinform. Res..

[31] Sharma V., Gupta P., Dixit A. (2008). *In silico* identification of putative drug targets from different metabolic pathways of *Aeromonas hydrophila*. Silico Biol..

[32] Hanson R.S., Hanson T.E. (1996). Methanotrophic bacteria. Microbiol. Rev..

[33] Dalton H., Kelly J.W., Baldwin T.O. (1991). Structure and Mechanism of Action of the Enzyme(s) Involved in Methane Oxidation. Applications of Enzyme Biotechnology.

[34] Beste D.J., Noh K., Niedenfuhr S., Mendum T.A., Hawkins N.D., Ward J.L., Beale M.H., Wiechert W., McFadden J. (2013). 13C-flux spectral analysis of host-pathogen metabolism reveals a mixed diet for intracellular *Mycobacterium tuberculosis*. Chem. Biol..

[35] Gouzy A., Larrouy-Maumus G., Bottai D., Levillain F., Dumas A., Wallach J.B., Caire-Brandli I., De Chastellier C., Wu T.D., Poincloux R. (2014). *Mycobacterium tuberculosis* exploits asparagine to assimilate nitrogen and resist acid stress during infection. PLoS Pathog..

[36] Gouzy A., Larrouy-Maumus G., Wu T.D., Peixoto A., Levillain F., Lugo-Villarino G., Guerquin-Kern J.L., De Carvalho L.P., Poquet Y., Neyrolles O. (2013). *Mycobacterium tuberculosis* nitrogen assimilation and host colonization require aspartate. Nat. Chem. Biol..

[37] Tullius M.V., Harth G., Horwitz M.A. (2003). Glutamine synthetase GlnA1 is essential for growth of *Mycobacterium tuberculosis* in human THP-1 macrophages and guinea pigs. Infect. Immun..

[38] Gillner D.M., Becker D.P., Holz R.C. (2013). Lysine biosynthesis in bacteria: A metallodesuccinylase as a potential antimicrobial target. J. Biol. Inorg. Chem.

[39] Mandal R.S., Das S. (2015). In silico approach towards identification of potential inhibitors of *Helicobacter pylori* DapE. J. Biomol. Struct. Dyn..

[40] Halouska S., Fenton R.J., Zinniel D.K., Marshall D.D., Barletta R.G., Powers R. (2014). Metabolomics analysis identifies D-Alanine-D-Alanine ligase as the primary lethal target of D-Cycloserine in mycobacteria. J. Proteome Res..

[41] Qiu W., Zheng X., Wei Y., Zhou X., Zhang K., Wang S., Cheng L., Li Y., Ren B., Xu X. (2016). D-Alanine metabolism is essential for growth and biofilm formation of *Streptococcus mutans*. Mol. Oral Microbiol..

[42] Silver L.L. (2016). Appropriate Targets for Antibacterial Drugs. Cold Spring Harb. Perspect. Med..

[43] Caffrey C.R., Rohwer A., Oellien F., Marhöfer R.J., Braschi S., Oliveira G., McKerrow J.H., Selzer P.M. (2009). A comparative chemogenomics strategy to predict potential drug targets in the metazoan pathogen, *Schistosoma mansoni*. PLoS ONE.

[44] Consortium U. (2015). UniProt: A hub for protein information. Nucleic Acids Res..

[45] Fu L., Niu B., Zhu Z., Wu S., Li W. (2012). CD-HIT: Accelerated for clustering the next-generation sequencing data. Bioinformatics.

[46] Tao T. (2008). Standalone BLAST Setup for Unix.

[47] Kerfeld C.A., Scott K.M. (2011). Using BLAST to teach “E-value-tionary” concepts. PLoS Biol..

[48] Gao F., Luo H., Zhang C.-T., Zhang R. (2015). Gene essentiality analysis based on DEG 10, an updated database of essential genes. Gene Essentiality.

[49] Kanehisa M., Furumichi M., Tanabe M., Sato Y., Morishima K. (2016). KEGG: New perspectives on genomes, pathways, diseases and drugs. Nucleic Acids Res..

[50] Moriya Y., Itoh M., Okuda S., Yoshizawa A.C., Kanehisa M. (2007). KAAS: An automatic genome annotation and pathway reconstruction server. Nucleic Acids Res..

[51] Yu N.Y., Wagner J.R., Laird M.R., Melli G., Rey S., Lo R., Dao P., Sahinalp S.C., Ester M., Foster L.J. (2010). PSORTb 3.0: Improved protein subcellular localization prediction with refined localization subcategories and predictive capabilities for all prokaryotes. Bioinformatics.

[52] Li Y.H., Xu J.Y., Tao L., Li X.F., Li S., Zeng X., Chen S.Y., Zhang P., Qin C., Zhang C. (2016). SVM-Prot 2016: A web-server for machine learning prediction of protein functional families from sequence irrespective of similarity. PLoS ONE.

[53] Wishart D.S., Feunang Y.D., Guo A.C., Lo E.J., Marcu A., Grant J.R., Sajed T., Johnson D., Li C., Sayeeda Z. (2017). DrugBank 5.0: A major update to the DrugBank database for 2018. Nucleic Acids Res..

