# Supplementary material for: Genome Subtraction and Comparison for the Identification of Novel Drug Targets against Mycobacterium avium subsp. hominissuis
Source: Pathogens. 2020 May 12;9(5):368. doi: 10.3390/pathogens9050368 (PMC7281720; doi:10.3390/pathogens9050368)
Supplement: Supplementary file 1 [file pathogens-09-00368-s001.zip › pathogens-729691-supplementary/Supplementary file/Supplementary table S1.pdf]

Supplementary table 1.

SVM RESULT BAR GRAPH

Predicted Protein Family Name
Zinc-binding
Transferases
Structural protein
Sodium-binding
RNA-binding Proteins
Photosynthesis
P-ATPase) family
Oxidoreductases
mRNA-binding Proteins
Metal-binding
Manganese-binding
Major facilitator family
Magnesium-binding
Lyases
Lipoprotein
All lipid-binding proteins
Ligases
Isomerases
Iron-binding
Immune response
Hydrolases - Acting on peptide bonds (Peptidases)
Hydrolases
Group Translocators
Electrochemical Potential-driven transporters
DNA replication
DNA repair
DNA recombination
DNA condensation
Copper-binding
Chlorophyll biosynthesis
Chlorophyll
All lipid-binding proteins
All DNA-binding
7 transmembrane receptor (Secretin family)
7 transmembrane receptor (metabotropic glutamate family)
G protein coupled receptors
7 transmembrane receptor (rhodopsin family and chemoreceptor)
rRNA-binding proteins
Nuclear receptors

TC3.A Primary Active Transporters - P-P-bond-hydrolysis-driven transporters
Nickel-binding
Porin
Sigma factor
TC3.A.5 Type II (general) secretory pathway (IISP) family
Lipid metabolism
TC8.A Accessory Factors Involved in Transport - Auxiliary transport proteins
Cell adhesion
Lipid degradation
Lipid transport
Plant defense
Lipid synthesis
Lipopolysaccharide biosynthesis
EC6.6 Forming nitrogenmetal bonds
Photorespiration
Primary Active Transporters - Oxidoreduction-driven transporters
TC9.B Incompletely Characterized Transport Systems

			TC9.B Incompletely Character
**MAH-TH135**	**MAH-OCU466**	**MAH-A5**	Primary Active Transporters - Oxidoreduc
70	39	67	
20	13	26	EC6.6 Form
1		1	Lipopoly
		1	
2			
3	2	4	
2		1	
2	4	5	
1		3	TC8.A Accessory Factors Involved in Transport - Auxi
6	2	2	
6	2	5	TC3.A.5 Type II (general) secreto
1		1	
2	2	4	
8	1	4	
8	3	6	TC3.A Primary Active Transporters - P-P-bond-hydro
15			
1		1	
1		1	7 transmembrane receptor (rhodopsin fa
21	9	14	G p
1	1	2	7 transmembrane receptor (metab
1		1	7 transmembrane r
13	12	15	
2			
5	6	1	
1	2	2	
27	24	38	
5	1	2	
1			
	2	2	
21	11	16	
1			Electrochemical Pote
	11	17	
1	3	1	
1	1		Hydrolases - Acting on pep
1	2	1	
1		1	
	1	2	
2		2	
	1		

1	3	1
1		
1		
		1
2	1	2
2	2	5
1	2	3
1		
1	2	2
	1	
1		
2	1	2
1		1
	1	1
	1	
	1	
	2	

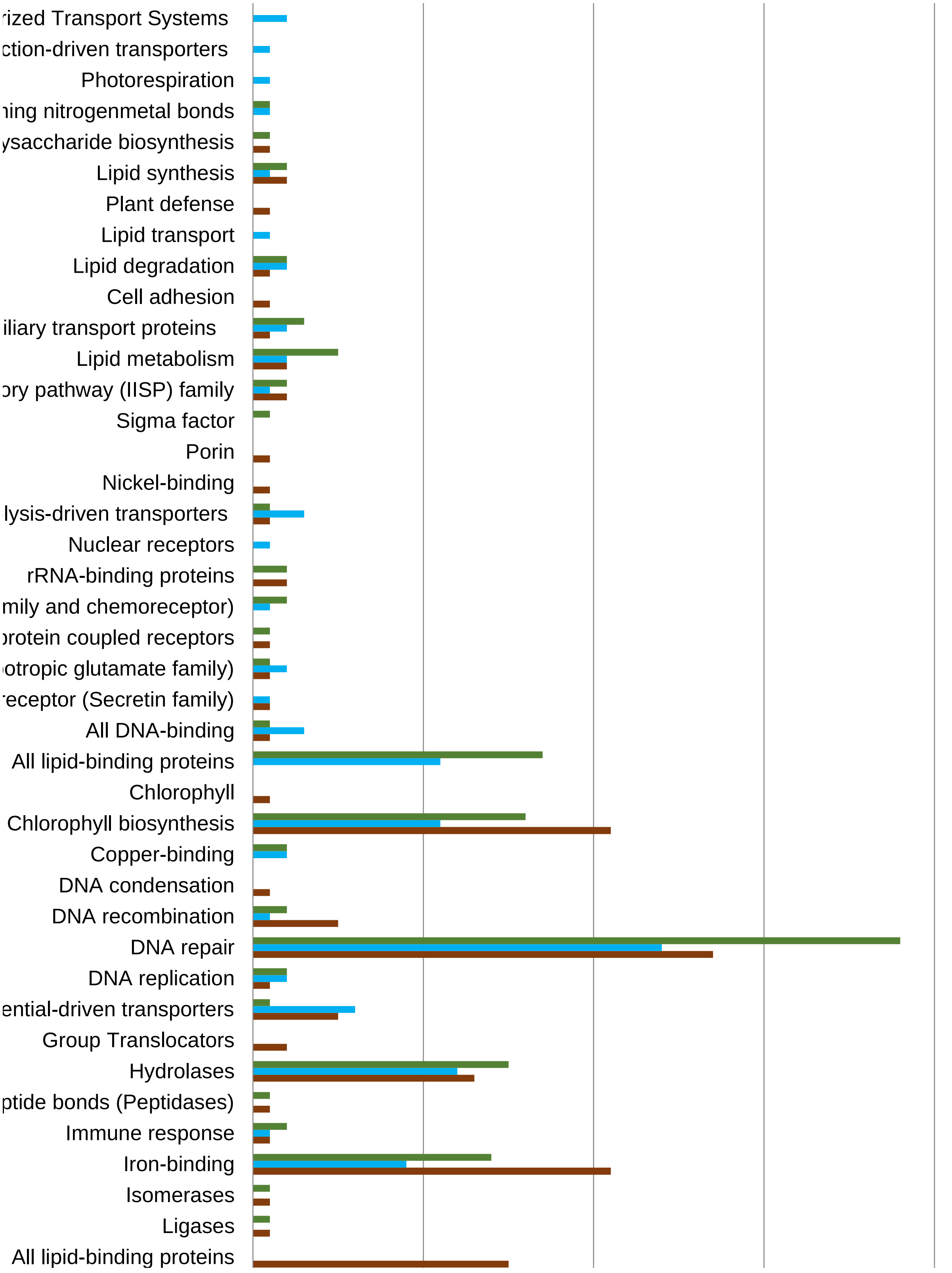
ized Transport Systems
ction-driven transporters
Photorespiration
ning nitrogenmetal bonds
ysaccharide biosynthesis
Lipid synthesis
Plant defense
Lipid transport
Lipid degradation
Cell adhesion
iliary transport proteins
Lipid metabolism
ory pathway (IISP) family
Sigma factor
Porin
Nickel-binding
lysis-driven transporters
Nuclear receptors
rRNA-binding proteins
mily and chemoreceptor)
protein coupled receptors
otropic glutamate family)
receptor (Secretin family)
All DNA-binding
All lipid-binding proteins
Chlorophyll
Chlorophyll biosynthesis
Copper-binding
DNA condensation
DNA recombination
DNA repair
DNA replication
ential-driven transporters
Group Translocators
Hydrolases
ptide bonds (Peptidases)
Immune response
Iron-binding
Isomerases
Ligases
All lipid-binding proteins

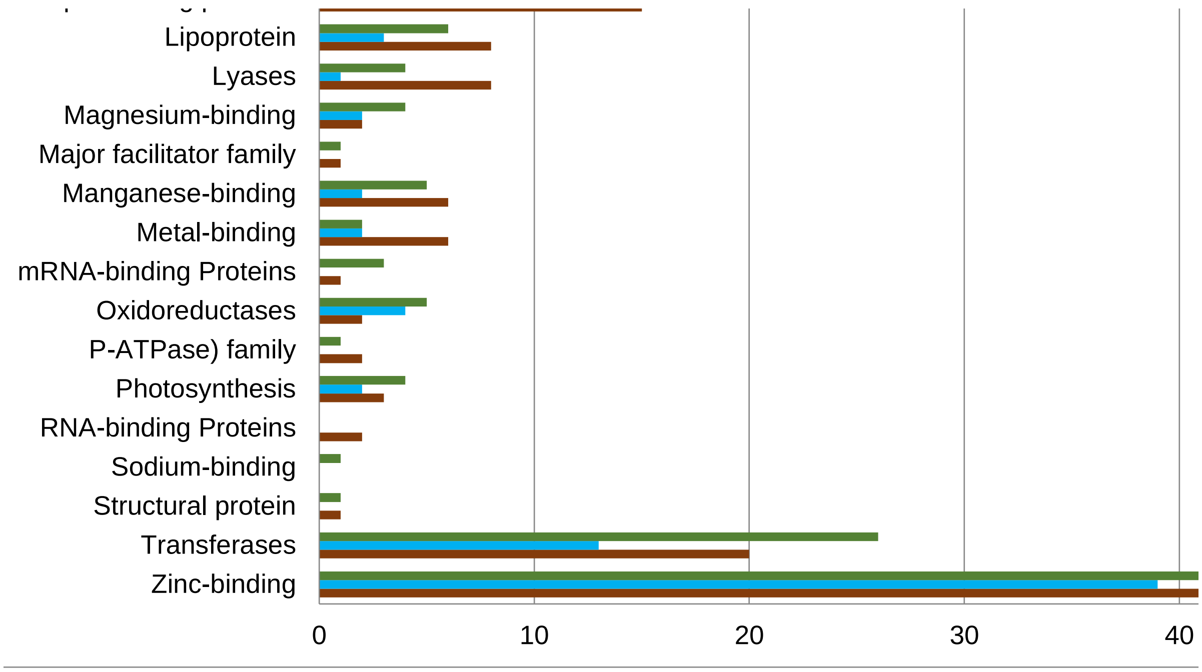
Lipoprotein
Lyases
Magnesium-binding
Major facilitator family
Manganese-binding
Metal-binding
mRNA-binding Proteins
Oxidoreductases
P-ATPase) family
Photosynthesis
RNA-binding Proteins
Sodium-binding
Structural protein
Transferases
Zinc-binding
0
10
20
30
40

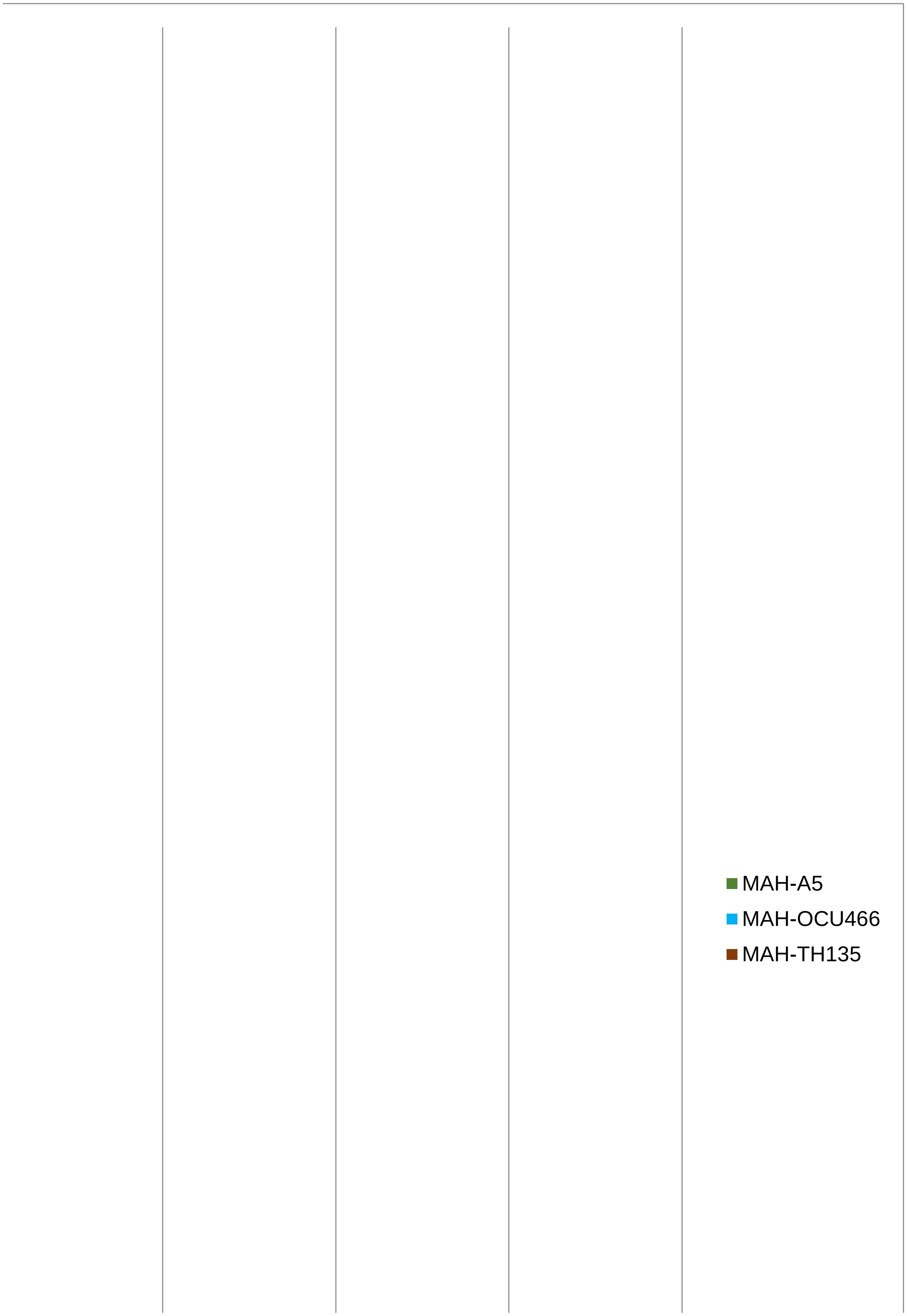
MAH-A5
MAH-OCU466
MAH-TH135

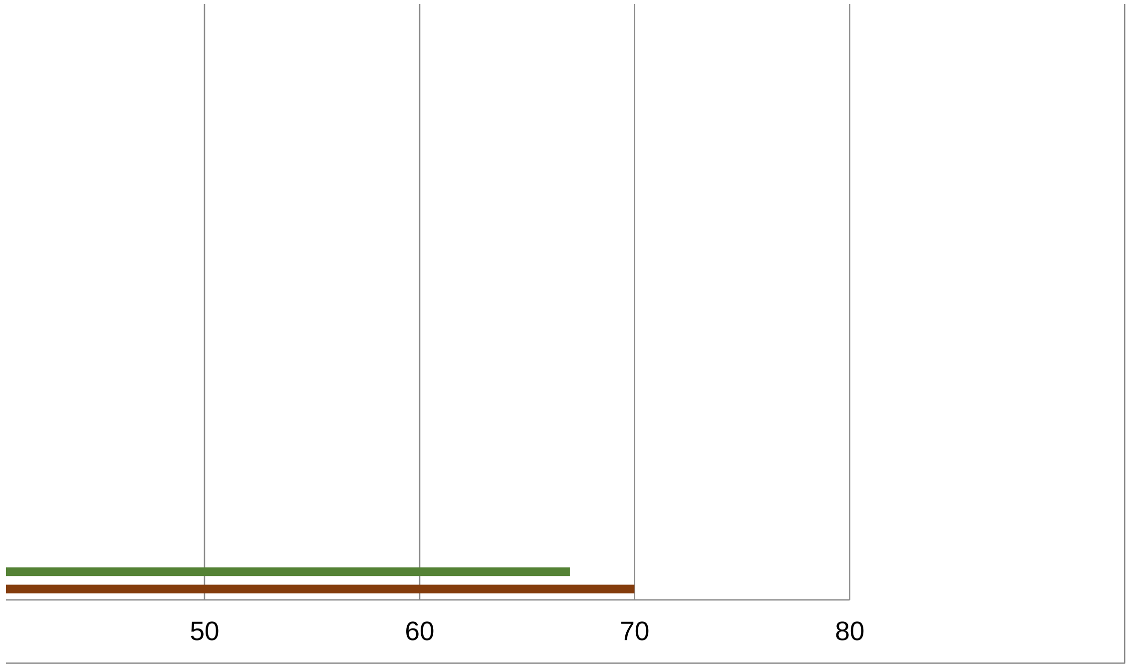
50
60
70
80
