# Supplementary material for: Genome Subtraction and Comparison for the Identification of Novel Drug Targets against Mycobacterium avium subsp. hominissuis
Source: Pathogens. 2020 May 12;9(5):368. doi: 10.3390/pathogens9050368 (PMC7281720; doi:10.3390/pathogens9050368)
Supplement: Supplementary file 1 [file pathogens-09-00368-s001.zip › pathogens-729691-supplementary/Supplementary file/Supplementary Table S5.docx]

**Supplementary Table S5.** The list of unique metabolic pathways commonly present in strains of *M. avium* with corresponding KEGG pathway IDs

| Metabolism | Pathways |
| --- | --- |
| **Lipid Metabolism** | Cutin, suberine and wax biosynthesis [PATH:ko00073]  Secondary bile acid biosynthesis [PATH:ko00121] |
| **Energy Metabolism** | Photosynthesis [PATH:ko00195]  Methane metabolism [PATH:ko00680]  Carbon fixation in photosynthetic organisms [PATH:ko00710]  Carbon fixation pathways in prokaryotes [PATH:ko00720]  Photosynthesis - antenna proteins [PATH:ko00196] |
| **Biosynthesis of other secondary metabolites** | Puromycin biosynthesis [PATH:ko00231]  Aflatoxin biosynthesis [PATH:ko00254]  Monobactam biosynthesis [PATH:ko00261]  Penicillin and cephalosporin biosynthesis [PATH:ko00311]  Clavulanic acid biosynthesis [PATH:ko00331]  Carbapenem biosynthesis [PATH:ko00332]  Novobiocin biosynthesis [PATH:ko00401]  Benzoxazinoid biosynthesis [PATH:ko00402]  Indole diterpene alkaloid biosynthesis [PATH:ko00403]  Staurosporine biosynthesis [PATH:ko00404]  Phenazine biosynthesis [PATH:ko00405]  Flavonoid biosynthesis [PATH:ko00941]  Anthocyanin biosynthesis [PATH:ko00942]  Isoflavonoid biosynthesis [PATH:ko00943]  Flavone and flavonol biosynthesis [PATH:ko00944]  Stilbenoid, diarylheptanoid and gingerol biosynthesis [PATH:ko00945]  Isoquinoline alkaloid biosynthesis [PATH:ko00950]  Tropane, piperidine and pyridine alkaloid biosynthesis [PATH:ko00960]  Betalain biosynthesis [PATH:ko00965]  Glucosinolate biosynthesis [PATH:ko00966]  Biosynthesis of secondary metabolites - unclassified [PATH:ko00999]  Acarbose and validamycin biosynthesis [PATH:ko00525]  Streptomycin biosynthesis [PATH:ko00521]  Indole alkaloid biosynthesis [PATH:ko00901]  Prodigiosin biosynthesis [PATH:ko00333]  Acridone alkaloid biosynthesis [PATH:ko01058] |
| **Amino acid metabolism** | Lysine biosynthesis [PATH:ko00300]  Metabolism of other amino acids  Cyanoamino acid metabolism [PATH:ko00460]  D-Alanine metabolism [PATH:ko00473]  Xenobiotics biodegradation and metabolism  Chlorocyclohexane and chlorobenzene degradation [PATH:ko00361]  Benzoate degradation [PATH:ko00362]  Bisphenol degradation [PATH:ko00363]  Fluorobenzoate degradation [PATH:ko00364]  Furfural degradation [PATH:ko00365]  Dioxin degradation [PATH:ko00621]  Xylene degradation [PATH:ko00622]  Toluene degradation [PATH:ko00623]  Polycyclic aromatic hydrocarbon degradation [PATH:ko00624]  Chloroalkane and chloroalkene degradation [PATH:ko00625]  Naphthalene degradation [PATH:ko00626]  Aminobenzoate degradation [PATH:ko00627]  Nitrotoluene degradation [PATH:ko00633]  Ethylbenzene degradation [PATH:ko00642]  Styrene degradation [PATH:ko00643]  Caprolactam degradation [PATH:ko00930]  Phenylpropanoid biosynthesis [PATH:ko00940]  Atrazine degradation [PATH:ko00791]  Steroid degradation [PATH:ko00984] |
| **Metabolism of terpenoids and polyketides** | Monoterpenoid biosynthesis [PATH:ko00902]  Limonene and pinene degradation [PATH:ko00903]  Diterpenoid biosynthesis [PATH:ko00904]  Brassinosteroid biosynthesis [PATH:ko00905]  Carotenoid biosynthesis [PATH:ko00906]  Zeatin biosynthesis [PATH:ko00908]  Sesquiterpenoid and triterpenoid biosynthesis [PATH:ko00909]  Geraniol degradation [PATH:ko00281]  Biosynthesis of 12-, 14- and 16-membered macrolides [PATH:ko00522}  Polyketide sugar unit biosynthesis [PATH:ko00523]  Insect hormone biosynthesis [PATH:ko00981]  Biosynthesis of ansamycins [PATH:ko01051]  Type I polyketide structures [PATH:ko01052]  Biosynthesis of siderophore group nonribosomal peptides [PATH:ko01053]  Nonribosomal peptide structures [PATH:ko01054]  Biosynthesis of vancomycin group antibiotics [PATH:ko01055]  Biosynthesis of type II polyketide backbone [PATH:ko01056]  Biosynthesis of type II polyketide products [PATH:ko01057]  Tetracycline biosynthesis [PATH:ko00253]  Biosynthesis of enediyne antibiotics [PATH:ko01059] |
| **Glycan biosynthesis and metabolism** | Various types of N-glycan biosynthesis [PATH:ko00513]  Lipopolysaccharide biosynthesis [PATH:ko00540]  Peptidoglycan biosynthesis [PATH:ko00550]  Lipoarabinomannan (LAM) biosynthesis [PATH:ko00571]  Arabinogalactan biosynthesis - Mycobacterium [PATH:ko00572] |
| **Carbohydrate metabolism** | C5-Branched dibasic acid metabolism [PATH:ko00660] |
| **Drug resistance: Antimicrobial** | beta-Lactam resistance [PATH:ko01501]  Vancomycin resistance [PATH:ko01502]  Cationic antimicrobial peptide (CAMP) resistance [PATH:ko01503] |
| **Signal transduction** | Two-component system [PATH:ko02020]  MAPK signaling pathway - yeast [PATH:ko04011]  MAPK signaling pathway - fly [PATH:ko04013]  MAPK signaling pathway - plant [PATH:ko04016]  Plant hormone signal transduction [PATH:ko04075]  Hedgehog signaling pathway - fly [PATH:ko04341]  Hippo signaling pathway - fly [PATH:ko04391] |
| **Cellular community - prokaryotes** | Quorum sensing [PATH:ko02024]  Biofilm formation - Escherichia coli [PATH:ko02026]  Biofilm formation - Vibrio cholerae [PATH:ko05111] |
| **Cell motility** | Bacterial chemotaxis [PATH:ko02030]  Flagellar assembly [PATH:ko02040] |
| **Membrane transport** | Phosphotransferase system (PTS) [PATH:ko02060]  Bacterial secretion system [PATH:ko03070] |
| **Cell growth and death** | Cell cycle - yeast [PATH:ko04111]  Cell cycle - Caulobacter [PATH:ko04112]  Meiosis - yeast [PATH:ko04113]  Apoptosis - fly [PATH:ko04214] |
| **Transport and catabolism** | Autophagy - yeast [PATH:ko04138]  Mitophagy - yeast [PATH:ko04139] |
| **Environmental adaptation** | Plant-pathogen interaction [PATH:ko04626]  Circadian rhythm - fly [PATH:ko04711]  Circadian rhythm - plant [PATH:ko04712] |
| **Development** | Dorso-ventral axis formation [PATH:ko04320] |
| **Immune system** | Toll and Imd signaling pathway [PATH:ko04624] |
|  |  |
| **Sensory system** | Phototransduction - fly [PATH:ko04745] |
| **Aging** | Longevity regulating pathway - worm [PATH:ko04212] |
