# Supplementary material for: Genome Subtraction and Comparison for the Identification of Novel Drug Targets against Mycobacterium avium subsp. hominissuis
Source: Pathogens. 2020 May 12;9(5):368. doi: 10.3390/pathogens9050368 (PMC7281720; doi:10.3390/pathogens9050368)
Supplement: Supplementary file 1 [file pathogens-09-00368-s001.zip › pathogens-729691-supplementary/Supplementary file/supplementary table S2.doc]

Supplementary table 2.

**ESSENTIAL NON-HOMOLOGOUS PROTEINS IN MAH STRAIN TH135 INVOLVED IN DIFFERENT METABOLIC PATHWAYS AND OTHER CELLULAR ACTIVITIES**

**IDs of protein, KEGG Orthology number (KO) Gene and Protein Names:**

**Metabolism:**

**1. Carbohydrate Metabolism (Glycolysis/Gluconeogenesis, Citrate cycle (TCA cycle), Pentose phosphate pathway, Fructose and mannose metabolism, Galactose metabolism, Ascorbate and aldarate metabolism, Starch and sucrose metabolism, Amino sugar and nucleotide sugar metabolism, Pyruvate metabolism, Glyoxylate and dicarboxylate metabolism, Propanoate metabolism, Butanoate metabolism)**

1. tr|T2GLS4|T2GLS4_MYCHT	K01858 INO1, ISYNA1	myo-inositol-1-phosphate synthase [EC:5.5.1.4]
2. tr|T2GLT3|T2GLT3_MYCHT	K01854 glf	UDP-galactopyranose mutase [EC:5.4.99.9]
3. tr|T2GLT3|T2GLT3_MYCHT	K01854 glf	UDP-galactopyranose mutase [EC:5.4.99.9]
4. tr|T2GMW2|T2GMW2_MYCHT	K00123 fdoG, fdhF, fdwA	formate dehydrogenase major subunit [EC:1.17.1.9]
5. tr|T2GMZ4|T2GMZ4_MYCHT	K00045 E1.1.1.67, mtlK	mannitol 2-dehydrogenase [EC:1.1.1.67]
6. tr|T2GN05|T2GN05_MYCHT	K01810 GPI, pgi	glucose-6-phosphate isomerase [EC:5.3.1.9]
7. tr|T2GN05|T2GN05_MYCHT	K01810 GPI, pgi	glucose-6-phosphate isomerase [EC:5.3.1.9]
8. tr|T2GN05|T2GN05_MYCHT	K01810 GPI, pgi	glucose-6-phosphate isomerase [EC:5.3.1.9]
9. tr|T2GN05|T2GN05_MYCHT	K01810 GPI, pgi	glucose-6-phosphate isomerase [EC:5.3.1.9]
10. tr|T2GNA0|T2GNA0_MYCHT	K00124 fdoH, fdsB	formate dehydrogenase iron-sulfur subunit
11. tr|T2GNG3|T2GNG3_MYCHT	K00697 otsA	trehalose 6-phosphate synthase [EC:2.4.1.15 2.4.1.347]
12. tr|T2GP16|T2GP16_MYCHT	K03417 prpB	methylisocitrate lyase [EC:4.1.3.30]
13. tr|T2GP56|T2GP56_MYCHT	K01649 leuA, IMS	2-isopropylmalate synthase [EC:2.3.3.13]
14. tr|T2GP80|T2GP80_MYCHT	K00123 fdoG, fdhF, fdwA	formate dehydrogenase major subunit [EC:1.17.1.9]
15. tr|T2GPP7|T2GPP7_MYCHT	K01808 rpiB	ribose 5-phosphate isomerase B [EC:5.3.1.6]
16. tr|T2GPP7|T2GPP7_MYCHT	K01808 rpiB	ribose 5-phosphate isomerase B [EC:5.3.1.6]
17. tr|T2GQC7|T2GQC7_MYCHT	K00024 mdh	malate dehydrogenase [EC:1.1.1.37]
18. tr|T2GQC7|T2GQC7_MYCHT	K00024 mdh	malate dehydrogenase [EC:1.1.1.37]
19. tr|T2GQC7|T2GQC7_MYCHT	K00024 mdh	malate dehydrogenase [EC:1.1.1.37]
20. tr|T2GQL9|T2GQL9_MYCHT	K00790 murA	UDP-N-acetylglucosamine 1-carboxyvinyltransferase [EC:2.5.1.7]
21. tr|T2GR43|T2GR43_MYCHT	K00174 korA, oorA, oforA	2-oxoglutarate/2-oxoacid ferredoxin oxidoreductase subunit alpha [EC:1.2.7.3 1.2.7.11]
22. tr|T2GR43|T2GR43_MYCHT	K00174 korA, oorA, oforA	2-oxoglutarate/2-oxoacid ferredoxin oxidoreductase subunit alpha [EC:1.2.7.3 1.2.7.11]
23. tr|T2GR43|T2GR43_MYCHT	K00174 korA, oorA, oforA	2-oxoglutarate/2-oxoacid ferredoxin oxidoreductase subunit alpha [EC:1.2.7.3 1.2.7.11]
24. tr|T2GR43|T2GR43_MYCHT	K00174 korA, oorA, oforA	2-oxoglutarate/2-oxoacid ferredoxin oxidoreductase subunit alpha [EC:1.2.7.3 1.2.7.11]
25. tr|T2GR73|T2GR73_MYCHT	K02446 glpX	fructose-1,6-bisphosphatase II [EC:3.1.3.11]
26. tr|T2GR73|T2GR73_MYCHT	K02446 glpX	fructose-1,6-bisphosphatase II [EC:3.1.3.11]
27. tr|T2GR73|T2GR73_MYCHT	K02446 glpX	fructose-1,6-bisphosphatase II [EC:3.1.3.11]
28. tr|T2GRA5|T2GRA5_MYCHT	K01006 ppdK	pyruvate, orthophosphate dikinase [EC:2.7.9.1]
29. tr|T2GRA6|T2GRA6_MYCHT	K01681 ACO, acnA	aconitate hydratase [EC:4.2.1.3]
30. tr|T2GRA6|T2GRA6_MYCHT	K01681 ACO, acnA	aconitate hydratase [EC:4.2.1.3]
31. tr|T2GRF4|T2GRF4_MYCHT	K00175 korB, oorB, oforB	2-oxoglutarate/2-oxoacid ferredoxin oxidoreductase subunit beta [EC:1.2.7.3 1.2.7.11]
32. tr|T2GRF4|T2GRF4_MYCHT	K00175 korB, oorB, oforB	2-oxoglutarate/2-oxoacid ferredoxin oxidoreductase subunit beta [EC:1.2.7.3 1.2.7.11]
33. tr|T2GRF4|T2GRF4_MYCHT	K00175 korB, oorB, oforB	2-oxoglutarate/2-oxoacid ferredoxin oxidoreductase subunit beta [EC:1.2.7.3 1.2.7.11]
34. tr|T2GRF4|T2GRF4_MYCHT	K00175 korB, oorB, oforB	2-oxoglutarate/2-oxoacid ferredoxin oxidoreductase subunit beta [EC:1.2.7.3 1.2.7.11]
35. tr|T2GRH6|T2GRH6_MYCHT	K01704 leuD, IPMI-S	3-isopropylmalate/(R)-2-methylmalate dehydratase small subunit [EC:4.2.1.33 4.2.1.35]
36. tr|T2GRM1|T2GRM1_MYCHT	K00873 PK, pyk	pyruvate kinase [EC:2.7.1.40]
37. tr|T2GRM1|T2GRM1_MYCHT	K00873 PK, pyk	pyruvate kinase [EC:2.7.1.40]
38. tr|T2GRY6|T2GRY6_MYCHT	K16147 glgE	starch synthase (maltosyl-transferring) [EC:2.4.99.16]
39. tr|T2GSP0|T2GSP0_MYCHT	K01783 rpe, RPE	ribulose-phosphate 3-epimerase [EC:5.1.3.1]
40. tr|T2GSP0|T2GSP0_MYCHT	K01783 rpe, RPE	ribulose-phosphate 3-epimerase [EC:5.1.3.1]
41. tr|T2GSV2|T2GSV2_MYCHT	K00249 ACADM, acd	acyl-CoA dehydrogenase [EC:1.3.8.7]
42. tr|T2GTN9|T2GTN9_MYCHT	K01595 ppc	phosphoenolpyruvate carboxylase [EC:4.1.1.31]
43. tr|T2GTW5|T2GTW5_MYCHT	K00705 malQ	4-alpha-glucanotransferase [EC:2.4.1.25]
44. tr|T2GTY2|T2GTY2_MYCHT	K01637 E4.1.3.1, aceA	isocitrate lyase [EC:4.1.3.1]
45. tr|T2GU18|T2GU18_MYCHT	K06044 treY, glgY	(1->4)-alpha-D-glucan 1-alpha-D-glucosylmutase [EC:5.4.99.15]
46. tr|T2GUY6|T2GUY6_MYCHT	K01638 aceB, glcB	malate synthase [EC:2.3.3.9]
47. tr|T2GUY6|T2GUY6_MYCHT	K01638 aceB, glcB	malate synthase [EC:2.3.3.9]
48. tr|T2GV99|T2GV99_MYCHT	K00865 glxK, garK	glycerate 2-kinase [EC:2.7.1.165]
49. tr|T2GVH4|T2GVH4_MYCHT	K00121 frmA, ADH5, adhC	S-(hydroxymethyl)glutathione dehydrogenase / alcohol dehydrogenase [EC:1.1.1.284 1.1.1.1]
50. tr|T2GVQ2|T2GVQ2_MYCHT	K00886 ppgK	polyphosphate glucokinase [EC:2.7.1.63]
51. tr|T2GVQ2|T2GVQ2_MYCHT	K00886 ppgK	polyphosphate glucokinase [EC:2.7.1.63]
52. tr|T2GW31|T2GW31_MYCHT	K00031 IDH1, IDH2, icd	isocitrate dehydrogenase [EC:1.1.1.42]
53. tr|T2GW88|T2GW88_MYCHT	K01087 otsB	trehalose 6-phosphate phosphatase [EC:3.1.3.12]
54. tr|T2GWJ1|T2GWJ1_MYCHT	K01653 E2.2.1.6S, ilvH, ilvN	acetolactate synthase I/III small subunit [EC:2.2.1.6]
55. tr|T2GWJ1|T2GWJ1_MYCHT	K01653 E2.2.1.6S, ilvH, ilvN	acetolactate synthase I/III small subunit [EC:2.2.1.6]
56. tr|T2GWX2|T2GWX2_MYCHT	K00849 galK	galactokinase [EC:2.7.1.6]
57. tr|T2GWX2|T2GWX2_MYCHT	K00849 galK	galactokinase [EC:2.7.1.6]
58. tr|T2GX92|T2GX92_MYCHT	K00074 paaH, hbd, fadB, mmgB	3-hydroxybutyryl-CoA dehydrogenase [EC:1.1.1.157]
59. tr|T2GXD9|T2GXD9_MYCHT	K13788 pta	phosphate acetyltransferase [EC:2.3.1.8]
60. tr|T2GXD9|T2GXD9_MYCHT	K13788 pta	phosphate acetyltransferase [EC:2.3.1.8]
61. tr|T2GXF3|T2GXF3_MYCHT	K01704 leuD, IPMI-S	3-isopropylmalate/(R)-2-methylmalate dehydratase small subunit [EC:4.2.1.33 4.2.1.35]
62. tr|T2GXI6|T2GXI6_MYCHT	K16149 K16149	1,4-alpha-glucan branching enzyme [EC:2.4.1.18]
63. tr|T2GYF4|T2GYF4_MYCHT	K00966 GMPP	mannose-1-phosphate guanylyltransferase [EC:2.7.7.13]
64. tr|T2GYF4|T2GYF4_MYCHT	K00966 GMPP	mannose-1-phosphate guanylyltransferase [EC:2.7.7.13]
65. tr|T2GYH3|T2GYH3_MYCHT	K00075 murB	UDP-N-acetylmuramate dehydrogenase [EC:1.3.1.98]
66. tr|T2GYW7|T2GYW7_MYCHT	K00045 E1.1.1.67, mtlK	mannitol 2-dehydrogenase [EC:1.1.1.67]
67. tr|T2GYX6|T2GYX6_MYCHT	K00925 ackA	acetate kinase [EC:2.7.2.1]
68. tr|T2GYX6|T2GYX6_MYCHT	K00925 ackA	acetate kinase [EC:2.7.2.1]
69. tr|T2GZD4|T2GZD4_MYCHT	K01637 E4.1.3.1, aceA	isocitrate lyase [EC:4.1.3.1]
70. tr|T2GZH9|T2GZH9_MYCHT	K00249 ACADM, acd	acyl-CoA dehydrogenase [EC:1.3.8.7]
71. tr|T2GZQ6|T2GZQ6_MYCHT	K05349 bglX	beta-glucosidase [EC:3.2.1.21]
72. tr|T2GZS7|T2GZS7_MYCHT	K16146 pep2	maltokinase [EC:2.7.1.175]

2. Energy Metabolism (Oxidative phosphorylation, Photosynthesis, Carbon fixation in photosynthetic organisms, Carbon fixation pathways in prokaryotes, Methane metabolism, Nitrogen metabolism, Sulfur metabolism)

1. tr|T2GMC6|T2GMC6_MYCHT	K01673 cynT, can	carbonic anhydrase [EC:4.2.1.1]
2. tr|T2GME9|T2GME9_MYCHT	K00265 gltB	glutamate synthase (NADPH) large chain [EC:1.4.1.13]
3. tr|T2GMM9|T2GMM9_MYCHT	K15554 ssuC	sulfonate transport system permease protein
4. tr|T2GMQ0|T2GMQ0_MYCHT	K00266 gltD	glutamate synthase (NADPH) small chain [EC:1.4.1.13]
5. tr|T2GMW2|T2GMW2_MYCHT	K00123 fdoG, fdhF, fdwA	formate dehydrogenase major subunit [EC:1.17.1.9]
6. tr|T2GNA0|T2GNA0_MYCHT	K00124 fdoH, fdsB	formate dehydrogenase iron-sulfur subunit
7. tr|T2GNR4|T2GNR4_MYCHT	K11779 fbiC	FO synthase [EC:2.5.1.147 4.3.1.32]
8. tr|T2GNX0|T2GNX0_MYCHT	K00370 narG, narZ, nxrA	nitrate reductase / nitrite oxidoreductase, alpha subunit [EC:1.7.5.1 1.7.99.-]
9. tr|T2GP50|T2GP50_MYCHT	K00957 cysD	sulfate adenylyltransferase subunit 2 [EC:2.7.7.4]
10. tr|T2GP74|T2GP74_MYCHT	K02109 ATPF0B, atpF	F-type H+-transporting ATPase subunit b
11. tr|T2GP74|T2GP74_MYCHT	K02109 ATPF0B, atpF	F-type H+-transporting ATPase subunit b
12. tr|T2GP77|T2GP77_MYCHT	K02114 ATPF1E, atpC	F-type H+-transporting ATPase subunit epsilon
13. tr|T2GP77|T2GP77_MYCHT	K02114 ATPF1E, atpC	F-type H+-transporting ATPase subunit epsilon
14. tr|T2GP80|T2GP80_MYCHT	K00123 fdoG, fdhF, fdwA	formate dehydrogenase major subunit [EC:1.17.1.9]
15. tr|T2GPK4|T2GPK4_MYCHT	K03885 ndh	NADH dehydrogenase [EC:1.6.99.3]
16. tr|T2GPP7|T2GPP7_MYCHT	K01808 rpiB	ribose 5-phosphate isomerase B [EC:5.3.1.6]
17. tr|T2GPX3|T2GPX3_MYCHT	K02047 cysW	sulfate/thiosulfate transport system permease protein
18. tr|T2GQB9|T2GQB9_MYCHT	K00640 cysE	serine O-acetyltransferase [EC:2.3.1.30]
19. tr|T2GQC7|T2GQC7_MYCHT	K00024 mdh	malate dehydrogenase [EC:1.1.1.37]
20. tr|T2GQC7|T2GQC7_MYCHT	K00024 mdh	malate dehydrogenase [EC:1.1.1.37]
21. tr|T2GQC7|T2GQC7_MYCHT	K00024 mdh	malate dehydrogenase [EC:1.1.1.37]
22. tr|T2GQH9|T2GQH9_MYCHT	K00390 cysH	phosphoadenosine phosphosulfate reductase [EC:1.8.4.8 1.8.4.10]
23. tr|T2GQI5|T2GQI5_MYCHT	K01673 cynT, can	carbonic anhydrase [EC:4.2.1.1]
24. tr|T2GQL0|T2GQL0_MYCHT	K02108 ATPF0A, atpB	F-type H+-transporting ATPase subunit a
25. tr|T2GQL0|T2GQL0_MYCHT	K02108 ATPF0A, atpB	F-type H+-transporting ATPase subunit a
26. tr|T2GQN3|T2GQN3_MYCHT	K00392 sir	sulfite reductase (ferredoxin) [EC:1.8.7.1]
27. tr|T2GQS5|T2GQS5_MYCHT	K00459 ncd2, npd	nitronate monooxygenase [EC:1.13.12.16]
28. tr|T2GQY6|T2GQY6_MYCHT	K02110 ATPF0C, atpE	F-type H+-transporting ATPase subunit c
29. tr|T2GQY6|T2GQY6_MYCHT	K02110 ATPF0C, atpE	F-type H+-transporting ATPase subunit c
30. tr|T2GR42|T2GR42_MYCHT	K02259 COX15, ctaA	cytochrome c oxidase assembly protein subunit 15
31. tr|T2GR43|T2GR43_MYCHT	K00174 korA, oorA, oforA	2-oxoglutarate/2-oxoacid ferredoxin oxidoreductase subunit alpha [EC:1.2.7.3 1.2.7.11]
32. tr|T2GR73|T2GR73_MYCHT	K02446 glpX	fructose-1,6-bisphosphatase II [EC:3.1.3.11]
33. tr|T2GR73|T2GR73_MYCHT	K02446 glpX	fructose-1,6-bisphosphatase II [EC:3.1.3.11]
34. tr|T2GRA5|T2GRA5_MYCHT	K01006 ppdK	pyruvate, orthophosphate dikinase [EC:2.7.9.1]
35. tr|T2GRA5|T2GRA5_MYCHT	K01006 ppdK	pyruvate, orthophosphate dikinase [EC:2.7.9.1]
36. tr|T2GRA6|T2GRA6_MYCHT	K01681 ACO, acnA	aconitate hydratase [EC:4.2.1.3]
37. tr|T2GRA7|T2GRA7_MYCHT	K23163 sbp	sulfate/thiosulfate transport system substrate-binding protein
38. tr|T2GRB2|T2GRB2_MYCHT	K00392 sir	sulfite reductase (ferredoxin) [EC:1.8.7.1]
39. tr|T2GRD6|T2GRD6_MYCHT	K15371 GDH2	glutamate dehydrogenase [EC:1.4.1.2]
40. tr|T2GRF2|T2GRF2_MYCHT	K00371 narH, narY, nxrB	nitrate reductase / nitrite oxidoreductase, beta subunit [EC:1.7.5.1 1.7.99.-]
41. tr|T2GRF4|T2GRF4_MYCHT	K00175 korB, oorB, oforB	2-oxoglutarate/2-oxoacid ferredoxin oxidoreductase subunit beta [EC:1.2.7.3 1.2.7.11]
42. tr|T2GRL7|T2GRL7_MYCHT	K02046 cysU	sulfate/thiosulfate transport system permease protein
43. tr|T2GRM7|T2GRM7_MYCHT	K00426 cydB	cytochrome bd ubiquinol oxidase subunit II [EC:7.1.1.7]
44. tr|T2GSK9|T2GSK9_MYCHT	K00390 cysH	phosphoadenosine phosphosulfate reductase [EC:1.8.4.8 1.8.4.10]
45. tr|T2GSP0|T2GSP0_MYCHT	K01783 rpe, RPE	ribulose-phosphate 3-epimerase [EC:5.1.3.1]
46. tr|T2GTN9|T2GTN9_MYCHT	K01595 ppc	phosphoenolpyruvate carboxylase [EC:4.1.1.31]
47. tr|T2GTN9|T2GTN9_MYCHT	K01595 ppc	phosphoenolpyruvate carboxylase [EC:4.1.1.31]
48. tr|T2GTN9|T2GTN9_MYCHT	K01595 ppc	phosphoenolpyruvate carboxylase [EC:4.1.1.31]
49. tr|T2GTX1|T2GTX1_MYCHT	K00459 ncd2, npd	nitronate monooxygenase [EC:1.13.12.16]
50. tr|T2GU28|T2GU28_MYCHT	K03885 ndh	NADH dehydrogenase [EC:1.6.99.3]
51. tr|T2GU69|T2GU69_MYCHT	K00425 cydA	cytochrome bd ubiquinol oxidase subunit I [EC:7.1.1.7]
52. tr|T2GUS7|T2GUS7_MYCHT	K15554 ssuC	sulfonate transport system permease protein
53. tr|T2GUX0|T2GUX0_MYCHT	K03890 qcrA	ubiquinol-cytochrome c reductase iron-sulfur subunit
54. tr|T2GVB6|T2GVB6_MYCHT	K00339 nuoJ	NADH-quinone oxidoreductase subunit J [EC:7.1.1.2]
55. tr|T2GVC0|T2GVC0_MYCHT	K04561 norB	nitric oxide reductase subunit B [EC:1.7.2.5]
56. tr|T2GVE0|T2GVE0_MYCHT	K00330 nuoA	NADH-quinone oxidoreductase subunit A [EC:7.1.1.2]
57. tr|T2GVF0|T2GVF0_MYCHT	K00340 nuoK	NADH-quinone oxidoreductase subunit K [EC:7.1.1.2]
58. tr|T2GVH4|T2GVH4_MYCHT	K00121 frmA, ADH5, adhC	S-(hydroxymethyl)glutathione dehydrogenase / alcohol dehydrogenase [EC:1.1.1.284 1.1.1.1]
59. tr|T2GVR6|T2GVR6_MYCHT	K22468 ppk2	polyphosphate kinase [EC:2.7.4.1]
60. tr|T2GVS7|T2GVS7_MYCHT	K15553 ssuA	sulfonate transport system substrate-binding protein
61. tr|T2GW31|T2GW31_MYCHT	K00031 IDH1, IDH2, icd	isocitrate dehydrogenase [EC:1.1.1.42]
62. tr|T2GW57|T2GW57_MYCHT	K03889 qcrC	ubiquinol-cytochrome c reductase cytochrome c subunit
63. tr|T2GWH4|T2GWH4_MYCHT	K00937 ppk	polyphosphate kinase [EC:2.7.4.1]
64. tr|T2GWR3|T2GWR3_MYCHT	K01725 cynS	cyanate lyase [EC:4.2.1.104]
65. tr|T2GXD9|T2GXD9_MYCHT	K13788 pta	phosphate acetyltransferase [EC:2.3.1.8]
66. tr|T2GXD9|T2GXD9_MYCHT	K13788 pta	phosphate acetyltransferase [EC:2.3.1.8]
67. tr|T2GYQ1|T2GYQ1_MYCHT	K00641 metX	homoserine O-acetyltransferase/O-succinyltransferase [EC:2.3.1.31 2.3.1.46]
68. tr|T2GYX6|T2GYX6_MYCHT	K00925 ackA	acetate kinase [EC:2.7.2.1]
69. tr|T2GYX6|T2GYX6_MYCHT	K00925 ackA	acetate kinase [EC:2.7.2.1]
70. tr|T2GZJ0|T2GZJ0_MYCHT	K03885 ndh	NADH dehydrogenase [EC:1.6.99.3]

3. Lipid Metabolism (Fatty acid biosynthesis, Primary bile acid biosynthesis, Secondary bile acid biosynthesis, Glycerolipid metabolism, Glycerophospholipid metabolism)

1. tr|T2GM93|T2GM93_MYCHT	K00059 fabG, OAR1	3-oxoacyl-[acyl-carrier protein] reductase [EC:1.1.1.100]
2. tr|T2GNQ0|T2GNQ0_MYCHT	K03922 desA2	acyl-[acyl-carrier-protein] desaturase [EC:1.14.19.2]
3. tr|T2GNQ0|T2GNQ0_MYCHT	K03922 desA2	acyl-[acyl-carrier-protein] desaturase [EC:1.14.19.2]
4. tr|T2GP06|T2GP06_MYCHT	K03921 FAB2, SSI2, desA1	acyl-[acyl-carrier-protein] desaturase [EC:1.14.19.2 1.14.19.11 1.14.19.26]
5. tr|T2GP06|T2GP06_MYCHT	K03921 FAB2, SSI2, desA1	acyl-[acyl-carrier-protein] desaturase [EC:1.14.19.2 1.14.19.11 1.14.19.26]
6. tr|T2GP24|T2GP24_MYCHT	K00635 tgs, wax-dgat	diacylglycerol O-acyltransferase / wax synthase [EC:2.3.1.20 2.3.1.75]
7. tr|T2GR15|T2GR15_MYCHT	K00635 tgs, wax-dgat	diacylglycerol O-acyltransferase / wax synthase [EC:2.3.1.20 2.3.1.75]
8. tr|T2GSD4|T2GSD4_MYCHT	K00995 pgsA, PGS1	CDP-diacylglycerol---glycerol-3-phosphate 3-phosphatidyltransferase [EC:2.7.8.5]
9. tr|T2GSL4|T2GSL4_MYCHT	K03921 FAB2, SSI2, desA1	acyl-[acyl-carrier-protein] desaturase [EC:1.14.19.2 1.14.19.11 1.14.19.26]
10. tr|T2GSL4|T2GSL4_MYCHT	K03921 FAB2, SSI2, desA1	acyl-[acyl-carrier-protein] desaturase [EC:1.14.19.2 1.14.19.11 1.14.19.26]
11. tr|T2GSQ8|T2GSQ8_MYCHT	K00635 tgs, wax-dgat	diacylglycerol O-acyltransferase / wax synthase [EC:2.3.1.20 2.3.1.75]
12. tr|T2GSV2|T2GSV2_MYCHT	K00249 ACADM, acd	acyl-CoA dehydrogenase [EC:1.3.8.7]
13. tr|T2GTP1|T2GTP1_MYCHT	K02078 acpP	acyl carrier protein
14. tr|T2GTP4|T2GTP4_MYCHT	K00635 tgs, wax-dgat	diacylglycerol O-acyltransferase / wax synthase [EC:2.3.1.20 2.3.1.75]
15. tr|T2GUA8|T2GUA8_MYCHT	K02371 fabK	enoyl-[acyl-carrier protein] reductase II [EC:1.3.1.9]
16. tr|T2GV46|T2GV46_MYCHT	K18851 fbp	diacylglycerol O-acyltransferase / trehalose O-mycolyltransferase [EC:2.3.1.20 2.3.1.122]
17. tr|T2GV99|T2GV99_MYCHT	K00865 glxK, garK	glycerate 2-kinase [EC:2.7.1.165]
18. tr|T2GVH4|T2GVH4_MYCHT	K00121 frmA, ADH5, adhC	S-(hydroxymethyl)glutathione dehydrogenase / alcohol dehydrogenase [EC:1.1.1.284 1.1.1.1]
19. tr|T2GVV0|T2GVV0_MYCHT	K00635 tgs, wax-dgat	diacylglycerol O-acyltransferase / wax synthase [EC:2.3.1.20 2.3.1.75]
20. tr|T2GW61|T2GW61_MYCHT	K00635 tgs, wax-dgat	diacylglycerol O-acyltransferase / wax synthase [EC:2.3.1.20 2.3.1.75]
21. tr|T2GWB1|T2GWB1_MYCHT	K00803 AGPS, agpS	alkyldihydroxyacetonephosphate synthase [EC:2.5.1.26]
22. tr|T2GWN4|T2GWN4_MYCHT	K00255 ACADL	long-chain-acyl-CoA dehydrogenase [EC:1.3.8.8]
23. tr|T2GY01|T2GY01_MYCHT	K00635 tgs, wax-dgat	diacylglycerol O-acyltransferase / wax synthase [EC:2.3.1.20 2.3.1.75]
24. tr|T2GYE1|T2GYE1_MYCHT	K18851 fbp	diacylglycerol O-acyltransferase / trehalose O-mycolyltransferase [EC:2.3.1.20 2.3.1.122]
25. tr|T2GYL9|T2GYL9_MYCHT	K01613 psd, PISD	phosphatidylserine decarboxylase [EC:4.1.1.65]
26. tr|T2GYU8|T2GYU8_MYCHT	K17103 CHO1, pssA	CDP-diacylglycerol---serine O-phosphatidyltransferase [EC:2.7.8.8]
27. tr|T2GYX1|T2GYX1_MYCHT	K00648 fabH	3-oxoacyl-[acyl-carrier-protein] synthase III [EC:2.3.1.180]
28. tr|T2GZH9|T2GZH9_MYCHT	K00249 ACADM, acd	acyl-CoA dehydrogenase [EC:1.3.8.7]
29. tr|T2GZK8|T2GZK8_MYCHT	K01046 lip, TGL2	triacylglycerol lipase [EC:3.1.1.3]

4. Nucleotide Metabolism (Purine metabolism, Pyrimidine metabolism)

1. tr|T2GME1|T2GME1_MYCHT	K23264 purS	phosphoribosylformylglycinamidine synthase subunit PurS [EC:6.3.5.3]
2. tr|T2GNK3|T2GNK3_MYCHT	K02428 rdgB	XTP/dITP diphosphohydrolase [EC:3.6.1.66]
3. tr|T2GNK8|T2GNK8_MYCHT	K01524 ppx-gppA	exopolyphosphatase / guanosine-5'-triphosphate,3'-diphosphate pyrophosphatase [EC:3.6.1.11 3.6.1.40]
4. tr|T2GP50|T2GP50_MYCHT	K00957 cysD	sulfate adenylyltransferase subunit 2 [EC:2.7.7.4]
5. tr|T2GRM1|T2GRM1_MYCHT	K00873 PK, pyk	pyruvate kinase [EC:2.7.1.40]
6. tr|T2GSU3|T2GSU3_MYCHT	K01129 dgt	dGTPase [EC:3.1.5.1]
7. tr|T2GTH2|T2GTH2_MYCHT	K00945 cmk	CMP/dCMP kinase [EC:2.7.4.25]
8. tr|T2GTM6|T2GTM6_MYCHT	K00856 E2.7.1.20, ADK	adenosine kinase [EC:2.7.1.20]
9. tr|T2GUG7|T2GUG7_MYCHT	K09903 pyrH	uridylate kinase [EC:2.7.4.22]
10. tr|T2GW91|T2GW91_MYCHT	K00088 IMPDH, guaB	IMP dehydrogenase [EC:1.1.1.205]
11. tr|T2GWA9|T2GWA9_MYCHT	K01239 iunH	purine nucleosidase [EC:3.2.2.1]
12. tr|T2GWR2|T2GWR2_MYCHT	K03465 thyX, thy1	thymidylate synthase (FAD) [EC:2.1.1.148]
13. tr|T2GX67|T2GX67_MYCHT	K01524 ppx-gppA	exopolyphosphatase / guanosine-5'-triphosphate,3'-diphosphate pyrophosphatase [EC:3.6.1.11 3.6.1.40]
14. tr|T2GXF5|T2GXF5_MYCHT	K08289 purT	phosphoribosylglycinamide formyltransferase 2 [EC:2.1.2.2]
15. tr|T2GY57|T2GY57_MYCHT	K16839 hpxO	FAD-dependent urate hydroxylase [EC:1.14.13.113]
16. tr|T2GYW1|T2GYW1_MYCHT	K01494 dcd	dCTP deaminase [EC:3.5.4.13]

5. Amino Acid Metabolism (Alanine, aspartate and glutamate metabolism, Glycine, serine and threonine metabolism, Cysteine and methionine metabolism, Valine, leucine and isoleucine biosynthesis, Lysine biosynthesis, Lysine degradation, Arginine and proline metabolism, Histidine metabolism, Tyrosine metabolism, Phenylalanine metabolism, Phenylalanine, Tyrosine and tryptophan biosynthesis)

1. tr|T2GLX0|T2GLX0_MYCHT	K21672 ord	2,4-diaminopentanoate dehydrogenase [EC:1.4.1.12 1.4.1.26]
2. tr|T2GLX0|T2GLX0_MYCHT	K21672 ord	2,4-diaminopentanoate dehydrogenase [EC:1.4.1.12 1.4.1.26]
3. tr|T2GM57|T2GM57_MYCHT	K01919 gshA	glutamate--cysteine ligase [EC:6.3.2.2]
4. tr|T2GME9|T2GME9_MYCHT	K00265 gltB	glutamate synthase (NADPH) large chain [EC:1.4.1.13]
5. tr|T2GMK1|T2GMK1_MYCHT	K05714 mhpC	2-hydroxy-6-oxonona-2,4-dienedioate hydrolase [EC:3.7.1.14]
6. tr|T2GMQ0|T2GMQ0_MYCHT	K00266 gltD	glutamate synthase (NADPH) small chain [EC:1.4.1.13]
7. tr|T2GMR9|T2GMR9_MYCHT	K04518 pheA2	prephenate dehydratase [EC:4.2.1.51]
8. tr|T2GMU4|T2GMU4_MYCHT	K00928 lysC	aspartate kinase [EC:2.7.2.4]
9. tr|T2GMU4|T2GMU4_MYCHT	K00928 lysC	aspartate kinase [EC:2.7.2.4]
10. tr|T2GMU4|T2GMU4_MYCHT	K00928 lysC	aspartate kinase [EC:2.7.2.4]
11. tr|T2GN13|T2GN13_MYCHT	K04517 tyrA2	prephenate dehydrogenase [EC:1.3.1.12]
12. tr|T2GN72|T2GN72_MYCHT	K00133 asd	aspartate-semialdehyde dehydrogenase [EC:1.2.1.11]
13. tr|T2GN72|T2GN72_MYCHT	K00133 asd	aspartate-semialdehyde dehydrogenase [EC:1.2.1.11]
14. tr|T2GN72|T2GN72_MYCHT	K00133 asd	aspartate-semialdehyde dehydrogenase [EC:1.2.1.11]
15. tr|T2GN82|T2GN82_MYCHT	K18911 egtD	L-histidine Nalpha-methyltransferase [EC:2.1.1.44]
16. tr|T2GNL2|T2GNL2_MYCHT	K00549 metE	5-methyltetrahydropteroyltriglutamate--homocysteine methyltransferase [EC:2.1.1.14]
17. tr|T2GP03|T2GP03_MYCHT	K00674 dapD	2,3,4,5-tetrahydropyridine-2,6-dicarboxylate N-succinyltransferase [EC:2.3.1.117]
18. tr|T2GP56|T2GP56_MYCHT	K01649 leuA, IMS	2-isopropylmalate synthase [EC:2.3.3.13]
19. tr|T2GP66|T2GP66_MYCHT	K00872 thrB1	homoserine kinase [EC:2.7.1.39]
20. tr|T2GPA8|T2GPA8_MYCHT	K21672 ord	2,4-diaminopentanoate dehydrogenase [EC:1.4.1.12 1.4.1.26]
21. tr|T2GPA8|T2GPA8_MYCHT	K21672 ord	2,4-diaminopentanoate dehydrogenase [EC:1.4.1.12 1.4.1.26]
22. tr|T2GPJ9|T2GPJ9_MYCHT	K01478 arcA	arginine deiminase [EC:3.5.3.6]
23. tr|T2GQ69|T2GQ69_MYCHT	K00318 PRODH, fadM, putB	proline dehydrogenase [EC:1.5.5.2]
24. tr|T2GQB9|T2GQB9_MYCHT	K00640 cysE	serine O-acetyltransferase [EC:2.3.1.30]
25. tr|T2GQC7|T2GQC7_MYCHT	K00024 mdh	malate dehydrogenase [EC:1.1.1.37]
26. tr|T2GQK2|T2GQK2_MYCHT	K00003 hom	homoserine dehydrogenase [EC:1.1.1.3]
27. tr|T2GQK2|T2GQK2_MYCHT	K00003 hom	homoserine dehydrogenase [EC:1.1.1.3]
28. tr|T2GQK2|T2GQK2_MYCHT	K00003 hom	homoserine dehydrogenase [EC:1.1.1.3]
29. tr|T2GQU7|T2GQU7_MYCHT	K01736 aroC	chorismate synthase [EC:4.2.3.5]
30. tr|T2GR02|T2GR02_MYCHT	K00891 E2.7.1.71, aroK, aroL	shikimate kinase [EC:2.7.1.71]
31. tr|T2GRD6|T2GRD6_MYCHT	K15371 GDH2	glutamate dehydrogenase [EC:1.4.1.2]
32. tr|T2GRD6|T2GRD6_MYCHT	K15371 GDH2	glutamate dehydrogenase [EC:1.4.1.2]
33. tr|T2GRH6|T2GRH6_MYCHT	K01704 leuD, IPMI-S	3-isopropylmalate/(R)-2-methylmalate dehydratase small subunit [EC:4.2.1.33 4.2.1.35]
34. tr|T2GRI4|T2GRI4_MYCHT	K00817 hisC	histidinol-phosphate aminotransferase [EC:2.6.1.9]
35. tr|T2GRI4|T2GRI4_MYCHT	K00817 hisC	histidinol-phosphate aminotransferase [EC:2.6.1.9]
36. tr|T2GRI4|T2GRI4_MYCHT	K00817 hisC	histidinol-phosphate aminotransferase [EC:2.6.1.9]
37. tr|T2GRI4|T2GRI4_MYCHT	K00817 hisC	histidinol-phosphate aminotransferase [EC:2.6.1.9]
38. tr|T2GRI9|T2GRI9_MYCHT	K02500 hisF	imidazole glycerol-phosphate synthase subunit HisF [EC:4.3.2.10]
39. tr|T2GRK5|T2GRK5_MYCHT	K01693 hisB	imidazoleglycerol-phosphate dehydratase [EC:4.2.1.19]
40. tr|T2GRL0|T2GRL0_MYCHT	K01496 hisI	phosphoribosyl-AMP cyclohydrolase [EC:3.5.4.19]
41. tr|T2GRL5|T2GRL5_MYCHT	K01609 trpC	indole-3-glycerol phosphate synthase [EC:4.1.1.48]
42. tr|T2GRS3|T2GRS3_MYCHT	K00145 argC	N-acetyl-gamma-glutamyl-phosphate reductase [EC:1.2.1.38]
43. tr|T2GS24|T2GS24_MYCHT	K01714 dapA	4-hydroxy-tetrahydrodipicolinate synthase [EC:4.3.3.7]
44. tr|T2GS72|T2GS72_MYCHT	K03786 aroQ, qutE	3-dehydroquinate dehydratase II [EC:4.2.1.10]
45. tr|T2GSH8|T2GSH8_MYCHT	K21672 ord	2,4-diaminopentanoate dehydrogenase [EC:1.4.1.12 1.4.1.26]
46. tr|T2GSH8|T2GSH8_MYCHT	K21672 ord	2,4-diaminopentanoate dehydrogenase [EC:1.4.1.12 1.4.1.26]
47. tr|T2GSI7|T2GSI7_MYCHT	K00014 aroE	shikimate dehydrogenase [EC:1.1.1.25]
48. tr|T2GSQ1|T2GSQ1_MYCHT	K03782 katG	catalase-peroxidase [EC:1.11.1.21]
49. tr|T2GSQ1|T2GSQ1_MYCHT	K03782 katG	catalase-peroxidase [EC:1.11.1.21]
50. tr|T2GSQ7|T2GSQ7_MYCHT	K01626 E2.5.1.54, aroF, aroG, aroH	3-deoxy-7-phosphoheptulonate synthase [EC:2.5.1.54]
51. tr|T2GSV1|T2GSV1_MYCHT	K01817 trpF	phosphoribosylanthranilate isomerase [EC:5.3.1.24]
52. tr|T2GSV2|T2GSV2_MYCHT	K00249 ACADM, acd	acyl-CoA dehydrogenase [EC:1.3.8.7]
53. tr|T2GSW2|T2GSW2_MYCHT	K01695 trpA	tryptophan synthase alpha chain [EC:4.2.1.20]
54. tr|T2GSW2|T2GSW2_MYCHT	K01695 trpA	tryptophan synthase alpha chain [EC:4.2.1.20]
55. tr|T2GT23|T2GT23_MYCHT	K00930 argB	acetylglutamate kinase [EC:2.7.2.8]
56. tr|T2GT43|T2GT43_MYCHT	K14155 patB, malY	cysteine-S-conjugate beta-lyase [EC:4.4.1.13]
57. tr|T2GT52|T2GT52_MYCHT	K00013 hisD	histidinol dehydrogenase [EC:1.1.1.23]
58. tr|T2GT58|T2GT58_MYCHT	K21672 ord	2,4-diaminopentanoate dehydrogenase [EC:1.4.1.12 1.4.1.26]
59. tr|T2GT58|T2GT58_MYCHT	K21672 ord	2,4-diaminopentanoate dehydrogenase [EC:1.4.1.12 1.4.1.26]
60. tr|T2GT61|T2GT61_MYCHT	K01657 trpE	anthranilate synthase component I [EC:4.1.3.27]
61. tr|T2GT96|T2GT96_MYCHT	K01523 hisE	phosphoribosyl-ATP pyrophosphohydrolase [EC:3.6.1.31]
62. tr|T2GTC2|T2GTC2_MYCHT	K00821 argD	acetylornithine/N-succinyldiaminopimelate aminotransferase [EC:2.6.1.11 2.6.1.17]
63. tr|T2GTC2|T2GTC2_MYCHT	K00821 argD	acetylornithine/N-succinyldiaminopimelate aminotransferase [EC:2.6.1.11 2.6.1.17]
64. tr|T2GTG9|T2GTG9_MYCHT	K01735 aroB	3-dehydroquinate synthase [EC:4.2.3.4]
65. tr|T2GTR5|T2GTR5_MYCHT	K00817 hisC	histidinol-phosphate aminotransferase [EC:2.6.1.9]
66. tr|T2GTR5|T2GTR5_MYCHT	K00817 hisC	histidinol-phosphate aminotransferase [EC:2.6.1.9]
67. tr|T2GTR5|T2GTR5_MYCHT	K00817 hisC	histidinol-phosphate aminotransferase [EC:2.6.1.9]
68. tr|T2GTR5|T2GTR5_MYCHT	K00817 hisC	histidinol-phosphate aminotransferase [EC:2.6.1.9]
69. tr|T2GU13|T2GU13_MYCHT	K01754 E4.3.1.19, ilvA, tdcB	threonine dehydratase [EC:4.3.1.19]
70. tr|T2GU13|T2GU13_MYCHT	K01754 E4.3.1.19, ilvA, tdcB	threonine dehydratase [EC:4.3.1.19]
71. tr|T2GU47|T2GU47_MYCHT	K02501 hisH	imidazole glycerol-phosphate synthase subunit HisH [EC:4.3.2.10]
72. tr|T2GU57|T2GU57_MYCHT	K01696 trpB	tryptophan synthase beta chain [EC:4.2.1.20]
73. tr|T2GU57|T2GU57_MYCHT	K01696 trpB	tryptophan synthase beta chain [EC:4.2.1.20]
74. tr|T2GUB4|T2GUB4_MYCHT	K00215 dapB	4-hydroxy-tetrahydrodipicolinate reductase [EC:1.17.1.8]
75. tr|T2GUC3|T2GUC3_MYCHT	K00620 argJ	glutamate N-acetyltransferase / amino-acid N-acetyltransferase [EC:2.3.1.35 2.3.1.1]
76. tr|T2GUU2|T2GUU2_MYCHT	K01929 murF	UDP-N-acetylmuramoyl-tripeptide--D-alanyl-D-alanine ligase [EC:6.3.2.10]
77. tr|T2GUV4|T2GUV4_MYCHT	K01626 E2.5.1.54, aroF, aroG, aroH	3-deoxy-7-phosphoheptulonate synthase [EC:2.5.1.54]
78. tr|T2GUW9|T2GUW9_MYCHT	K00765 hisG	ATP phosphoribosyltransferase [EC:2.4.2.17]
79. tr|T2GV58|T2GV58_MYCHT	K01928 murE	UDP-N-acetylmuramoyl-L-alanyl-D-glutamate--2,6-diaminopimelate ligase [EC:6.3.2.13]
80. tr|T2GV99|T2GV99_MYCHT	K00865 glxK, garK	glycerate 2-kinase [EC:2.7.1.165]
81. tr|T2GVH4|T2GVH4_MYCHT	K00121 frmA, ADH5, adhC	S-(hydroxymethyl)glutathione dehydrogenase / alcohol dehydrogenase [EC:1.1.1.284 1.1.1.1]
82. tr|T2GVS6|T2GVS6_MYCHT	K01778 dapF	diaminopimelate epimerase [EC:5.1.1.7]
83. tr|T2GVU6|T2GVU6_MYCHT	K00619 argA	amino-acid N-acetyltransferase [EC:2.3.1.1]
84. tr|T2GVX8|T2GVX8_MYCHT	K00215 dapB	4-hydroxy-tetrahydrodipicolinate reductase [EC:1.17.1.8]
85. tr|T2GW70|T2GW70_MYCHT	K00053 ilvC	ketol-acid reductoisomerase [EC:1.1.1.86]
86. tr|T2GWC2|T2GWC2_MYCHT	K21672 ord	2,4-diaminopentanoate dehydrogenase [EC:1.4.1.12 1.4.1.26]
87. tr|T2GWC2|T2GWC2_MYCHT	K21672 ord	2,4-diaminopentanoate dehydrogenase [EC:1.4.1.12 1.4.1.26]
88. tr|T2GWE7|T2GWE7_MYCHT	K00817 hisC	histidinol-phosphate aminotransferase [EC:2.6.1.9]
89. tr|T2GWE7|T2GWE7_MYCHT	K00817 hisC	histidinol-phosphate aminotransferase [EC:2.6.1.9]
90. tr|T2GWE7|T2GWE7_MYCHT	K00817 hisC	histidinol-phosphate aminotransferase [EC:2.6.1.9]
91. tr|T2GWE7|T2GWE7_MYCHT	K00817 hisC	histidinol-phosphate aminotransferase [EC:2.6.1.9]
92. tr|T2GWJ1|T2GWJ1_MYCHT	K01653 E2.2.1.6S, ilvH, ilvN	acetolactate synthase I/III small subunit [EC:2.2.1.6]
93. tr|T2GX92|T2GX92_MYCHT	K00074 paaH, hbd, fadB, mmgB	3-hydroxybutyryl-CoA dehydrogenase [EC:1.1.1.157]
94. tr|T2GXF3|T2GXF3_MYCHT	K01704 leuD, IPMI-S	3-isopropylmalate/(R)-2-methylmalate dehydratase small subunit [EC:4.2.1.33 4.2.1.35]
95. tr|T2GXW6|T2GXW6_MYCHT	K01470 E3.5.2.10	creatinine amidohydrolase [EC:3.5.2.10]
96. tr|T2GY87|T2GY87_MYCHT	K21672 ord	2,4-diaminopentanoate dehydrogenase [EC:1.4.1.12 1.4.1.26]
97. tr|T2GY87|T2GY87_MYCHT	K21672 ord	2,4-diaminopentanoate dehydrogenase [EC:1.4.1.12 1.4.1.26]
98. tr|T2GYA6|T2GYA6_MYCHT	K00800 aroA	3-phosphoshikimate 1-carboxyvinyltransferase [EC:2.5.1.19]
99. tr|T2GYQ1|T2GYQ1_MYCHT	K00641 metX	homoserine O-acetyltransferase/O-succinyltransferase [EC:2.3.1.31 2.3.1.46]
100. tr|T2GYU8|T2GYU8_MYCHT	K17103 CHO1, pssA	CDP-diacylglycerol---serine O-phosphatidyltransferase [EC:2.7.8.8]
101. tr|T2GZ49|T2GZ49_MYCHT	K00215 dapB	4-hydroxy-tetrahydrodipicolinate reductase [EC:1.17.1.8]
102. tr|T2GZH9|T2GZH9_MYCHT	K00249 ACADM, acd	acyl-CoA dehydrogenase [EC:1.3.8.7]
103. tr|T2GZQ5|T2GZQ5_MYCHT	K01687 ilvD	dihydroxy-acid dehydratase [EC:4.2.1.9]

6. Metabolism of Other Amino Acids (beta-Alanine metabolism, Taurine and hypotaurine metabolism, Phosphonate and phosphinate metabolism, Selenocompound metabolism, Glutamine and D-glutamate metabolism, Arginine and D-ornithine metabolism, D-Alanine metabolism)

1. tr|T2GLX0|T2GLX0_MYCHT	K21672 ord	2,4-diaminopentanoate dehydrogenase [EC:1.4.1.12 1.4.1.26]
2. tr|T2GM57|T2GM57_MYCHT	K01919 gshA	glutamate--cysteine ligase [EC:6.3.2.2]
3. tr|T2GMB5|T2GMB5_MYCHT	K01918 panC	pantoate--beta-alanine ligase [EC:6.3.2.1]
4. tr|T2GNL2|T2GNL2_MYCHT	K00549 metE	5-methyltetrahydropteroyltriglutamate--homocysteine methyltransferase [EC:2.1.1.14]
5. tr|T2GP50|T2GP50_MYCHT	K00957 cysD	sulfate adenylyltransferase subunit 2 [EC:2.7.7.4]
6. tr|T2GPA0|T2GPA0_MYCHT	K01776 murI	glutamate racemase [EC:5.1.1.3]
7. tr|T2GPA8|T2GPA8_MYCHT	K21672 ord	2,4-diaminopentanoate dehydrogenase [EC:1.4.1.12 .4.1.26]
8. tr|T2GPG8|T2GPG8_MYCHT	K01579 panD	aspartate 1-decarboxylase [EC:4.1.1.11]
9. tr|T2GRD6|T2GRD6_MYCHT	K15371 GDH2	glutamate dehydrogenase [EC:1.4.1.2]
10. tr|T2GSH8|T2GSH8_MYCHT	K21672 ord	2,4-diaminopentanoate dehydrogenase [EC:1.4.1.12 1.4.1.26]
11. tr|T2GSV2|T2GSV2_MYCHT	K00249 ACADM, acd	acyl-CoA dehydrogenase [EC:1.3.8.7]
12. tr|T2GT43|T2GT43_MYCHT	K14155 patB, malY	cysteine-S-conjugate beta-lyase [EC:4.4.1.13]
13. tr|T2GT58|T2GT58_MYCHT	K21672 ord	2,4-diaminopentanoate dehydrogenase [EC:1.4.1.12 1.4.1.26]
14. tr|T2GTI8|T2GTI8_MYCHT	K01925 murD	UDP-N-acetylmuramoylalanine--D-glutamate ligase [EC:6.3.2.9]
15. tr|T2GUT6|T2GUT6_MYCHT	K01924 murC	UDP-N-acetylmuramate--alanine ligase [EC:6.3.2.8]
16. tr|T2GW31|T2GW31_MYCHT	K00031 IDH1, IDH2, icd	isocitrate dehydrogenase [EC:1.1.1.42]
17. tr|T2GWC2|T2GWC2_MYCHT	K21672 ord	2,4-diaminopentanoate dehydrogenase [EC:1.4.1.12 1.4.1.26]
18. tr|T2GXD9|T2GXD9_MYCHT	K13788 pta	phosphate acetyltransferase [EC:2.3.1.8]
19. tr|T2GY87|T2GY87_MYCHT	K21672 ord	2,4-diaminopentanoate dehydrogenase [EC:1.4.1.12 1.4.1.26]
20. tr|T2GYU6|T2GYU6_MYCHT	K01775 alr	alanine racemase [EC:5.1.1.1]
21. tr|T2GYX6|T2GYX6_MYCHT	K00925 ackA	acetate kinase [EC:2.7.2.1]
22. tr|T2GZH9|T2GZH9_MYCHT	K00249 ACADM, acd	acyl-CoA dehydrogenase [EC:1.3.8.7]
23. tr|T2GZQ6|T2GZQ6_MYCHT	K05349 bglX	beta-glucosidase [EC:3.2.1.21]

8. Glycan Biosynthesis and Metabolism (Lipopolysaccharide biosynthesis, Peptidoglycan biosynthesis, Lipoarabinomannan (LAM) biosynthesis, Arabinogalactan biosynthesis)

1. tr|T2GLU3|T2GLU3_MYCHT	K11387 embC	arabinosyltransferase C [EC:2.4.2.-]
2. tr|T2GLY2|T2GLY2_MYCHT	K16650 glft2	galactofuranosylgalactofuranosylrhamnosyl-N-acetylglucosaminyl-diphospho-decaprenol beta-1,5/1,6-galactofuranosyltransferase [EC:2.4.1.288]
3. tr|T2GLZ0|T2GLZ0_MYCHT	K11385 embA	arabinosyltransferase A [EC:2.4.2.-]
4. tr|T2GLZ5|T2GLZ5_MYCHT	K13686 aftA	galactan 5-O-arabinofuranosyltransferase [EC:2.4.2.46]
5. tr|T2GM16|T2GM16_MYCHT	K07259 dacB	serine-type D-Ala-D-Ala carboxypeptidase/endopeptidase (penicillin-binding protein 4) [EC:3.4.16.4 3.4.21.-]
6. tr|T2GMM3|T2GMM3_MYCHT	K16649 glft1	rhamnopyranosyl-N-acetylglucosaminyl-diphospho-decaprenol beta-1,3/1,4-galactofuranosyltransferase [EC:2.4.1.287]
7. tr|T2GMU5|T2GMU5_MYCHT	K13687 aftB	arabinofuranosyltransferase [EC:2.4.2.-]
8. tr|T2GMU5|T2GMU5_MYCHT	K13687 aftB	arabinofuranosyltransferase [EC:2.4.2.-]
9. tr|T2GN63|T2GN63_MYCHT	K05364 pbpA	penicillin-binding protein A
10. tr|T2GPJ3|T2GPJ3_MYCHT	K03273 gmhB	D-glycero-D-manno-heptose 1,7-bisphosphate phosphatase [EC:3.1.3.82 3.1.3.83]
11. tr|T2GQL9|T2GQL9_MYCHT	K00790 murA	UDP-N-acetylglucosamine 1-carboxyvinyltransferase [EC:2.5.1.7]
12. tr|T2GQN0|T2GQN0_MYCHT	K22311 ptfP1	phosphatidylinositol dimannoside acyltransferase [EC:2.3.1.265]
13. tr|T2GQV4|T2GQV4_MYCHT	K03271 gmhA, lpcA	D-sedoheptulose 7-phosphate isomerase [EC:5.3.1.28]
14. tr|T2GRE7|T2GRE7_MYCHT	K13669 pimE	alpha-1,2-mannosyltransferase [EC:2.4.1.-]
15. tr|T2GRQ0|T2GRQ0_MYCHT	K14340 K14340	mannosyltransferase [EC:2.4.1.-]
16. tr|T2GSG7|T2GSG7_MYCHT	K14339 mptB	alpha-1,6-mannosyltransferase [EC:2.4.1.-]
17. tr|T2GTH6|T2GTH6_MYCHT	K03587 ftsI	cell division protein FtsI (penicillin-binding protein 3) [EC:3.4.16.4]
18. tr|T2GTI8|T2GTI8_MYCHT	K01925 murD	UDP-N-acetylmuramoylalanine--D-glutamate ligase [EC:6.3.2.9]
19. tr|T2GTK5|T2GTK5_MYCHT	K13671 K13671	alpha-1,2-mannosyltransferase [EC:2.4.1.-]
20. tr|T2GTR2|T2GTR2_MYCHT	K06153 bacA	undecaprenyl-diphosphatase [EC:3.6.1.27]
21. tr|T2GUT6|T2GUT6_MYCHT	K01924 murC	UDP-N-acetylmuramate--alanine ligase [EC:6.3.2.8]
22. tr|T2GUU2|T2GUU2_MYCHT	K01929 murF	UDP-N-acetylmuramoyl-tripeptide--D-alanyl-D-alanine ligase [EC:6.3.2.10]
23. tr|T2GV46|T2GV46_MYCHT	K18851 fbp	diacylglycerol O-acyltransferase / trehalose O-mycolyltransferase [EC:2.3.1.20 2.3.1.122]
24. tr|T2GV53|T2GV53_MYCHT	K02563 murG	UDP-N-acetylglucosamine--N-acetylmuramyl-(pentapeptide) pyrophosphoryl-undecaprenol N-acetylglucosamine transferase [EC:2.4.1.227]
25. tr|T2GV58|T2GV58_MYCHT	K01928 murE	UDP-N-acetylmuramoyl-L-alanyl-D-glutamate--2,6-diaminopimelate ligase [EC:6.3.2.13]
26. tr|T2GV68|T2GV68_MYCHT	K14337 mptA	alpha-1,6-mannosyltransferase [EC:2.4.1.-]
27. tr|T2GVB2|T2GVB2_MYCHT	K16647 aftC	arabinofuranan 3-O-arabinosyltransferase [EC:2.4.2.47]
28. tr|T2GVB2|T2GVB2_MYCHT	K16647 aftC	arabinofuranan 3-O-arabinosyltransferase [EC:2.4.2.47]
29. tr|T2GVW9|T2GVW9_MYCHT	K07258 dacC, dacA, dacD	serine-type D-Ala-D-Ala carboxypeptidase (penicillin-binding protein 5/6) [EC:3.4.16.4]
30. tr|T2GW34|T2GW34_MYCHT	K01000 mraY	phospho-N-acetylmuramoyl-pentapeptide-transferase [EC:2.7.8.13]
31. tr|T2GX73|T2GX73_MYCHT	K16870 wbbL	N-acetylglucosaminyl-diphospho-decaprenol L-rhamnosyltransferase [EC:2.4.1.289]
32. tr|T2GY09|T2GY09_MYCHT	K03271 gmhA, lpcA	D-sedoheptulose 7-phosphate isomerase [EC:5.3.1.28]
33. tr|T2GYE1|T2GYE1_MYCHT	K18851 fbp	diacylglycerol O-acyltransferase / trehalose O-mycolyltransferase [EC:2.3.1.20 2.3.1.122]
34. tr|T2GYH3|T2GYH3_MYCHT	K00075 murB	UDP-N-acetylmuramate dehydrogenase [EC:1.3.1.98]
35. tr|T2GYN8|T2GYN8_MYCHT	K07258 dacC, dacA, dacD	serine-type D-Ala-D-Ala carboxypeptidase (penicillin-binding protein 5/6) [EC:3.4.16.4]
36. tr|T2GZ81|T2GZ81_MYCHT	K23176 mtxT	methylthioxylose transferase
37. tr|T2GZP2|T2GZP2_MYCHT	K16648 aftD	arabinofuranan 3-O-arabinosyltransferase [EC:2.4.2.-]
38. tr|T2GZP2|T2GZP2_MYCHT	K16648 aftD	arabinofuranan 3-O-arabinosyltransferase [EC:2.4.2.-]

9. Metabolism of Cofactors and Vitamins (Thiamine metabolism, Riboflavin metabolism, Vitamin B6 metabolism, Nicotinate and Nicotinamide metabolism Pantothenate and CoA biosynthesis, Biotin metabolism Folate biosynthesis, Porphyrin and chlorophyll metabolism Ubiquinone and other Terpenoid-quinone biosynthesis)

1. tr|T2GM26|T2GM26_MYCHT	K01633 folB	7,8-dihydroneopterin aldolase/epimerase/oxygenase [EC:4.1.2.25 5.1.99.8 1.13.11.81]
2. tr|T2GM93|T2GM93_MYCHT	K00059 fabG, OAR1	3-oxoacyl-[acyl-carrier protein] reductase [EC:1.1.1.100]
3. tr|T2GMB2|T2GMB2_MYCHT	K00950 folK	2-amino-4-hydroxy-6-hydroxymethyldihydropteridine diphosphokinase [EC:2.7.6.3]
4. tr|T2GMB5|T2GMB5_MYCHT	K01918 panC	pantoate--beta-alanine ligase [EC:6.3.2.1]
5. tr|T2GN42|T2GN42_MYCHT	K03525 coaX	type III pantothenate kinase [EC:2.7.1.33]
6. tr|T2GNG0|T2GNG0_MYCHT	K01665 pabB	para-aminobenzoate synthetase component I [EC:2.6.1.85]
7. tr|T2GNH0|T2GNH0_MYCHT	K00796 folP	dihydropteroate synthase [EC:2.5.1.15]
8. tr|T2GNN6|T2GNN6_MYCHT	K02619 pabC	4-amino-4-deoxychorismate lyase [EC:4.1.3.38]
9. tr|T2GNV2|T2GNV2_MYCHT	K00796 folP	dihydropteroate synthase [EC:2.5.1.15]
10. tr|T2GPG8|T2GPG8_MYCHT	K01579 panD	aspartate 1-decarboxylase [EC:4.1.1.11]
11. tr|T2GPT8|T2GPT8_MYCHT	K00969 nadD	nicotinate-nucleotide adenylyltransferase [EC:2.7.7.18]
12. tr|T2GQN4|T2GQN4_MYCHT	K08681 pdxT, pdx2	5'-phosphate synthase pdxT subunit [EC:4.3.3.6]
13. tr|T2GQZ6|T2GQZ6_MYCHT	K11752 ribD	diaminohydroxyphosphoribosylaminopyrimidine deaminase / 5-amino-6-(5-phosphoribosylamino)uracil reductase [EC:3.5.4.26 1.1.1.193]
14. tr|T2GQZ9|T2GQZ9_MYCHT	K00794 ribH, RIB4	6,7-dimethyl-8-ribityllumazine synthase [EC:2.5.1.78]
15. tr|T2GR42|T2GR42_MYCHT	K02259 COX15, ctaA	cytochrome c oxidase assembly protein subunit 15
16. tr|T2GRH4|T2GRH4_MYCHT	K01012 bioB	biotin synthase [EC:2.8.1.6]
17. tr|T2GRH9|T2GRH9_MYCHT	K03517 nadA	quinolinate synthase [EC:2.5.1.72]
18. tr|T2GS08|T2GS08_MYCHT	K06215 pdxS, pdx1	pyridoxal 5'-phosphate synthase pdxS subunit [EC:4.3.3.6]
19. tr|T2GS78|T2GS78_MYCHT	K00997 acpS	holo-[acyl-carrier protein] synthase [EC:2.7.8.7]
20. tr|T2GSB9|T2GSB9_MYCHT	K00793 ribE, RIB5	riboflavin synthase [EC:2.5.1.9]
21. tr|T2GSP4|T2GSP4_MYCHT	K14652 ribBA	3,4-dihydroxy 2-butanone 4-phosphate synthase / GTP cyclohydrolase II [EC:4.1.99.12 3.5.4.25]
22. tr|T2GSP4|T2GSP4_MYCHT	K14652 ribBA	3,4-dihydroxy 2-butanone 4-phosphate synthase / GTP cyclohydrolase II [EC:4.1.99.12 3.5.4.25]
23. tr|T2GT40|T2GT40_MYCHT	K01935 bioD	dethiobiotin synthetase [EC:6.3.3.3]
24. tr|T2GT60|T2GT60_MYCHT	K02230 cobN	cobaltochelatase CobN [EC:6.6.1.2]
25. tr|T2GT69|T2GT69_MYCHT	K05895 cobK-cbiJ	precorrin-6A/cobalt-precorrin-6A reductase [EC:1.3.1.54 1.3.1.106]
26. tr|T2GT81|T2GT81_MYCHT	K06042 cobH-cbiC	precorrin-8X/cobalt-precorrin-8 methylmutase [EC:5.4.99.61 5.4.99.60]
27. tr|T2GT86|T2GT86_MYCHT	K05936 cobM, cbiF	precorrin-4/cobalt-precorrin-4 C11-methyltransferase [EC:2.1.1.133 2.1.1.271]
28. tr|T2GTN1|T2GTN1_MYCHT	K02231 cobP, cobU	adenosylcobinamide kinase / adenosylcobinamide-phosphate guanylyltransferase [EC:2.7.1.156 2.7.7.62]
29. tr|T2GTQ3|T2GTQ3_MYCHT	K02227 cbiB, cobD	adenosylcobinamide-phosphate synthase [EC:6.3.1.10]
30. tr|T2GTY9|T2GTY9_MYCHT	K00082 ribD2	5-amino-6-(5-phosphoribosylamino)uracil reductase [EC:1.1.1.193]
31. tr|T2GU50|T2GU50_MYCHT	K00322 sthA, udhA	NAD(P) transhydrogenase [EC:1.6.1.1]
32. tr|T2GUD6|T2GUD6_MYCHT	K02302 cysG	uroporphyrin-III C-methyltransferase / precorrin-2 dehydrogenase / sirohydrochlorin ferrochelatase [EC:2.1.1.107 1.3.1.76 4.99.1.4]
33. tr|T2GUF4|T2GUF4_MYCHT	K02224 cobB-cbiA	cobyrinic acid a,c-diamide synthase [EC:6.3.5.9 6.3.5.11]
34. tr|T2GUF5|T2GUF5_MYCHT	K03151 thiI	tRNA uracil 4-sulfurtransferase [EC:2.8.1.4]
35. tr|T2GUR3|T2GUR3_MYCHT	K00954 E2.7.7.3A, coaD, kdtB	pantetheine-phosphate adenylyltransferase [EC:2.7.7.3]
36. tr|T2GUY4|T2GUY4_MYCHT	K02233 E2.7.8.26, cobS, cobV	adenosylcobinamide-GDP ribazoletransferase [EC:2.7.8.26]
37. tr|T2GUZ4|T2GUZ4_MYCHT	K03644 lipA	lipoyl synthase [EC:2.8.1.8]
38. tr|T2GVC3|T2GVC3_MYCHT	K00606 panB	3-methyl-2-oxobutanoate hydroxymethyltranserase [EC:2.1.2.11]
39. tr|T2GVH4|T2GVH4_MYCHT	K00121 frmA, ADH5, adhC	S-(hydroxymethyl)glutathione dehydrogenase / alcohol dehydrogenase [EC:1.1.1.284 1.1.1.1]
40. tr|T2GVQ9|T2GVQ9_MYCHT	K03404 chlD, bchD	magnesium chelatase subunit D [EC:6.6.1.1]
41. tr|T2GVT3|T2GVT3_MYCHT	K13540 cobIJ	precorrin-2 C20-methyltransferase / precorrin-3B C17-methyltransferase [EC:2.1.1.130 2.1.1.131]
42. tr|T2GVT6|T2GVT6_MYCHT	K00595 cobL	precorrin-6Y C5,15-methyltransferase (decarboxylating) [EC:2.1.1.132]
43. tr|T2GW70|T2GW70_MYCHT	K00053 ilvC	ketol-acid reductoisomerase [EC:1.1.1.86]
44. tr|T2GWA9|T2GWA9_MYCHT	K01239 iunH	purine nucleosidase [EC:3.2.2.1]
45. tr|T2GWH0|T2GWH0_MYCHT	K00946 thiL	thiamine-monophosphate kinase [EC:2.7.4.16]
46. tr|T2GWJ1|T2GWJ1_MYCHT	K01653 E2.2.1.6S, ilvH, ilvN	acetolactate synthase I/III small subunit [EC:2.2.1.6]
47. tr|T2GWR2|T2GWR2_MYCHT	K03465 thyX, thy1	thymidylate synthase (FAD) [EC:2.1.1.148]
48. tr|T2GWY0|T2GWY0_MYCHT	K19221 cobA, btuR	cob(I)alamin adenosyltransferase [EC:2.5.1.17]
49. tr|T2GWY1|T2GWY1_MYCHT	K01661 menB	naphthoate synthase [EC:4.1.3.36]
50. tr|T2GWZ8|T2GWZ8_MYCHT	K02232 cobQ, cbiP	adenosylcobyric acid synthase [EC:6.3.5.10]
51. tr|T2GX09|T2GX09_MYCHT	K02361 entC	isochorismate synthase [EC:5.4.4.2]
52. tr|T2GXD8|T2GXD8_MYCHT	K03147 thiC	phosphomethylpyrimidine synthase [EC:4.1.99.17]
53. tr|T2GXF5|T2GXF5_MYCHT	K08289 purT	phosphoribosylglycinamide formyltransferase 2 [EC:2.1.2.2]
54. tr|T2GXG3|T2GXG3_MYCHT	K03809 wrbA	NAD(P)H dehydrogenase (quinone) [EC:1.6.5.2]
55. tr|T2GYP3|T2GYP3_MYCHT	K00077 panE, apbA	2-dehydropantoate 2-reductase [EC:1.1.1.169]
56. tr|T2GYQ7|T2GYQ7_MYCHT	K02492 hemA	glutamyl-tRNA reductase [EC:1.2.1.70]
57. tr|T2GYW6|T2GYW6_MYCHT	K03149 thiG	thiazole synthase [EC:2.8.1.10]
58. tr|T2GYX7|T2GYX7_MYCHT	K04719 bluB	5,6-dimethylbenzimidazole synthase [EC:1.13.11.79]
59. tr|T2GZ71|T2GZ71_MYCHT	K02551 menD	2-succinyl-5-enolpyruvyl-6-hydroxy-3-cyclohexene-1-carboxylate synthase [EC:2.2.1.9]
60. tr|T2GZ77|T2GZ77_MYCHT	K01719 hemD, UROS	uroporphyrinogen-III synthase [EC:4.2.1.75]
61. tr|T2GZA4|T2GZA4_MYCHT	K13542 cobA-hemD	uroporphyrinogen III methyltransferase / synthase [EC:2.1.1.107 4.2.1.75]
62. tr|T2GZG8|T2GZG8_MYCHT	K00941 thiD	hydroxymethylpyrimidine/phosphomethylpyrimidine kinase [EC:2.7.1.49 2.7.4.7]
63. tr|T2GZH8|T2GZH8_MYCHT	K00788 thiE	thiamine-phosphate pyrophosphorylase [EC:2.5.1.3]
64. tr|T2GZQ5|T2GZQ5_MYCHT	K01687 ilvD	dihydroxy-acid dehydratase [EC:4.2.1.9]

9. Metabolism of Terpenoids and Polyketides (Terpenoid backbone biosynthesis, Limonene and pinene degradation, Polyketide sugar unit biosynthesis, Biosynthesis of siderophore group nonribosomal peptides)

1. tr|T2GN62|T2GN62_MYCHT	K01770 ispF	2-C-methyl-D-erythritol 2,4-cyclodiphosphate synthase [EC:4.6.1.12]
2. tr|T2GNG6|T2GNG6_MYCHT	K14733 limB	limonene 1,2-monooxygenase [EC:1.14.13.107]
3. tr|T2GNI7|T2GNI7_MYCHT	K03527 ispH, lytB	4-hydroxy-3-methylbut-2-en-1-yl diphosphate reductase [EC:1.17.7.4]
4. tr|T2GQ18|T2GQ18_MYCHT	K00919 ispE	4-diphosphocytidyl-2-C-methyl-D-erythritol kinase [EC:2.7.1.148]
5. tr|T2GQ26|T2GQ26_MYCHT	K04793 mbtG	mycobactin lysine-N-oxygenase
6. tr|T2GQ43|T2GQ43_MYCHT	K04781 mbtI, irp9, ybtS	salicylate synthetase [EC:5.4.4.2 4.2.99.21]
7. tr|T2GRG0|T2GRG0_MYCHT	K04787 mbtA	mycobactin salicyl-AMP ligase [EC:6.3.2.-]
8. tr|T2GRG4|T2GRG4_MYCHT	K04789 mbtE	mycobactin peptide synthetase MbtE
9. tr|T2GRT4|T2GRT4_MYCHT	K14215 E2.5.1.86	trans,polycis-decaprenyl diphosphate synthase [EC:2.5.1.86]
10. tr|T2GSM7|T2GSM7_MYCHT	K14733 limB	limonene 1,2-monooxygenase [EC:1.14.13.107]
11. tr|T2GUY0|T2GUY0_MYCHT	K09879 crtU, cruE	carotenoid phi-ring synthase / carotenoid chi-ring synthase [EC:1.3.99.39 1.3.99.40]
12. tr|T2GW30|T2GW30_MYCHT	K00099 dxr	1-deoxy-D-xylulose-5-phosphate reductoisomerase [EC:1.1.1.267]
13. tr|T2GWG7|T2GWG7_MYCHT	K01790 rfbC, rmlC	dTDP-4-dehydrorhamnose 3,5-epimerase [EC:5.1.3.13]
14. tr|T2GX03|T2GX03_MYCHT	K03526 gcpE, ispG	(E)-4-hydroxy-3-methylbut-2-enyl-diphosphate synthase [EC:1.17.7.1 1.17.7.3]
15. tr|T2GX09|T2GX09_MYCHT	K02361 entC	isochorismate synthase [EC:5.4.4.2]

10. Biosynthesis of Other Secondary Metabolites

1. tr|T2GLS4|T2GLS4_MYCHT	K01858 INO1, ISYNA1	myo-inositol-1-phosphate synthase [EC:5.5.1.4]
2. tr|T2GM93|T2GM93_MYCHT	K00059 fabG, OAR1	3-oxoacyl-[acyl-carrier protein] reductase [EC:1.1.1.100]
3. tr|T2GMU4|T2GMU4_MYCHT	K00928 lysC	aspartate kinase [EC:2.7.2.4]
4. tr|T2GN13|T2GN13_MYCHT	K04517 tyrA2	prephenate dehydrogenase [EC:1.3.1.12]
5. tr|T2GN72|T2GN72_MYCHT	K00133 asd	aspartate-semialdehyde dehydrogenase [EC:1.2.1.11]
6. tr|T2GP50|T2GP50_MYCHT	K00957 cysD	sulfate adenylyltransferase subunit 2 [EC:2.7.7.4]
7. tr|T2GRI4|T2GRI4_MYCHT	K00817 hisC	histidinol-phosphate aminotransferase [EC:2.6.1.9]
8. tr|T2GRI4|T2GRI4_MYCHT	K00817 hisC	histidinol-phosphate aminotransferase [EC:2.6.1.9]
9. tr|T2GS24|T2GS24_MYCHT	K01714 dapA	4-hydroxy-tetrahydrodipicolinate synthase [EC:4.3.3.7]
10. tr|T2GSQ1|T2GSQ1_MYCHT	K03782 katG	catalase-peroxidase [EC:1.11.1.21]
11. tr|T2GT61|T2GT61_MYCHT	K01657 trpE	anthranilate synthase component I [EC:4.1.3.27]
12. tr|T2GTP1|T2GTP1_MYCHT	K02078 acpP	acyl carrier protein
13. tr|T2GTR5|T2GTR5_MYCHT	K00817 hisC	histidinol-phosphate aminotransferase [EC:2.6.1.9]
14. tr|T2GTR5|T2GTR5_MYCHT	K00817 hisC	histidinol-phosphate aminotransferase [EC:2.6.1.9]
15. tr|T2GUB4|T2GUB4_MYCHT	K00215 dapB	4-hydroxy-tetrahydrodipicolinate reductase [EC:1.17.1.8]
16. tr|T2GVX8|T2GVX8_MYCHT	K00215 dapB	4-hydroxy-tetrahydrodipicolinate reductase [EC:1.17.1.8]
17. tr|T2GWE7|T2GWE7_MYCHT	K00817 hisC	histidinol-phosphate aminotransferase [EC:2.6.1.9]
18. tr|T2GWE7|T2GWE7_MYCHT	K00817 hisC	histidinol-phosphate aminotransferase [EC:2.6.1.9]
19. tr|T2GWG7|T2GWG7_MYCHT	K01790 rfbC, rmlC	dTDP-4-dehydrorhamnose 3,5-epimerase [EC:5.1.3.13]
20. tr|T2GZ49|T2GZ49_MYCHT	K00215 dapB	4-hydroxy-tetrahydrodipicolinate reductase [EC:1.17.1.8]
21. tr|T2GZQ6|T2GZQ6_MYCHT	K05349 bglX	beta-glucosidase [EC:3.2.1.21]

11. Xenobiotics Biodegradation and Metabolism (Benzoate degradation, Aminobenzoate degradation, Xylene degradation, Ethylbenzene degradation, Atrazine degradation Dioxin degradation)

1. tr|T2GQ31|T2GQ31_MYCHT	K01607 pcaC	4-carboxymuconolactone decarboxylase [EC:4.1.1.44]
2. tr|T2GX92|T2GX92_MYCHT	K00074 paaH, hbd, fadB, mmgB	3-hydroxybutyryl-CoA dehydrogenase [EC:1.1.1.157]
3. tr|T2GVH4|T2GVH4_MYCHT	K00121 frmA, ADH5, adhC	S-(hydroxymethyl)glutathione dehydrogenase / alcohol dehydrogenase [EC:1.1.1.284 1.1.1.1]
4. tr|T2GM01|T2GM01_MYCHT	K06912 tfdA	alpha-ketoglutarate-dependent 2,4-dichlorophenoxyacetate dioxygenase [EC:1.14.11.-]
5. tr|T2GQT9|T2GQT9_MYCHT	K03379 chnB	cyclohexanone monooxygenase [EC:1.14.13.22]
6. tr|T2GZR2|T2GZR2_MYCHT	K03379 chnB	cyclohexanone monooxygenase [EC:1.14.13.22]
7. tr|T2GS28|T2GS28_MYCHT	K03379 chnB	cyclohexanone monooxygenase [EC:1.14.13.22]
8. tr|T2GPJ2|T2GPJ2_MYCHT	K06446 DCAA	acyl-CoA dehydrogenase [EC:1.3.99.-]
9. tr|T2GVH4|T2GVH4_MYCHT	K00121 frmA, ADH5, adhC	S-(hydroxymethyl)glutathione dehydrogenase / alcohol dehydrogenase [EC:1.1.1.284 1.1.1.1]
10. tr|T2GPN4|T2GPN4_MYCHT	K15981 CYP125A	cholest-4-en-3-one 26-monooxygenase [EC:1.14.15.29]
11. tr|T2GUL0|T2GUL0_MYCHT	K15982 kshA	3-ketosteroid 9alpha-monooxygenase subunit A [EC:1.14.15.30]
12. tr|T2GM56|T2GM56_MYCHT	K16048 hsaB	3-hydroxy-9,10-secoandrosta-1,3,5(10)-triene-9,17-dione monooxygenase reductase component [EC:1.5.1.-]
13. tr|T2GNL5|T2GNL5_MYCHT	K16049 hsaC	3,4-dihydroxy-9,10-secoandrosta-1,3,5(10)-triene-9,17-dione 4,5-dioxygenase [EC:1.13.11.25]
14. tr|T2GS25|T2GS25_MYCHT	K16051 tesI	3-oxo-5alpha-steroid 4-dehydrogenase [EC:1.3.99.5]
15. tr|T2GVH4|T2GVH4_MYCHT	K00121 frmA, ADH5, adhC	S-(hydroxymethyl)glutathione dehydrogenase / alcohol dehydrogenase [EC:1.1.1.284 1.1.1.1]
16. tr|T2GVH4|T2GVH4_MYCHT	K00121 frmA, ADH5, adhC	S-(hydroxymethyl)glutathione dehydrogenase / alcohol dehydrogenase [EC:1.1.1.284 1.1.1.1]
17. tr|T2GW91|T2GW91_MYCHT	K00088 IMPDH, guaB	IMP dehydrogenase [EC:1.1.1.205]
18. tr|T2GSQ1|T2GSQ1_MYCHT	K03782 katG	catalase-peroxidase [EC:1.11.1.21]]

Genetic Information Processing:

12. Transcription, Translation, Replication and Repair

1. tr|T2GLL7|T2GLL7_MYCHT	K02338 dnaN	DNA polymerase III subunit beta [EC:2.7.7.7]
2. tr|T2GLL7|T2GLL7_MYCHT	K02338 dnaN	DNA polymerase III subunit beta [EC:2.7.7.7]
3. tr|T2GLL7|T2GLL7_MYCHT	K02338 dnaN	DNA polymerase III subunit beta [EC:2.7.7.7]
4. tr|T2GLP1|T2GLP1_MYCHT	K03629 recF	DNA replication and repair protein RecF
5. tr|T2GM52|T2GM52_MYCHT	K02342 dnaQ	DNA polymerase III subunit epsilon [EC:2.7.7.7]
6. tr|T2GM52|T2GM52_MYCHT	K02342 dnaQ	DNA polymerase III subunit epsilon [EC:2.7.7.7]
7. tr|T2GM52|T2GM52_MYCHT	K02342 dnaQ	DNA polymerase III subunit epsilon [EC:2.7.7.7]
8. tr|T2GM76|T2GM76_MYCHT	K02990 RP-S6, MRPS6, rpsF	small subunit ribosomal protein S6
9. tr|T2GMI5|T2GMI5_MYCHT	K03111 ssb	single-strand DNA-binding protein
10. tr|T2GMI5|T2GMI5_MYCHT	K03111 ssb	single-strand DNA-binding protein
11. tr|T2GMI5|T2GMI5_MYCHT	K03111 ssb	single-strand DNA-binding protein
12. tr|T2GMU1|T2GMU1_MYCHT	K06187 recR	recombination protein RecR
13. tr|T2GND2|T2GND2_MYCHT	K02314 dnaB	replicative DNA helicase [EC:3.6.4.12]
14. tr|T2GNJ6|T2GNJ6_MYCHT	K01883 CARS, cysS	cysteinyl-tRNA synthetase [EC:6.1.1.16]
15. tr|T2GPB5|T2GPB5_MYCHT	K10979 ku	DNA end-binding protein Ku
16. tr|T2GPC9|T2GPC9_MYCHT	K10563 mutM, fpg	formamidopyrimidine-DNA glycosylase [EC:3.2.2.23 4.2.99.18]
17. tr|T2GPV9|T2GPV9_MYCHT	K02340 holA	DNA polymerase III subunit delta [EC:2.7.7.7]
18. tr|T2GPV9|T2GPV9_MYCHT	K02340 holA	DNA polymerase III subunit delta [EC:2.7.7.7]
19. tr|T2GPV9|T2GPV9_MYCHT	K02340 holA	DNA polymerase III subunit delta [EC:2.7.7.7]
20. tr|T2GPX1|T2GPX1_MYCHT	K03601 xseA	exodeoxyribonuclease VII large subunit [EC:3.1.11.6]
21. tr|T2GQ23|T2GQ23_MYCHT	K02968 RP-S20, rpsT	small subunit ribosomal protein S20
22. tr|T2GQ57|T2GQ57_MYCHT	K02316 dnaG	DNA primase [EC:2.7.7.101]
23. tr|T2GQ97|T2GQ97_MYCHT	K01246 tag	DNA-3-methyladenine glycosylase I [EC:3.2.2.20]
24. tr|T2GQP7|T2GQP7_MYCHT	K03074 secF	preprotein translocase subunit SecF
25. tr|T2GQP8|T2GQP8_MYCHT	K03657 uvrD, pcrA	DNA helicase II / ATP-dependent DNA helicase PcrA [EC:3.6.4.12]
26. tr|T2GQP8|T2GQP8_MYCHT	K03657 uvrD, pcrA	DNA helicase II / ATP-dependent DNA helicase PcrA [EC:3.6.4.12]
27. tr|T2GR01|T2GR01_MYCHT	K02897 RP-L25, rplY	large subunit ribosomal protein L25
28. tr|T2GR04|T2GR04_MYCHT	K03703 uvrC	excinuclease ABC subunit C
29. tr|T2GR78|T2GR78_MYCHT	K03602 xseB	exodeoxyribonuclease VII small subunit [EC:3.1.11.6]
30. tr|T2GRE8|T2GRE8_MYCHT	K02337 dnaE	DNA polymerase III subunit alpha [EC:2.7.7.7]
31. tr|T2GRE8|T2GRE8_MYCHT	K02337 dnaE	DNA polymerase III subunit alpha [EC:2.7.7.7]
32. tr|T2GRE8|T2GRE8_MYCHT	K02337 dnaE	DNA polymerase III subunit alpha [EC:2.7.7.7]
33. tr|T2GRG3|T2GRG3_MYCHT	K02888 RP-L21, MRPL21, rplU	large subunit ribosomal protein L21
34. tr|T2GRI5|T2GRI5_MYCHT	K03584 recO	DNA repair protein RecO (recombination protein O)
35. tr|T2GRS8|T2GRS8_MYCHT	K21929 udg	uracil-DNA glycosylase [EC:3.2.2.27]
36. tr|T2GRW3|T2GRW3_MYCHT	K02909 RP-L31, rpmE	large subunit ribosomal protein L31
37. tr|T2GS17|T2GS17_MYCHT	K03550 ruvA	holliday junction DNA helicase RuvA [EC:3.6.4.12]
38. tr|T2GS22|T2GS22_MYCHT	K03210 yajC	preprotein translocase subunit YajC
39. tr|T2GSA1|T2GSA1_MYCHT	K03060 rpoZ	DNA-directed RNA polymerase subunit omega [EC:2.7.7.6]
40. tr|T2GSN4|T2GSN4_MYCHT	K04066 priA	primosomal protein N' (replication factor Y) (superfamily II helicase) [EC:3.6.4.-]
41. tr|T2GSQ5|T2GSQ5_MYCHT	K03101 lspA	signal peptidase II [EC:3.4.23.36]
42. tr|T2GT11|T2GT11_MYCHT	K02916 RP-L35, MRPL35, rpmI	large subunit ribosomal protein L35
43. tr|T2GT95|T2GT95_MYCHT	K03118 tatC	sec-independent protein translocase protein TatC
44. tr|T2GTA0|T2GTA0_MYCHT	K03432 psmA, prcA	proteasome alpha subunit [EC:3.4.25.1]
45. tr|T2GTC3|T2GTC3_MYCHT	K01159 ruvC	crossover junction endodeoxyribonuclease RuvC [EC:3.1.22.4]
46. tr|T2GTM1|T2GTM1_MYCHT	K03070 secA	preprotein translocase subunit SecA [EC:7.4.2.8]
47. tr|T2GUF5|T2GUF5_MYCHT	K03151 thiI	tRNA uracil 4-sulfurtransferase [EC:2.8.1.4]
48. tr|T2GUW6|T2GUW6_MYCHT	K02342 dnaQ	DNA polymerase III subunit epsilon [EC:2.7.7.7]
49. tr|T2GUW6|T2GUW6_MYCHT	K02342 dnaQ	DNA polymerase III subunit epsilon [EC:2.7.7.7]
50. tr|T2GUW6|T2GUW6_MYCHT	K02342 dnaQ	DNA polymerase III subunit epsilon [EC:2.7.7.7]
51. tr|T2GVR6|T2GVR6_MYCHT	K22468 ppk2	polyphosphate kinase [EC:2.7.4.1]
52. tr|T2GVU7|T2GVU7_MYCHT	K03116 tatA	sec-independent protein translocase protein TatA
53. tr|T2GWH4|T2GWH4_MYCHT	K00937 ppk	polyphosphate kinase [EC:2.7.4.1]
54. tr|T2GWJ0|T2GWJ0_MYCHT	K02904 RP-L29, rpmC	large subunit ribosomal protein L29
55. tr|T2GWK0|T2GWK0_MYCHT	K02435 gatC, GATC	aspartyl-tRNA(Asn)/glutamyl-tRNA(Gln) amidotransferase subunit C [EC:6.3.5.6 6.3.5.7]
56. tr|T2GWL1|T2GWL1_MYCHT	K02881 RP-L18, MRPL18, rplR	large subunit ribosomal protein L18
57. tr|T2GWR1|T2GWR1_MYCHT	K01151 nfo	deoxyribonuclease IV [EC:3.1.21.2]
58. tr|T2GWR9|T2GWR9_MYCHT	K03582 recB	exodeoxyribonuclease V beta subunit [EC:3.1.11.5]
59. tr|T2GWU4|T2GWU4_MYCHT	K21929 udg	uracil-DNA glycosylase [EC:3.2.2.27]
60. tr|T2GXC5|T2GXC5_MYCHT	K05592 deaD, cshA	ATP-dependent RNA helicase DeaD [EC:3.6.4.13]
61. tr|T2GXC6|T2GXC6_MYCHT	K02902 RP-L28, MRPL28, rpmB	large subunit ribosomal protein L28
62. tr|T2GXC8|T2GXC8_MYCHT	K03154 thiS	sulfur carrier protein
63. tr|T2GXP5|T2GXP5_MYCHT	K03040 rpoA	DNA-directed RNA polymerase subunit alpha [EC:2.7.7.6]
64. tr|T2GXT9|T2GXT9_MYCHT	K02902 RP-L28, MRPL28, rpmB	large subunit ribosomal protein L28
65. tr|T2GXV7|T2GXV7_MYCHT	K02890 RP-L22, MRPL22, rplV	large subunit ribosomal protein L22
66. tr|T2GXZ9|T2GXZ9_MYCHT	K02864 RP-L10, MRPL10, rplJ	large subunit ribosomal protein L10
67. tr|T2GY16|T2GY16_MYCHT	K03073 secE	preprotein translocase subunit SecE
68. tr|T2GY29|T2GY29_MYCHT	K02986 RP-S4, rpsD	small subunit ribosomal protein S4
69. tr|T2GY69|T2GY69_MYCHT	K03657 uvrD, pcrA	DNA helicase II / ATP-dependent DNA helicase PcrA [EC:3.6.4.12]
70. tr|T2GY69|T2GY69_MYCHT	K03657 uvrD, pcrA	DNA helicase II / ATP-dependent DNA helicase PcrA [EC:3.6.4.12]
71. tr|T2GY74|T2GY74_MYCHT	K02907 RP-L30, MRPL30, rpmD	large subunit ribosomal protein L30
72. tr|T2GY88|T2GY88_MYCHT	K02961 RP-S17, MRPS17, rpsQ	small subunit ribosomal protein S17
73. tr|T2GYG1|T2GYG1_MYCHT	K03583 recC	exodeoxyribonuclease V gamma subunit [EC:3.1.11.5]
74. tr|T2GYR3|T2GYR3_MYCHT	K03217 yidC, spoIIIJ, OXA1, ccfA	YidC/Oxa1 family membrane protein insertase
75. tr|T2GYZ9|T2GYZ9_MYCHT	K02982 RP-S3, rpsC	small subunit ribosomal protein S3
76. tr|T2GZ32|T2GZ32_MYCHT	K02913 RP-L33, MRPL33, rpmG	large subunit ribosomal protein L33
77. tr|T2GZI0|T2GZI0_MYCHT	K02914 RP-L34, MRPL34, rpmH	large subunit ribosomal protein L34
78. tr|T2GZK5|T2GZK5_MYCHT	K04043 dnaK, HSPA9	molecular chaperone DnaK

Environmental Information Processing:

13. Membrane Transport, Signal Transduction

1. tr|T2GLP2|T2GLP2_MYCHT	K02066 mlaE, linK	phospholipid/cholesterol/gamma-HCH transport system permease protein
2. tr|T2GM02|T2GM02_MYCHT	K15582 oppC	oligopeptide transport system permease protein
3. tr|T2GM07|T2GM07_MYCHT	K02313 dnaA	chromosomal replication initiator protein
4. tr|T2GMI7|T2GMI7_MYCHT	K02066 mlaE, linK	phospholipid/cholesterol/gamma-HCH transport system permease protein
5. tr|T2GMM9|T2GMM9_MYCHT	K15554 ssuC	sulfonate transport system permease protein
6. tr|T2GMX1|T2GMX1_MYCHT	K07655 prrB	two-component system, OmpR family, sensor histidine kinase PrrB [EC:2.7.13.3]
7. tr|T2GMZ0|T2GMZ0_MYCHT	K02040 pstS	phosphate transport system substrate-binding protein
8. tr|T2GMZ0|T2GMZ0_MYCHT	K02040 pstS	phosphate transport system substrate-binding protein
9. tr|T2GMZ2|T2GMZ2_MYCHT	K15580 oppA, mppA	oligopeptide transport system substrate-binding protein
10. tr|T2GMZ5|T2GMZ5_MYCHT	K02066 mlaE, linK	phospholipid/cholesterol/gamma-HCH transport system permease protein
11. tr|T2GN30|T2GN30_MYCHT	K07669 mprA	two-component system, OmpR family, response regulator MprA
12. tr|T2GN64|T2GN64_MYCHT	K07671 prrA	two-component system, OmpR family, response regulator PrrA
13. tr|T2GN87|T2GN87_MYCHT	K02037 pstC	phosphate transport system permease protein
14. tr|T2GNC8|T2GNC8_MYCHT	K07653 mprB	two-component system, OmpR family, sensor histidine kinase MprB [EC:2.7.13.3]
15. tr|T2GND0|T2GND0_MYCHT	K07672 trcR	two-component system, OmpR family, response regulator TrcR
16. tr|T2GND4|T2GND4_MYCHT	K15581 oppB	oligopeptide transport system permease protein
17. tr|T2GNX0|T2GNX0_MYCHT	K00370 narG, narZ, nxrA	nitrate reductase / nitrite oxidoreductase, alpha subunit [EC:1.7.5.1 1.7.99.-]
18. tr|T2GNZ7|T2GNZ7_MYCHT	K05846 opuBD	osmoprotectant transport system permease protein
19. tr|T2GP01|T2GP01_MYCHT	K02038 pstA	phosphate transport system permease protein
20. tr|T2GP49|T2GP49_MYCHT	K07684 narL	two-component system, NarL family, nitrate/nitrite response regulator NarL
21. tr|T2GPQ7|T2GPQ7_MYCHT	K07667 kdpE	two-component system, OmpR family, KDP operon response regulator KdpE
22. tr|T2GPS5|T2GPS5_MYCHT	K02066 mlaE, linK	phospholipid/cholesterol/gamma-HCH transport system permease protein
23. tr|T2GPX3|T2GPX3_MYCHT	K02047 cysW	sulfate/thiosulfate transport system permease protein
24. tr|T2GQ36|T2GQ36_MYCHT	K07646 kdpD	two-component system, OmpR family, sensor histidine kinase KdpD [EC:2.7.13.3]
25. tr|T2GQ41|T2GQ41_MYCHT	K07656 trcS	two-component system, OmpR family, sensor histidine kinase TrcS [EC:2.7.13.3]
26. tr|T2GQ44|T2GQ44_MYCHT	K00373 narJ, narW	nitrate reductase molybdenum cofactor assembly chaperone NarJ/NarW
27. tr|T2GQ71|T2GQ71_MYCHT	K02066 mlaE, linK	phospholipid/cholesterol/gamma-HCH transport system permease protein
28. tr|T2GQC9|T2GQC9_MYCHT	K02066 mlaE, linK	phospholipid/cholesterol/gamma-HCH transport system permease protein
29. tr|T2GQE8|T2GQE8_MYCHT	K02066 mlaE, linK	phospholipid/cholesterol/gamma-HCH transport system permease protein
30. tr|T2GQN1|T2GQN1_MYCHT	K02038 pstA	phosphate transport system permease protein
31. tr|T2GQP7|T2GQP7_MYCHT	K03074 secF	preprotein translocase subunit SecF
32. tr|T2GR21|T2GR21_MYCHT	K01546 kdpA	potassium-transporting ATPase potassium-binding subunit
33. tr|T2GR42|T2GR42_MYCHT	K02259 COX15, ctaA	cytochrome c oxidase assembly protein subunit 15
34. tr|T2GRA7|T2GRA7_MYCHT	K23163 sbp	sulfate/thiosulfate transport system substrate-binding protein
35. tr|T2GRF2|T2GRF2_MYCHT	K00371 narH, narY, nxrB	nitrate reductase / nitrite oxidoreductase, beta subunit [EC:1.7.5.1 1.7.99.-]
36. tr|T2GRL7|T2GRL7_MYCHT	K02046 cysU	sulfate/thiosulfate transport system permease protein
37. tr|T2GRM7|T2GRM7_MYCHT	K00426 cydB	cytochrome bd ubiquinol oxidase subunit II [EC:7.1.1.7]
38. tr|T2GRN4|T2GRN4_MYCHT	K16787 ecfA2	energy-coupling factor transport system ATP-binding protein [EC:3.6.3.-]
39. tr|T2GRX7|T2GRX7_MYCHT	K16785 ecfT	energy-coupling factor transport system permease protein
40. tr|T2GS22|T2GS22_MYCHT	K03210 yajC	preprotein translocase subunit YajC
41. tr|T2GSE9|T2GSE9_MYCHT	K02018 modB	molybdate transport system permease protein
42. tr|T2GT72|T2GT72_MYCHT	K16012 cydC	ATP-binding cassette, subfamily C, bacterial CydC
43. tr|T2GT95|T2GT95_MYCHT	K03118 tatC	sec-independent protein translocase protein TatC
44. tr|T2GTM1|T2GTM1_MYCHT	K03070 secA	preprotein translocase subunit SecA [EC:7.4.2.8]
45. tr|T2GU69|T2GU69_MYCHT	K00425 cydA	cytochrome bd ubiquinol oxidase subunit I [EC:7.1.1.7]
46. tr|T2GUK4|T2GUK4_MYCHT	K00990 glnD	[protein-PII] uridylyltransferase [EC:2.7.7.59]
47. tr|T2GUL7|T2GUL7_MYCHT	K04751 glnB	nitrogen regulatory protein P-II 1
48. tr|T2GUM4|T2GUM4_MYCHT	K02066 mlaE, linK	phospholipid/cholesterol/gamma-HCH transport system permease protein
49. tr|T2GUS7|T2GUS7_MYCHT	K15554 ssuC	sulfonate transport system permease protein
50. tr|T2GVS7|T2GVS7_MYCHT	K15553 ssuA	sulfonate transport system substrate-binding protein
51. tr|T2GVU7|T2GVU7_MYCHT	K03116 tatA	sec-independent protein translocase protein TatA
52. tr|T2GVW7|T2GVW7_MYCHT	K02066 mlaE, linK	phospholipid/cholesterol/gamma-HCH transport system permease protein
53. tr|T2GWS9|T2GWS9_MYCHT	K02066 mlaE, linK	phospholipid/cholesterol/gamma-HCH transport system permease protein
54. tr|T2GWV9|T2GWV9_MYCHT	K07695 devR	two-component system, NarL family, response regulator DevR
55. tr|T2GX41|T2GX41_MYCHT	K03412 cheB	two-component system, chemotaxis family, protein-glutamate methylesterase/glutaminase [EC:3.1.1.61 3.5.1.44]
56. tr|T2GX44|T2GX44_MYCHT	K07776 regX3	two-component system, OmpR family, response regulator RegX3
57. tr|T2GX55|T2GX55_MYCHT	K07670 mtrA	two-component system, OmpR family, response regulator MtrA
58. tr|T2GX72|T2GX72_MYCHT	K07768 senX3	two-component system, OmpR family, sensor histidine kinase SenX3 [EC:2.7.13.3]
59. tr|T2GXS7|T2GXS7_MYCHT	K09811 ftsX	cell division transport system permease protein
60. tr|T2GY16|T2GY16_MYCHT	K03073 secE	preprotein translocase subunit SecE
61. tr|T2GY60|T2GY60_MYCHT	K02066 mlaE, linK	phospholipid/cholesterol/gamma-HCH transport system permease protein
62. tr|T2GYD2|T2GYD2_MYCHT	K07654 mtrB	two-component system, OmpR family, sensor histidine kinase MtrB [EC:2.7.13.3]
63. tr|T2GYG5|T2GYG5_MYCHT	K02040 pstS	phosphate transport system substrate-binding protein
64. tr|T2GYG5|T2GYG5_MYCHT	K02040 pstS	phosphate transport system substrate-binding protein
65. tr|T2GYG9|T2GYG9_MYCHT	K02066 mlaE, linK	phospholipid/cholesterol/gamma-HCH transport system permease protein
66. tr|T2GYR3|T2GYR3_MYCHT	K03217 yidC, spoIIIJ, OXA1, ccfA	YidC/Oxa1 family membrane protein insertase
67. tr|T2GYZ6|T2GYZ6_MYCHT	K02040 pstS	phosphate transport system substrate-binding protein
68. tr|T2GYZ6|T2GYZ6_MYCHT	K02040 pstS	phosphate transport system substrate-binding protein
69. tr|T2GZE1|T2GZE1_MYCHT	K02066 mlaE, linK	phospholipid/cholesterol/gamma-HCH transport system permease protein

Cellular Processes:

14. Transport and Catabolism, Cell Growth and Death

1. tr|T2GM02|T2GM02_MYCHT	K15582 oppC	oligopeptide transport system permease protein
2. tr|T2GM07|T2GM07_MYCHT	K02313 dnaA	chromosomal replication initiator protein
3. tr|T2GMZ2|T2GMZ2_MYCHT	K15580 oppA, mppA	oligopeptide transport system substrate-binding protein
4. tr|T2GND2|T2GND2_MYCHT	K02314 dnaB	replicative DNA helicase [EC:3.6.4.12]
5. tr|T2GND4|T2GND4_MYCHT	K15581 oppB	oligopeptide transport system permease protein
6. tr|T2GPB7|T2GPB7_MYCHT	K02034 ABC.PE.P1	peptide/nickel transport system permease protein
7. tr|T2GPQ7|T2GPQ7_MYCHT	K07667 kdpE	two-component system, OmpR family, KDP operon response regulator KdpE
8. tr|T2GQB9|T2GQB9_MYCHT	K00640 cysE	serine O-acetyltransferase [EC:2.3.1.30]
9. tr|T2GQV1|T2GQV1_MYCHT	K02035 ABC.PE.S	peptide/nickel transport system substrate-binding protein
10. tr|T2GQV2|T2GQV2_MYCHT	K02035 ABC.PE.S	peptide/nickel transport system substrate-binding protein
11. tr|T2GQZ6|T2GQZ6_MYCHT	K11752 ribD	diaminohydroxyphosphoribosylaminopyrimidine deaminase / 5-amino-6-(5-phosphoribosylamino)uracil reductase [EC:3.5.4.26 1.1.1.193]
12. tr|T2GQZ7|T2GQZ7_MYCHT	K02035 ABC.PE.S	peptide/nickel transport system substrate-binding protein
13. tr|T2GRU5|T2GRU5_MYCHT	K02033 ABC.PE.P	peptide/nickel transport system permease protein
14. tr|T2GS22|T2GS22_MYCHT	K03210 yajC	preprotein translocase subunit YajC
15. tr|T2GS66|T2GS66_MYCHT	K02034 ABC.PE.P1	peptide/nickel transport system permease protein
16. tr|T2GSQ7|T2GSQ7_MYCHT	K01626 E2.5.1.54, aroF, aroG, aroH	3-deoxy-7-phosphoheptulonate synthase [EC:2.5.1.54]
17. tr|T2GT61|T2GT61_MYCHT	K01657 trpE	anthranilate synthase component I [EC:4.1.3.27]
18. tr|T2GT61|T2GT61_MYCHT	K01657 trpE	anthranilate synthase component I [EC:4.1.3.27]
19. tr|T2GTG4|T2GTG4_MYCHT	K02033 ABC.PE.P	peptide/nickel transport system permease protein
20. tr|T2GTH0|T2GTH0_MYCHT	K03588 ftsW, spoVE	cell division protein FtsW
21. tr|T2GTI4|T2GTI4_MYCHT	K03531 ftsZ	cell division protein FtsZ
22. tr|T2GTM1|T2GTM1_MYCHT	K03070 secA	preprotein translocase subunit SecA [EC:7.4.2.8]
23. tr|T2GTS8|T2GTS8_MYCHT	K04761 oxyR	LysR family transcriptional regulator, hydrogen peroxide-inducible genes activator
24. tr|T2GUV4|T2GUV4_MYCHT	K01626 E2.5.1.54, aroF, aroG, aroH	3-deoxy-7-phosphoheptulonate synthase [EC:2.5.1.54]
25. tr|T2GV53|T2GV53_MYCHT	K02563 murG	UDP-N-acetylglucosamine--N-acetylmuramyl-(pentapeptide) pyrophosphoryl-undecaprenol N-acetylglucosamine transferase [EC:2.4.1.227]
26. tr|T2GVE8|T2GVE8_MYCHT	K03087 rpoS	RNA polymerase nonessential primary-like sigma factor
27. tr|T2GVE8|T2GVE8_MYCHT	K03087 rpoS	RNA polymerase nonessential primary-like sigma factor
28. tr|T2GW31|T2GW31_MYCHT	K00031 IDH1, IDH2, icd	isocitrate dehydrogenase [EC:1.1.1.42]
29. tr|T2GW33|T2GW33_MYCHT	K03589 ftsQ	cell division protein FtsQ
30. tr|T2GWB1|T2GWB1_MYCHT	K00803 AGPS, agpS	alkyldihydroxyacetonephosphate synthase [EC:2.5.1.26]
31. tr|T2GX41|T2GX41_MYCHT	K03412 cheB	two-component system, chemotaxis family, protein-glutamate methylesterase/glutaminase [EC:3.1.1.61 3.5.1.44]
32. tr|T2GY16|T2GY16_MYCHT	K03073 secE	preprotein translocase subunit SecE
33. tr|T2GYR3|T2GYR3_MYCHT	K03217 yidC, spoIIIJ, OXA1, ccfA	YidC/Oxa1 family membrane protein insertase

Organismal Systems

15. Immune system
1. tr|T2GZV0|T2GZV0_MYCHT	K03671 trxA	thioredoxin 1
16. Endocrine system

1. tr|T2GRM1|T2GRM1_MYCHT	K00873 PK, pyk	pyruvate kinase [EC:2.7.1.40]
2. tr|T2GWN4|T2GWN4_MYCHT	K00255 ACADL	long-chain-acyl-CoA dehydrogenase [EC:1.3.8.8]
3. tr|T2GSV2|T2GSV2_MYCHT	K00249 ACADM, acd	acyl-CoA dehydrogenase [EC:1.3.8.7]
4. tr|T2GZH9|T2GZH9_MYCHT	K00249 ACADM, acd	acyl-CoA dehydrogenase [EC:1.3.8.7]
17. Aging

1. tr|T2GZK5|T2GZK5_MYCHT	K04043 dnaK, HSPA9	molecular chaperone DnaK
18. Environmental adaptation

1. tr|T2GR42|T2GR42_MYCHT	K02259 COX15, ctaA	cytochrome c oxidase assembly protein subunit 15

Human Diseases

19. Cancers, Cardiovascular diseases, Drug resistance: Antimicrobial (beta-Lactam resistance and Vancomycin resistance)

1. tr|T2GLX6|T2GLX6_MYCHT	K14952 namH	UDP-MurNAc hydroxylase
2. tr|T2GM02|T2GM02_MYCHT	K15582 oppC	oligopeptide transport system permease protein
3. tr|T2GMZ0|T2GMZ0_MYCHT	K02040 pstS	phosphate transport system substrate-binding protein
4. tr|T2GMZ2|T2GMZ2_MYCHT	K15580 oppA, mppA	oligopeptide transport system substrate-binding protein
5. tr|T2GND4|T2GND4_MYCHT	K15581 oppB	oligopeptide transport system permease protein
6. tr|T2GRM1|T2GRM1_MYCHT	K00873 PK, pyk	pyruvate kinase [EC:2.7.1.40]
7. tr|T2GRM1|T2GRM1_MYCHT	K00873 PK, pyk	pyruvate kinase [EC:2.7.1.40]
8. tr|T2GRM1|T2GRM1_MYCHT	K00873 PK, pyk	pyruvate kinase [EC:2.7.1.40]
9. tr|T2GRM1|T2GRM1_MYCHT	K00873 PK, pyk	pyruvate kinase [EC:2.7.1.40]
10. tr|T2GTH6|T2GTH6_MYCHT	K03587 ftsI	cell division protein FtsI (penicillin-binding protein 3) [EC:3.4.16.4]
11. tr|T2GUU2|T2GUU2_MYCHT	K01929 murF	UDP-N-acetylmuramoyl-tripeptide--D-alanyl-D-alanine ligase [EC:6.3.2.10]
12. tr|T2GV53|T2GV53_MYCHT	K02563 murG	UDP-N-acetylglucosamine--N-acetylmuramyl-(pentapeptide) pyrophosphoryl-undecaprenol N-acetylglucosamine transferase [EC:2.4.1.227]
13. tr|T2GVH4|T2GVH4_MYCHT	K00121 frmA, ADH5, adhC	S-(hydroxymethyl)glutathione dehydrogenase / alcohol dehydrogenase [EC:1.1.1.284 1.1.1.1]
14. tr|T2GW34|T2GW34_MYCHT	K01000 mraY	phospho-N-acetylmuramoyl-pentapeptide-transferase [EC:2.7.8.13]
15. tr|T2GYG5|T2GYG5_MYCHT	K02040 pstS	phosphate transport system substrate-binding protein
16. tr|T2GYU6|T2GYU6_MYCHT	K01775 alr	alanine racemase [EC:5.1.1.1]
17. tr|T2GYZ6|T2GYZ6_MYCHT	K02040 pstS	phosphate transport system substrate-binding protein
18. tr|T2GZK5|T2GZK5_MYCHT	K04043 dnaK, HSPA9	molecular chaperone DnaK
19. tr|T2GZV0|T2GZV0_MYCHT	K03671 trxA	thioredoxin 1
