# Supplementary material for: Genome Subtraction and Comparison for the Identification of Novel Drug Targets against Mycobacterium avium subsp. hominissuis
Source: Pathogens. 2020 May 12;9(5):368. doi: 10.3390/pathogens9050368 (PMC7281720; doi:10.3390/pathogens9050368)
Supplement: Supplementary file 1 [file pathogens-09-00368-s001.zip › pathogens-729691-supplementary/Supplementary file/supplementary table S4.doc]

Supplementary table 4.

**ESSENTIAL NON-HOMOLOGOUS PROTEINS IN MAH STRAIN A5 INVOLVED IN DIFFERENT METABOLIC PATHWAYS AND OTHER CELLULAR ACTIVITIES**

**IDs of protein, KEGG Orthology number (KO) Gene and Protein Names:**

**Metabolism:**

1. **Carbohydrate Metabolism (Glycolysis/Gluconeogenesis, Citrate cycle (TCA cycle), Pentose phosphate pathway, Fructose and mannose metabolism, Galactose metabolism, Ascorbate and aldarate metabolism, Starch and sucrose metabolism, Amino sugar and nucleotide sugar metabolism, Pyruvate metabolism, Glyoxylate and dicarboxylate metabolism, Propanoate metabolism, Butanoate metabolism)**
1. tr|A0A0E2WGP9|A0A0E2WGP9_MYCAV; K01810 GPI, pgi; glucose-6-phosphate isomerase [EC:5.3.1.9]
2. tr|A0A0E2WUB4|A0A0E2WUB4_MYCAV; K02446 glpX; fructose-1,6-bisphosphatase II [EC:3.1.3.11]
3. tr|A0A0E2WBJ9|A0A0E2WBJ9_MYCAV; K00873 PK, pyk; pyruvate kinase [EC:2.7.1.40]
4. tr|A0A0E2W7T5|A0A0E2W7T5_MYCAV; K00174 korA, oorA, oforA; 2-oxoglutarate/2-oxoacid ferredoxin oxidoreductase subunit alpha [EC:1.2.7.3 1.2.7.11]
5. tr|A0A0E2WQZ0|A0A0E2WQZ0_MYCAV; K00175 korB, oorB, oforB; 2-oxoglutarate/2-oxoacid ferredoxin oxidoreductase subunit beta [EC:1.2.7.3 1.2.7.11]
6. tr|A0A0E2W3B4|A0A0E2W3B4_MYCAV; K00121 frmA, ADH5, adhC; S-(hydroxymethyl)glutathione dehydrogenase / alcohol dehydrogenase [EC:1.1.1.284 1.1.1.1]
7. tr|A0A0E2WML2|A0A0E2WML2_MYCAV; K00886 ppgK; polyphosphate glucokinase [EC:2.7.1.63]
8. tr|A0A0E2WNA9|A0A0E2WNA9_MYCAV; K01681 ACO, acnA; aconitate hydratase [EC:4.2.1.3]
9. tr|A0A0E2WHK3|A0A0E2WHK3_MYCAV; K00031 IDH1, IDH2, icd; isocitrate dehydrogenase [EC:1.1.1.42]
10. tr|A0A0E2W7T5|A0A0E2W7T5_MYCAV; K00174 korA, oorA, oforA; 2-oxoglutarate/2-oxoacid ferredoxin oxidoreductase subunit alpha [EC:1.2.7.3 1.2.7.11]
11. tr|A0A0E2WQZ0|A0A0E2WQZ0_MYCAV; K00175 korB, oorB, oforB; 2-oxoglutarate/2-oxoacid ferredoxin oxidoreductase subunit beta [EC:1.2.7.3 1.2.7.11]
12. tr|A0A0E2WFI8|A0A0E2WFI8_MYCAV; K00024 mdh; malate dehydrogenase [EC:1.1.1.37]
13. tr|A0A0E2WGP9|A0A0E2WGP9_MYCAV; K01810 GPI, pgi; glucose-6-phosphate isomerase [EC:5.3.1.9]
14. tr|A0A0E2W4U2|A0A0E2W4U2_MYCAV; K01783 rpe, RPE; ribulose-phosphate 3-epimerase [EC:5.1.3.1]
15. tr|A0A0E2WQY0|A0A0E2WQY0_MYCAV; K01808 rpiB; ribose 5-phosphate isomerase B [EC:5.3.1.6]
16. tr|A0A0E2WUB4|A0A0E2WUB4_MYCAV; K02446 glpX; fructose-1,6-bisphosphatase II [EC:3.1.3.11]
17. tr|A0A0E2W4U2|A0A0E2W4U2_MYCAV; K01783 rpe, RPE; ribulose-phosphate 3-epimerase [EC:5.1.3.1]
18. tr|A0A0E2W437|A0A0E2W437_MYCAV; K00966 GMPP; mannose-1-phosphate guanylyltransferase [EC:2.7.7.13]
19. tr|A0A0E2WUB4|A0A0E2WUB4_MYCAV; K02446 glpX; fructose-1,6-bisphosphatase II [EC:3.1.3.11]
20. tr|A0A0E2WTR4|A0A0E2WTR4_MYCAV; K00045 E1.1.1.67, mtlK; mannitol 2-dehydrogenase [EC:1.1.1.67]
21. tr|A0A0E2W0Q2|A0A0E2W0Q2_MYCAV; K00045 E1.1.1.67, mtlK; mannitol 2-dehydrogenase [EC:1.1.1.67]
22. tr|A0A0E2WQY0|A0A0E2WQY0_MYCAV; K01808 rpiB; ribose 5-phosphate isomerase B [EC:5.3.1.6]
23. tr|A0A0E2WJU8|A0A0E2WJU8_MYCAV; K00849 galK; galactokinase [EC:2.7.1.6]
24. tr|A0A0E2WVS6|A0A0E2WVS6_MYCAV; K01854 glf; UDP-galactopyranose mutase [EC:5.4.99.9]
25. tr|A0A0E2W056|A0A0E2W056_MYCAV; K05349 bglX; beta-glucosidase [EC:3.2.1.21]
26. tr|A0A0E2W3L9|A0A0E2W3L9_MYCAV; K16149 K16149; 1,4-alpha-glucan branching enzyme [EC:2.4.1.18]
27. tr|A0A0E2W723|A0A0E2W723_MYCAV; K00705 malQ; 4-alpha-glucanotransferase [EC:2.4.1.25]
28. tr|A0A0E2WJ77|A0A0E2WJ77_MYCAV; K16146 pep2; maltokinase [EC:2.7.1.175]
29. tr|A0A0E2W970|A0A0E2W970_MYCAV; K16147 glgE; starch synthase (maltosyl-transferring) [EC:2.4.99.16]
30. tr|A0A0E2WBQ2|A0A0E2WBQ2_MYCAV; K06044 treY, glgY; (1->4)-alpha-D-glucan 1-alpha-D-glucosylmutase [EC:5.4.99.15]
31. tr|A0A0E2WBH2|A0A0E2WBH2_MYCAV; K00697 otsA; trehalose 6-phosphate synthase [EC:2.4.1.15 .4.1.347]
32. tr|A0A0E2W3X2|A0A0E2W3X2_MYCAV; K01087 otsB; trehalose 6-phosphate phosphatase [EC:3.1.3.12]
33. tr|A0A0E2WGP9|A0A0E2WGP9_MYCAV; K01810 GPI, pgi; glucose-6-phosphate isomerase [EC:5.3.1.9]
34. tr|A0A0E2W7C2|A0A0E2W7C2_MYCAV; K00790 murA; UDP-N-acetylglucosamine 1-carboxyvinyltransferase [EC:2.5.1.7]
35. tr|A0A0E2W1W0|A0A0E2W1W0_MYCAV; K00075 murB; UDP-N-acetylmuramate dehydrogenase [EC:1.3.1.98]
36. tr|A0A0E2WML2|A0A0E2WML2_MYCAV; K00886 ppgK; polyphosphate glucokinase [EC:2.7.1.63]
37. tr|A0A0E2WGP9|A0A0E2WGP9_MYCAV; K01810 GPI, pgi; glucose-6-phosphate isomerase [EC:5.3.1.9]
38. tr|A0A0E2WJU8|A0A0E2WJU8_MYCAV; K00849 galK; galactokinase [EC:2.7.1.6]
39. tr|A0A0E2WVS6|A0A0E2WVS6_MYCAV; K01854 glf; UDP-galactopyranose mutase [EC:5.4.99.9]
40. tr|A0A0E2W437|A0A0E2W437_MYCAV; K00966 GMPP; mannose-1-phosphate guanylyltransferase [EC:2.7.7.13]
41. tr|A0A0E2W7T5|A0A0E2W7T5_MYCAV; K00174 korA, oorA, oforA; 2-oxoglutarate/2-oxoacid ferredoxin oxidoreductase subunit alpha [EC:1.2.7.3 1.2.7.11]
42. tr|A0A0E2WQZ0|A0A0E2WQZ0_MYCAV; K00175 korB, oorB, oforB; 2-oxoglutarate/2-oxoacid ferredoxin oxidoreductase subunit beta [EC:1.2.7.3 1.2.7.11]
43. tr|A0A0E2W1E8|A0A0E2W1E8_MYCAV; K00925 ackA; acetate kinase [EC:2.7.2.1]
44. tr|A0A0E2W830|A0A0E2W830_MYCAV; K13788 pta; phosphate acetyltransferase [EC:2.3.1.8]
45. tr|A0A0E2WBJ9|A0A0E2WBJ9_MYCAV; K00873 PK, pyk; pyruvate kinase [EC:2.7.1.40]
46. tr|A0A0E2WFI8|A0A0E2WFI8_MYCAV; K00024 mdh; malate dehydrogenase [EC:1.1.1.37]
47. tr|A0A0E2WAT7|A0A0E2WAT7_MYCAV; K01595 ppc; phosphoenolpyruvate carboxylase [EC:4.1.1.31]
48. tr|A0A0E2WUE8|A0A0E2WUE8_MYCAV; K01006 ppdK; pyruvate, orthophosphate dikinase [EC:2.7.9.1]
49. tr|A0A0E2W8M7|A0A0E2W8M7_MYCAV; K01638 aceB, glcB; malate synthase [EC:2.3.3.9]
50. tr|A0A0E2WCJ7|A0A0E2WCJ7_MYCAV; K01649 leuA, IMS; 2-isopropylmalate synthase [EC:2.3.3.13]
51. tr|A0A0E2W168|A0A0E2W168_MYCAV; K01637 E4.1.3.1, aceA; isocitrate lyase [EC:4.1.3.1]
52. tr|A0A0E2W6H1|A0A0E2W6H1_MYCAV; K01637 E4.1.3.1, aceA; isocitrate lyase [EC:4.1.3.1]
53. tr|A0A0E2W8M7|A0A0E2W8M7_MYCAV; K01638 aceB, glcB; malate synthase [EC:2.3.3.9]
54. tr|A0A0E2WFI8|A0A0E2WFI8_MYCAV; K00024 mdh; malate dehydrogenase [EC:1.1.1.37]
55. tr|A0A0E2WNA9|A0A0E2WNA9_MYCAV; K01681 ACO, acnA; aconitate hydratase [EC:4.2.1.3]
56. tr|A0A0E2WF00|A0A0E2WF00_MYCAV; K00865 glxK, garK; glycerate 2-kinase [EC:2.7.1.165]
57. tr|A0A0E2WCK2|A0A0E2WCK2_MYCAV; K00123 fdoG, fdhF, fdwA; formate dehydrogenase major subunit [EC:1.17.1.9]
58. tr|A0A0E2WWJ2|A0A0E2WWJ2_MYCAV; K00123 fdoG, fdhF, fdwA; formate dehydrogenase major subunit [EC:1.17.1.9]
59. tr|A0A0E2WE64|A0A0E2WE64_MYCAV; K00124 fdoH, fdsB; formate dehydrogenase iron-sulfur subunit
60. tr|A0A0E2W1E8|A0A0E2W1E8_MYCAV; K00925 ackA; acetate kinase [EC:2.7.2.1]
61. tr|A0A0E2W830|A0A0E2W830_MYCAV; K13788 pta; phosphate acetyltransferase [EC:2.3.1.8]
62. tr|A0A0E2W6T2|A0A0E2W6T2_MYCAV; K00249 ACADM, acd; acyl-CoA dehydrogenase [EC:1.3.8.7]
63. tr|A0A0E2WPJ7|A0A0E2WPJ7_MYCAV; K00249 ACADM, acd; acyl-CoA dehydrogenase [EC:1.3.8.7]
64. tr|A0A0E2WA17|A0A0E2WA17_MYCAV; K00140 mmsA, iolA, ALDH6A1; malonate-semialdehyde dehydrogenase (acetylating) / methylmalonate-semialdehyde dehydrogenase [EC:1.2.1.18 1.2.1.27]
65. tr|A0A0E2WCG6|A0A0E2WCG6_MYCAV; K03417 prpB; methylisocitrate lyase [EC:4.1.3.30]
66. tr|A0A0E2W3H9|A0A0E2W3H9_MYCAV; K00074 paaH, hbd, fadB, mmgB; 3-hydroxybutyryl-CoA dehydrogenase [EC:1.1.1.157]
67. tr|A0A0E2W7T5|A0A0E2W7T5_MYCAV; K00174 korA, oorA, oforA; 2-oxoglutarate/2-oxoacid ferredoxin oxidoreductase subunit alpha [EC:1.2.7.3 1.2.7.11]
68. tr|A0A0E2WQZ0|A0A0E2WQZ0_MYCAV; K00175 korB, oorB, oforB; 2-oxoglutarate/2-oxoacid ferredoxin oxidoreductase subunit beta [EC:1.2.7.3 1.2.7.11]
69. tr|A0A0E2W383|A0A0E2W383_MYCAV; K01653 E2.2.1.6S, ilvH, ilvN; acetolactate synthase I/III small subunit [EC:2.2.1.6]
70. tr|A0A0E2W383|A0A0E2W383_MYCAV; K01653 E2.2.1.6S, ilvH, ilvN; acetolactate synthase I/III small subunit [EC:2.2.1.6]
71. tr|A0A0E2W7U8|A0A0E2W7U8_MYCAV; K01704 leuD, IPMI-S; 3-isopropylmalate/(R)-2-methylmalate dehydratase small subunit [EC:4.2.1.33 4.2.1.35]
72. tr|A0A0E2W367|A0A0E2W367_MYCAV; K01704 leuD, IPMI-S; 3-isopropylmalate/(R)-2-methylmalate dehydratase small subunit [EC:4.2.1.33 4.2.1.35]
73. tr|A0A0E2WCU5|A0A0E2WCU5_MYCAV; K01858 INO1, ISYNA1; myo-inositol-1-phosphate synthase [EC:5.5.1.4]
74. tr|A0A0E2WA17|A0A0E2WA17_MYCAV; K00140 mmsA, iolA, ALDH6A1; malonate-semialdehyde dehydrogenase (acetylating) / methylmalonate-semialdehyde dehydrogenase [EC:1.2.1.18 1.2.1.27]

2. Energy Metabolism (Oxidative phosphorylation, Photosynthesis, Carbon fixation in photosynthetic organisms, Carbon fixation pathways in prokaryotes, Methane metabolism, Nitrogen metabolism, Sulfur metabolism)

1. tr|A0A0E2WL68|A0A0E2WL68_MYCAV; K00330 nuoA; NADH-quinone oxidoreductase subunit A [EC:7.1.1.2]
2. tr|A0A0E2W903|A0A0E2W903_MYCAV; K00339 nuoJ; NADH-quinone oxidoreductase subunit J [EC:7.1.1.2]
3. tr|A0A0E2W362|A0A0E2W362_MYCAV; K00340 nuoK; NADH-quinone oxidoreductase subunit K [EC:7.1.1.2]
4. tr|A0A0E2W294|A0A0E2W294_MYCAV; K03885 ndh; NADH dehydrogenase [EC:1.6.99.3]
5. tr|A0A0E2W6P7|A0A0E2W6P7_MYCAV; K03885 ndh; NADH dehydrogenase [EC:1.6.99.3]
6. tr|A0A0E2WS09|A0A0E2WS09_MYCAV; K03889 qcrC; ubiquinol-cytochrome c reductase cytochrome c subunit
7. tr|A0A0E2W8N7|A0A0E2W8N7_MYCAV; K03890 qcrA; ubiquinol-cytochrome c reductase iron-sulfur subunit
8. tr|A0A0E2W5A3|A0A0E2W5A3_MYCAV; K02259 COX15, ctaA; cytochrome c oxidase assembly protein subunit 15
9. tr|A0A0E2W552|A0A0E2W552_MYCAV; K00425 cydA; cytochrome bd ubiquinol oxidase subunit I [EC:7.1.1.7]
10. tr|A0A0E2WBJ5|A0A0E2WBJ5_MYCAV; K00426 cydB; cytochrome bd ubiquinol oxidase subunit II [EC:7.1.1.7]
11. tr|A0A0E2W931|A0A0E2W931_MYCAV; K02114 ATPF1E, atpC; F-type H+-transporting ATPase subunit epsilon
12. tr|A0A0E2W7B2|A0A0E2W7B2_MYCAV; K02108 ATPF0A, atpB; F-type H+-transporting ATPase subunit a
13. tr|A0A0E2W923|A0A0E2W923_MYCAV; K02109 ATPF0B, atpF; F-type H+-transporting ATPase subunit b
14. tr|A0A0E2WQF1|A0A0E2WQF1_MYCAV; K02110 ATPF0C, atpE; F-type H+-transporting ATPase subunit c
15. tr|A0A0E2W413|A0A0E2W413_MYCAV; K00937 ppk; polyphosphate kinase [EC:2.7.4.1]
16. tr|A0A0E2W2L0|A0A0E2W2L0_MYCAV; K22468 ppk2; polyphosphate kinase [EC:2.7.4.1]
17. tr|A0A0E2W931|A0A0E2W931_MYCAV; K02114 ATPF1E, atpC; F-type H+-transporting ATPase subunit psilon
18. tr|A0A0E2W923|A0A0E2W923_MYCAV; K02109 ATPF0B, atpF; F-type H+-transporting ATPase subunit b
19. tr|A0A0E2WQF1|A0A0E2WQF1_MYCAV; K02110 ATPF0C, atpE; F-type H+-transporting ATPase subunit c
20. tr|A0A0E2W7B2|A0A0E2W7B2_MYCAV; K02108 ATPF0A, atpB; F-type H+-transporting ATPase subunit a
21. tr|A0A0E2WUB4|A0A0E2WUB4_MYCAV; K02446 glpX; fructose-1,6-bisphosphatase II [EC:3.1.3.11]
22. tr|A0A0E2WQY0|A0A0E2WQY0_MYCAV; K01808 rpiB; ribose 5-phosphate isomerase B [EC:5.3.1.6]
23. tr|A0A0E2W4U2|A0A0E2W4U2_MYCAV; K01783 rpe, RPE; ribulose-phosphate 3-epimerase [EC:5.1.3.1]
24. tr|A0A0E2WAT7|A0A0E2WAT7_MYCAV; K01595 ppc; phosphoenolpyruvate carboxylase [EC:4.1.1.31]
25. tr|A0A0E2WUE8|A0A0E2WUE8_MYCAV; K01006 ppdK; pyruvate, orthophosphate dikinase [EC:2.7.9.1]
26. tr|A0A0E2WFI8|A0A0E2WFI8_MYCAV; K00024 mdh; malate dehydrogenase [EC:1.1.1.37]
27. tr|A0A0E2WUE8|A0A0E2WUE8_MYCAV; K01006 ppdK; pyruvate, orthophosphate dikinase [EC:2.7.9.1]
28. tr|A0A0E2WAT7|A0A0E2WAT7_MYCAV; K01595 ppc; phosphoenolpyruvate carboxylase [EC:4.1.1.31]
29. tr|A0A0E2WFI8|A0A0E2WFI8_MYCAV; K00024 mdh; malate dehydrogenase [EC:1.1.1.37]
30. tr|A0A0E2W7T5|A0A0E2W7T5_MYCAV; K00174 korA, oorA, oforA; 2-oxoglutarate/2-oxoacid ferredoxin oxidoreductase subunit alpha [EC:1.2.7.3 1.2.7.11]
31. tr|A0A0E2WQZ0|A0A0E2WQZ0_MYCAV; K00175 korB, oorB, oforB; 2-oxoglutarate/2-oxoacid ferredoxin oxidoreductase subunit beta [EC:1.2.7.3 1.2.7.11]
32. tr|A0A0E2WHK3|A0A0E2WHK3_MYCAV; K00031 IDH1, IDH2, icd; isocitrate dehydrogenase [EC:1.1.1.42]
33. tr|A0A0E2WNA9|A0A0E2WNA9_MYCAV; K01681 ACO, acnA; aconitate hydratase [EC:4.2.1.3]
34. tr|A0A0E2W830|A0A0E2W830_MYCAV; K13788 pta; phosphate acetyltransferase [EC:2.3.1.8]
35. tr|A0A0E2W1E8|A0A0E2W1E8_MYCAV; K00925 ackA; acetate kinase [EC:2.7.2.1]
36. tr|A0A0E2W3B4|A0A0E2W3B4_MYCAV; K00121 frmA, ADH5, adhC; S-(hydroxymethyl)glutathione dehydrogenase / alcohol dehydrogenase [EC:1.1.1.284 1.1.1.1]
37. tr|A0A0E2WCK2|A0A0E2WCK2_MYCAV; K00123 fdoG, fdhF, fdwA; formate dehydrogenase major subunit [EC:1.17.1.9]
38. tr|A0A0E2WWJ2|A0A0E2WWJ2_MYCAV; K00123 fdoG, fdhF, fdwA; formate dehydrogenase major subunit [EC:1.17.1.9]
39. tr|A0A0E2WE64|A0A0E2WE64_MYCAV; K00124 fdoH, fdsB; formate dehydrogenase iron-sulfur subunit
40. tr|A0A0E2WAT7|A0A0E2WAT7_MYCAV; K01595 ppc; phosphoenolpyruvate carboxylase [EC:4.1.1.31]
41. tr|A0A0E2WFI8|A0A0E2WFI8_MYCAV; K00024 mdh; malate dehydrogenase [EC:1.1.1.37]
42. tr|A0A0E2WUB4|A0A0E2WUB4_MYCAV; K02446 glpX; fructose-1,6-bisphosphatase II [EC:3.1.3.11]
43. tr|A0A0E2W1E8|A0A0E2W1E8_MYCAV; K00925 ackA; acetate kinase [EC:2.7.2.1]
44. tr|A0A0E2W830|A0A0E2W830_MYCAV; K13788 pta; phosphate acetyltransferase [EC:2.3.1.8]
45. tr|A0A0E2W9K7|A0A0E2W9K7_MYCAV; K11779 fbiC; FO synthase [EC:2.5.1.147 4.3.1.32]
46. tr|A0A0E2WSS4|A0A0E2WSS4_MYCAV; K00370 narG, narZ, nxrA; nitrate reductase / nitrite oxidoreductase, alpha subunit [EC:1.7.5.1 1.7.99.-]
47. tr|A0A0E2WB68|A0A0E2WB68_MYCAV; K00371 narH, narY, nxrB; nitrate reductase / nitrite oxidoreductase, beta subunit [EC:1.7.5.1 1.7.99.-]
48. tr|A0A0E2W4M0|A0A0E2W4M0_MYCAV; K04561 norB; nitric oxide reductase subunit B [EC:1.7.2.5]
49. tr|A0A0E2W5X9|A0A0E2W5X9_MYCAV; K00459 ncd2, npd; nitronate monooxygenase [EC:1.13.12.16]
50. tr|A0A0E2W7S2|A0A0E2W7S2_MYCAV; K15371 GDH2; glutamate dehydrogenase [EC:1.4.1.2]
51. tr|A0A0E2WVP3|A0A0E2WVP3_MYCAV; K00265 gltB; glutamate synthase (NADPH) large chain [EC:1.4.1.13]
52. tr|A0A0E2WDP8|A0A0E2WDP8_MYCAV; K00266 gltD; glutamate synthase (NADPH) small chain [EC:1.4.1.13]
53. tr|A0A0E2W0U2|A0A0E2W0U2_MYCAV; K01725 cynS; cyanate lyase [EC:4.2.1.104]
54. tr|A0A0E2WD67|A0A0E2WD67_MYCAV; K01673 cynT, can; carbonic anhydrase [EC:4.2.1.1]
55. tr|A0A0E2W753|A0A0E2W753_MYCAV; K01673 cynT, can; carbonic anhydrase [EC:4.2.1.1]
56. tr|A0A0E2W7X3|A0A0E2W7X3_MYCAV; K23163 sbp; sulfate/thiosulfate transport system substrate-binding protein
57. tr|A0A0E2WR38|A0A0E2WR38_MYCAV; K02046 cysU; sulfate/thiosulfate transport system permease protein
58. tr|A0A0E2W9S5|A0A0E2W9S5_MYCAV; K02047 cysW; sulfate/thiosulfate transport system permease protein
59. tr|A0A0E2WPD9|A0A0E2WPD9_MYCAV; K15553 ssuA; sulfonate transport system substrate-binding protein
60. tr|A0A0E2WCE0|A0A0E2WCE0_MYCAV; K15554 ssuC; sulfonate transport system permease protein
61. tr|A0A0E2WX33|A0A0E2WX33_MYCAV; K15554 ssuC; sulfonate transport system permease protein
62. tr|A0A0E2W8Y9|A0A0E2W8Y9_MYCAV; K00957 cysD; sulfate adenylyltransferase subunit 2 [EC:2.7.7.4]
63. tr|A0A0E2W8G4|A0A0E2W8G4_MYCAV; K00390 cysH; phosphoadenosine phosphosulfate reductase [EC:1.8.4.8 1.8.4.10]
64. tr|A0A0E2WED7|A0A0E2WED7_MYCAV; K00390 cysH; phosphoadenosine phosphosulfate reductase [EC:1.8.4.8 1.8.4.10]
65. tr|A0A0E2W7Y7|A0A0E2W7Y7_MYCAV; K00392 sir; sulfite reductase (ferredoxin) [EC:1.8.7.1]
66. tr|A0A0E2WES7|A0A0E2WES7_MYCAV; K00392 sir; sulfite reductase (ferredoxin) [EC:1.8.7.1]
67. tr|A0A0E2W9Y9|A0A0E2W9Y9_MYCAV; K00640 cysE; serine O-acetyltransferase [EC:2.3.1.30]
68. tr|A0A0E2WD26|A0A0E2WD26_MYCAV; K00641 metX; homoserine O-acetyltransferase/O-succinyltransferase [EC:2.3.1.31 2.3.1.46]

3. Lipid Metabolism (Fatty acid biosynthesis, Primary bile acid biosynthesis, Secondary bile acid biosynthesis, Glycerolipid metabolism, Glycerophospholipid metabolism)

1. tr|A0A0E2WPV2|A0A0E2WPV2_MYCAV; K02078 acpP; acyl carrier protein
2. tr|A0A0E2WA10|A0A0E2WA10_MYCAV; K00648 fabH; 3-oxoacyl-[acyl-carrier-protein] synthase III [EC:2.3.1.180]
3. tr|A0A0E2WEE0|A0A0E2WEE0_MYCAV; K03921 FAB2, SSI2, desA1; acyl-[acyl-carrier-protein] desaturase [EC:1.14.19.2 1.14.19.11 1.14.19.26]
4. tr|A0A0E2WBJ3|A0A0E2WBJ3_MYCAV; K03921 FAB2, SSI2, desA1; acyl-[acyl-carrier-protein] desaturase [EC:1.14.19.2 1.14.19.11 1.14.19.26]
5. tr|A0A0E2WUB1|A0A0E2WUB1_MYCAV; K03922 desA2; acyl-[acyl-carrier-protein] desaturase [EC:1.14.19.2]
6. tr|A0A0E2W6T2|A0A0E2W6T2_MYCAV; K00249 ACADM, acd; acyl-CoA dehydrogenase [EC:1.3.8.7]
7. tr|A0A0E2WPJ7|A0A0E2WPJ7_MYCAV; K00249 ACADM, acd; acyl-CoA dehydrogenase [EC:1.3.8.7]
8. tr|A0A0E2WMM5|A0A0E2WMM5_MYCAV; K00255 ACADL; long-chain-acyl-CoA dehydrogenase [EC:1.3.8.8]
9. tr|A0A0E2W3B4|A0A0E2W3B4_MYCAV; K00121 frmA, ADH5, adhC; S-(hydroxymethyl)glutathione dehydrogenase / alcohol dehydrogenase [EC:1.1.1.284 1.1.1.1]
10. tr|A0A0E2WF00|A0A0E2WF00_MYCAV; K00865 glxK, garK; glycerate 2-kinase [EC:2.7.1.165]
11. tr|A0A0E2W5T5|A0A0E2W5T5_MYCAV; K00635 tgs, wax-dgat; diacylglycerol O-acyltransferase / wax synthase [EC:2.3.1.20 2.3.1.75]
12. tr|A0A0E2WN60|A0A0E2WN60_MYCAV; K00635 tgs, wax-dgat; diacylglycerol O-acyltransferase / wax synthase [EC:2.3.1.20 2.3.1.75]
13. tr|A0A0E2WIK8|A0A0E2WIK8_MYCAV; K00635 tgs, wax-dgat; diacylglycerol O-acyltransferase / wax synthase [EC:2.3.1.20 2.3.1.75]
14. tr|A0A0E2W7R8|A0A0E2W7R8_MYCAV; K00635 tgs, wax-dgat; diacylglycerol O-acyltransferase / wax synthase [EC:2.3.1.20 2.3.1.75]
15. tr|A0A0E2WKP1|A0A0E2WKP1_MYCAV; K00635 tgs, wax-dgat; diacylglycerol O-acyltransferase / wax synthase [EC:2.3.1.20 2.3.1.75]
16. tr|A0A0E2WKI7|A0A0E2WKI7_MYCAV; K00635 tgs, wax-dgat; diacylglycerol O-acyltransferase / wax synthase [EC:2.3.1.20 2.3.1.75]
17. tr|A0A0E2W8I5|A0A0E2W8I5_MYCAV; K18851 fbp; diacylglycerol O-acyltransferase / trehalose O-mycolyltransferase [EC:2.3.1.20 2.3.1.122]
18. tr|A0A0E2W6U1|A0A0E2W6U1_MYCAV; K18851 fbp; diacylglycerol O-acyltransferase / trehalose O-mycolyltransferase [EC:2.3.1.20 2.3.1.122]
19. tr|A0A0E2W702|A0A0E2W702_MYCAV; K01046 lip, TGL2; triacylglycerol lipase [EC:3.1.1.3]
20. tr|A0A0E2W7T3|A0A0E2W7T3_MYCAV; K17103 CHO1, pssA; CDP-diacylglycerol---serine O-phosphatidyltransferase [EC:2.7.8.8]
21. tr|A0A0E2W194|A0A0E2W194_MYCAV; K01613 psd, PISD; phosphatidylserine decarboxylase [EC:4.1.1.65]
22. tr|A0A0E2WA14|A0A0E2WA14_MYCAV; K00995 pgsA, PGS1; CDP-diacylglycerol---glycerol-3-phosphate 3-phosphatidyltransferase [EC:2.7.8.5]
23. tr|A0A0E2WRS9|A0A0E2WRS9_MYCAV; K00803 AGPS, agpS; alkyldihydroxyacetonephosphate synthase [EC:2.5.1.26]
24. tr|A0A0E2WEE0|A0A0E2WEE0_MYCAV; K03921 FAB2, SSI2, desA1; acyl-[acyl-carrier-protein] desaturase [EC:1.14.19.2 1.14.19.11 1.14.19.26]
25. tr|A0A0E2WBJ3|A0A0E2WBJ3_MYCAV; K03921 FAB2, SSI2, desA1; acyl-[acyl-carrier-protein] desaturase [EC:1.14.19.2 1.14.19.11 1.14.19.26]
26. tr|A0A0E2WUB1|A0A0E2WUB1_MYCAV; K03922 desA2; acyl-[acyl-carrier-protein] desaturase [EC:1.14.19.2]

4. Nucleotide Metabolism (Purine metabolism, Pyrimidine metabolism)

1. tr|A0A0E2WBA9|A0A0E2WBA9_MYCAV; K23264 purS; phosphoribosylformylglycinamidine synthase subunit PurS [EC:6.3.5.3]
2. tr|A0A0E2WKJ1|A0A0E2WKJ1_MYCAV; K01239 iunH; purine nucleosidase [EC:3.2.2.1]
3. tr|A0A0E2W1P4|A0A0E2W1P4_MYCAV; K00088 IMPDH, guaB; IMP dehydrogenase [EC:1.1.1.205]
4. tr|A0A0E2WAR0|A0A0E2WAR0_MYCAV; K02428 rdgB; XTP/dITP diphosphohydrolase [EC:3.6.1.66]
5. tr|A0A0E2WBJ9|A0A0E2WBJ9_MYCAV; K00873 PK, pyk; pyruvate kinase [EC:2.7.1.40]
6. tr|A0A0E2WEJ8|A0A0E2WEJ8_MYCAV; K01129 dgt; dGTPase [EC:3.1.5.1]
7. tr|A0A0E2W125|A0A0E2W125_MYCAV; K01524 ppx-gppA; exopolyphosphatase / guanosine-5'-triphosphate,3'-diphosphate pyrophosphatase [EC:3.6.1.11 3.6.1.40]
8. tr|A0A0E2WAR5|A0A0E2WAR5_MYCAV; K01524 ppx-gppA; exopolyphosphatase / guanosine-5'-triphosphate,3'-diphosphate pyrophosphatase [EC:3.6.1.11 3.6.1.40]
9. tr|A0A0E2WAD3|A0A0E2WAD3_MYCAV; K00856 E2.7.1.20, ADK; adenosine kinase [EC:2.7.1.20]
10. tr|A0A0E2W8Y9|A0A0E2W8Y9_MYCAV; K00957 cysD; sulfate adenylyltransferase subunit 2 [EC:2.7.7.4]
11. tr|A0A0E2WT12|A0A0E2WT12_MYCAV; K16839 hpxO; FAD-dependent urate hydroxylase [EC:1.14.13.113]
12. tr|A0A0E2W5G4|A0A0E2W5G4_MYCAV; K09903 pyrH; uridylate kinase [EC:2.7.4.22]
13. tr|A0A0E2W6Y9|A0A0E2W6Y9_MYCAV; K00945 cmk; CMP/dCMP kinase [EC:2.7.4.25]
14. tr|A0A0E2WJL7|A0A0E2WJL7_MYCAV; K01494 dcd; dCTP deaminase [EC:3.5.4.13]
15. tr|A0A0E2WMN8|A0A0E2WMN8_MYCAV; K03465 thyX, thy1; thymidylate synthase (FAD) [EC:2.1.1.148]

5. Amino Acid Metabolism (Alanine, aspartate and glutamate metabolism, Glycine, serine and threonine metabolism, Cysteine and methionine metabolism, Valine, leucine and isoleucine biosynthesis, Lysine biosynthesis, Lysine degradation, Arginine and proline metabolism, Histidine metabolism, Tyrosine metabolism, Phenylalanine metabolism, Phenylalanine, Tyrosine and tryptophan biosynthesis)

1. tr|A0A0E2WVP3|A0A0E2WVP3_MYCAV; K00265 gltB; glutamate synthase (NADPH) large chain [EC:1.4.1.13]
2. tr|A0A0E2WDP8|A0A0E2WDP8_MYCAV; K00266 gltD; glutamate synthase (NADPH) small chain [EC:1.4.1.13]
3. tr|A0A0E2W7S2|A0A0E2W7S2_MYCAV; K15371 GDH2; glutamate dehydrogenase [EC:1.4.1.2]
4. tr|A0A0E2WIN2|A0A0E2WIN2_MYCAV; K00928 lysC; aspartate kinase [EC:2.7.2.4]
5. tr|A0A0E2W7A1|A0A0E2W7A1_MYCAV; K00003 hom; homoserine dehydrogenase [EC:1.1.1.3]
6. tr|A0A0E2W915|A0A0E2W915_MYCAV; K00872 thrB1; homoserine kinase [EC:2.7.1.39]
7. tr|A0A0E2WF00|A0A0E2WF00_MYCAV; K00865 glxK, garK; glycerate 2-kinase [EC:2.7.1.165]
8. tr|A0A0E2W7T3|A0A0E2W7T3_MYCAV; K17103 CHO1, pssA; CDP-diacylglycerol---serine O-phosphatidyltransferase [EC:2.7.8.8]
9. tr|A0A0E2W5P2|A0A0E2W5P2_MYCAV; K01754 E4.3.1.19, ilvA, tdcB; threonine dehydratase [EC:4.3.1.19]
10. tr|A0A0E2W7A2|A0A0E2W7A2_MYCAV; K01695 trpA; tryptophan synthase alpha chain [EC:4.2.1.20]
11. tr|A0A0E2W589|A0A0E2W589_MYCAV; K01696 trpB; tryptophan synthase beta chain [EC:4.2.1.20]
12. tr|A0A0E2W9Y9|A0A0E2W9Y9_MYCAV; K00640 cysE; serine O-acetyltransferase [EC:2.3.1.30]
13. tr|A0A0E2WH64|A0A0E2WH64_MYCAV; K00549 metE; 5-methyltetrahydropteroyltriglutamate--homocysteine methyltransferase [EC:2.1.1.14]
14. tr|A0A0E2WIN2|A0A0E2WIN2_MYCAV; K00928 lysC; aspartate kinase [EC:2.7.2.4]
15. tr|A0A0E2W7A1|A0A0E2W7A1_MYCAV; K00003 hom; homoserine dehydrogenase [EC:1.1.1.3]
16. tr|A0A0E2WD26|A0A0E2WD26_MYCAV; K00641 metX; homoserine O-acetyltransferase/O-succinyltransferase [EC:2.3.1.31 2.3.1.46]
17. tr|A0A0E2WIN5|A0A0E2WIN5_MYCAV; K01919 gshA; glutamate--cysteine ligase [EC:6.3.2.2]
18. tr|A0A0E2WFI8|A0A0E2WFI8_MYCAV; K00024 mdh; malate dehydrogenase [EC:1.1.1.37]
19. tr|A0A0E2W6T2|A0A0E2W6T2_MYCAV; K00249 ACADM, acd; acyl-CoA dehydrogenase [EC:1.3.8.7]
20. tr|A0A0E2WPJ7|A0A0E2WPJ7_MYCAV; K00249 ACADM, acd; acyl-CoA dehydrogenase [EC:1.3.8.7]
21. tr|A0A0E2WA17|A0A0E2WA17_MYCAV; K00140 mmsA, iolA, ALDH6A1; malonate-semialdehyde dehydrogenase (acetylating) / methylmalonate-semialdehyde dehydrogenase [EC:1.2.1.18 1.2.1.27]
22. tr|A0A0E2W5P2|A0A0E2W5P2_MYCAV; K01754 E4.3.1.19, ilvA, tdcB; threonine dehydratase [EC:4.3.1.19]
23. tr|A0A0E2W7U8|A0A0E2W7U8_MYCAV; K01704 leuD, IPMI-S; 3-isopropylmalate/(R)-2-methylmalate dehydratase small subunit [EC:4.2.1.33 4.2.1.35]
24. tr|A0A0E2W367|A0A0E2W367_MYCAV; K01704 leuD, IPMI-S; 3-isopropylmalate/(R)-2-methylmalate dehydratase small subunit [EC:4.2.1.33 4.2.1.35]
25. tr|A0A0E2W383|A0A0E2W383_MYCAV; K01653 E2.2.1.6S, ilvH, ilvN; acetolactate synthase I/III small subunit [EC:2.2.1.6]
26. tr|A0A0E2W5P1|A0A0E2W5P1_MYCAV; K00053 ilvC; ketol-acid reductoisomerase [EC:1.1.1.86]
27. tr|A0A0E2W6I0|A0A0E2W6I0_MYCAV; K01687 ilvD; dihydroxy-acid dehydratase [EC:4.2.1.9]
28. tr|A0A0E2WCJ7|A0A0E2WCJ7_MYCAV; K01649 leuA, IMS; 2-isopropylmalate synthase [EC:2.3.3.13]
29. tr|A0A0E2W7A1|A0A0E2W7A1_MYCAV; K00003 hom; homoserine dehydrogenase [EC:1.1.1.3]
30. tr|A0A0E2WIN2|A0A0E2WIN2_MYCAV; K00928 lysC; aspartate kinase [EC:2.7.2.4]
31. tr|A0A0E2W7K2|A0A0E2W7K2_MYCAV; K01714 dapA; 4-hydroxy-tetrahydrodipicolinate synthase [EC:4.3.3.7]
32. tr|A0A0E2W3G8|A0A0E2W3G8_MYCAV; K00215 dapB; 4-hydroxy-tetrahydrodipicolinate reductase [EC:1.17.1.8]
33. tr|A0A0E2W6L0|A0A0E2W6L0_MYCAV; K00215 dapB; 4-hydroxy-tetrahydrodipicolinate reductase [EC:1.17.1.8]
34. tr|A0A0E2W5V4|A0A0E2W5V4_MYCAV; K00215 dapB; 4-hydroxy-tetrahydrodipicolinate reductase [EC:1.17.1.8]
35. tr|A0A0E2WFQ6|A0A0E2WFQ6_MYCAV; K00674 dapD; 2,3,4,5-tetrahydropyridine-2,6-dicarboxylate N-succinyltransferase [EC:2.3.1.117]
36. tr|A0A0E2W465|A0A0E2W465_MYCAV; K01778 dapF; diaminopimelate epimerase [EC:5.1.1.7]
37. tr|A0A0E2W8S1|A0A0E2W8S1_MYCAV; K01928 murE; UDP-N-acetylmuramoyl-L-alanyl-D-glutamate--2,6-diaminopimelate ligase [EC:6.3.2.13]
38. tr|A0A0E2WS61|A0A0E2WS61_MYCAV; K01929 murF; UDP-N-acetylmuramoyl-tripeptide--D-alanyl-D-alanine ligase [EC:6.3.2.10]
39. tr|A0A0E2W990|A0A0E2W990_MYCAV; K21672 ord; 2,4-diaminopentanoate dehydrogenase [EC:1.4.1.12 1.4.1.26]
40. tr|A0A0E2WAH7|A0A0E2WAH7_MYCAV; K21672 ord; 2,4-diaminopentanoate dehydrogenase [EC:1.4.1.12 1.4.1.26]
41. tr|A0A0E2WCI0|A0A0E2WCI0_MYCAV; K21672 ord; 2,4-diaminopentanoate dehydrogenase [EC:1.4.1.12 1.4.1.26]
42. tr|A0A0E2WCH1|A0A0E2WCH1_MYCAV; K21672 ord; 2,4-diaminopentanoate dehydrogenase [EC:1.4.1.12 1.4.1.26]
43. tr|A0A0E2W7Z1|A0A0E2W7Z1_MYCAV; K21672 ord; 2,4-diaminopentanoate dehydrogenase [EC:1.4.1.12 1.4.1.26]
44. tr|A0A0E2W6P9|A0A0E2W6P9_MYCAV; K21672 ord; 2,4-diaminopentanoate dehydrogenase [EC:1.4.1.12 1.4.1.26]
45. tr|A0A0E2WC94|A0A0E2WC94_MYCAV; K01478 arcA; arginine deiminase [EC:3.5.3.6]
46. tr|A0A0E2W7S2|A0A0E2W7S2_MYCAV; K15371 GDH2; glutamate dehydrogenase [EC:1.4.1.2]
47. tr|A0A0E2WAE0|A0A0E2WAE0_MYCAV; K00619 argA; amino-acid N-acetyltransferase [EC:2.3.1.1]
48. tr|A0A0E2W4Z7|A0A0E2W4Z7_MYCAV; K00620 argJ; glutamate N-acetyltransferase / amino-acid N-acetyltransferase [EC:2.3.1.35 2.3.1.1]
49. tr|A0A0E2W732|A0A0E2W732_MYCAV; K00930 argB; acetylglutamate kinase [EC:2.7.2.8]
50. tr|A0A0E2WBG0|A0A0E2WBG0_MYCAV; K00145 argC; N-acetyl-gamma-glutamyl-phosphate reductase [EC:1.2.1.38]
51. tr|A0A0E2W9N7|A0A0E2W9N7_MYCAV; K01470 E3.5.2.10; creatinine amidohydrolase [EC:3.5.2.10]
52. tr|A0A0E2WB42|A0A0E2WB42_MYCAV; K00318 PRODH, fadM, putB; proline dehydrogenase [EC:1.5.5.2]
53. tr|A0A0E2W990|A0A0E2W990_MYCAV; K21672 ord; 2,4-diaminopentanoate dehydrogenase [EC:1.4.1.12 .4.1.26]
54. tr|A0A0E2WAH7|A0A0E2WAH7_MYCAV; K21672 ord; 2,4-diaminopentanoate dehydrogenase [EC:1.4.1.12 1.4.1.26]
55. tr|A0A0E2WCI0|A0A0E2WCI0_MYCAV; K21672 ord; 2,4-diaminopentanoate dehydrogenase [EC:1.4.1.12 1.4.1.26]
56. tr|A0A0E2WCH1|A0A0E2WCH1_MYCAV; K21672 ord; 2,4-diaminopentanoate dehydrogenase [EC:1.4.1.12 1.4.1.26]
57. D	tr|A0A0E2W7Z1|A0A0E2W7Z1_MYCAV; K21672 ord; 2,4-diaminopentanoate dehydrogenase [EC:1.4.1.12 1.4.1.26]
58. tr|A0A0E2W6P9|A0A0E2W6P9_MYCAV; K21672 ord; 2,4-diaminopentanoate dehydrogenase [EC:1.4.1.12 1.4.1.26]
59. tr|A0A0E2W660|A0A0E2W660_MYCAV; K00765 hisG; ATP phosphoribosyltransferase [EC:2.4.2.17]
60. tr|A0A0E2W7V3|A0A0E2W7V3_MYCAV; K01523 hisE; phosphoribosyl-ATP pyrophosphohydrolase [EC:3.6.1.31]
61. tr|A0A0E2WBM5|A0A0E2WBM5_MYCAV; K01496 hisI; phosphoribosyl-AMP cyclohydrolase [EC:3.5.4.19]
62. tr|A0A0E2W5A5|A0A0E2W5A5_MYCAV; K02501 hisH; imidazole glycerol-phosphate synthase subunit HisH [EC:4.3.2.10]
63. tr|A0A0E2W5L1|A0A0E2W5L1_MYCAV; K02500 hisF; imidazole glycerol-phosphate synthase subunit HisF [EC:4.3.2.10]
64. tr|A0A0E2WBN0|A0A0E2WBN0_MYCAV; K01693 hisB; imidazoleglycerol-phosphate dehydratase [EC:4.2.1.19]
65. tr|A0A0E2WRV3|A0A0E2WRV3_MYCAV; K00817 hisC; histidinol-phosphate aminotransferase [EC:2.6.1.9]
66. tr|A0A0E2W5L5|A0A0E2W5L5_MYCAV; K00817 hisC; histidinol-phosphate aminotransferase [EC:2.6.1.9]
67. tr|A0A0E2WNU9|A0A0E2WNU9_MYCAV; K00013 hisD; histidinol dehydrogenase [EC:1.1.1.23]
68. tr|A0A0E2WE49|A0A0E2WE49_MYCAV; K18911 egtD; L-histidine Nalpha-methyltransferase [EC:2.1.1.44]
69. tr|A0A0E2WRV3|A0A0E2WRV3_MYCAV; K00817 hisC; histidinol-phosphate aminotransferase [EC:2.6.1.9]
70. tr|A0A0E2W5L5|A0A0E2W5L5_MYCAV; K00817 hisC; histidinol-phosphate aminotransferase [EC:2.6.1.9]
71. tr|A0A0E2W3B4|A0A0E2W3B4_MYCAV; K00121 frmA, ADH5, adhC; S-(hydroxymethyl)glutathione dehydrogenase / alcohol dehydrogenase [EC:1.1.1.284 1.1.1.1]
72. tr|A0A0E2W3H9|A0A0E2W3H9_MYCAV; K00074 paaH, hbd, fadB, mmgB; 3-hydroxybutyryl-CoA dehydrogenase [EC:1.1.1.157]
73. tr|A0A0E2WRV3|A0A0E2WRV3_MYCAV; K00817 hisC; histidinol-phosphate aminotransferase [EC:2.6.1.9]
74. tr|A0A0E2W5L5|A0A0E2W5L5_MYCAV; K00817 hisC; histidinol-phosphate aminotransferase [EC:2.6.1.9]
75. tr|A0A0E2WPM6|A0A0E2WPM6_MYCAV; K03782 katG; catalase-peroxidase [EC:1.11.1.21]
76. tr|A0A0E2WA69|A0A0E2WA69_MYCAV; K05714 mhpC; 2-hydroxy-6-oxonona-2,4-dienedioate hydrolase [EC:3.7.1.14]
77. tr|A0A0E2WPM6|A0A0E2WPM6_MYCAV; K03782 katG; catalase-peroxidase [EC:1.11.1.21]
78. tr|A0A0E2WR93|A0A0E2WR93_MYCAV; K01626 E2.5.1.54, aroF, aroG, aroH; 3-deoxy-7-phosphoheptulonate synthase [EC:2.5.1.54]
79. tr|A0A0E2WAF0|A0A0E2WAF0_MYCAV; K01626 E2.5.1.54, aroF, aroG, aroH; 3-deoxy-7-phosphoheptulonate synthase [EC:2.5.1.54]
80. tr|A0A0E2WMZ4|A0A0E2WMZ4_MYCAV; K01735 aroB; 3-dehydroquinate synthase [EC:4.2.3.4]
81. tr|A0A0E2W505|A0A0E2W505_MYCAV; K03786 aroQ, qutE; 3-dehydroquinate dehydratase II [EC:4.2.1.10]
82. tr|A0A0E2W6I3|A0A0E2W6I3_MYCAV; K00014 aroE; shikimate dehydrogenase [EC:1.1.1.25]
83. tr|A0A0E2W1U4|A0A0E2W1U4_MYCAV; K00800 aroA; 3-phosphoshikimate 1-carboxyvinyltransferase [EC:2.5.1.19]
84. tr|A0A0E2W4G7|A0A0E2W4G7_MYCAV; K01736 aroC; chorismate synthase [EC:4.2.3.5]
85. tr|A0A0E2WNU3|A0A0E2WNU3_MYCAV; K01657 trpE; anthranilate synthase component I [EC:4.1.3.27]
86. tr|A0A0E2WFF2|A0A0E2WFF2_MYCAV; K00766 trpD; anthranilate phosphoribosyltransferase [EC:2.4.2.18]
87. tr|A0A0E2W7B1|A0A0E2W7B1_MYCAV; K01817 trpF; phosphoribosylanthranilate isomerase [EC:5.3.1.24]
88. tr|A0A0E2WBM0|A0A0E2WBM0_MYCAV; K01609 trpC; indole-3-glycerol phosphate synthase [EC:4.1.1.48]
89. tr|A0A0E2W7A2|A0A0E2W7A2_MYCAV; K01695 trpA; tryptophan synthase alpha chain [EC:4.2.1.20]
90. tr|A0A0E2W589|A0A0E2W589_MYCAV; K01696 trpB; tryptophan synthase beta chain [EC:4.2.1.20]
91. tr|A0A0E2WIJ1|A0A0E2WIJ1_MYCAV; K04517 tyrA2; prephenate dehydrogenase [EC:1.3.1.12]
92. tr|A0A0E2WDR6|A0A0E2WDR6_MYCAV; K04518 pheA2; prephenate dehydratase [EC:4.2.1.51]
93. tr|A0A0E2WRV3|A0A0E2WRV3_MYCAV; K00817 hisC; histidinol-phosphate aminotransferase [EC:2.6.1.9]
94. tr|A0A0E2W5L5|A0A0E2W5L5_MYCAV; K00817 hisC; histidinol-phosphate aminotransferase [EC:2.6.1.9]

6. Metabolism of Other Amino Acids (beta-Alanine metabolism, Taurine and hypotaurine metabolism, Phosphonate and phosphinate metabolism, Selenocompound metabolism, Glutamine and D-glutamate metabolism, Arginine and D-ornithine metabolism, D-Alanine metabolism)

1. tr|A0A0E2WHQ4|A0A0E2WHQ4_MYCAV; K01579 panD; aspartate 1-decarboxylase [EC:4.1.1.11]
2. tr|A0A0E2WBP6|A0A0E2WBP6_MYCAV; K01918 panC; pantoate--beta-alanine ligase [EC:6.3.2.1]
3. tr|A0A0E2W6T2|A0A0E2W6T2_MYCAV; K00249 ACADM, acd; acyl-CoA dehydrogenase [EC:1.3.8.7]
4. tr|A0A0E2WPJ7|A0A0E2WPJ7_MYCAV; K00249 ACADM, acd; acyl-CoA dehydrogenase [EC:1.3.8.7]
5. tr|A0A0E2WA17|A0A0E2WA17_MYCAV; K00140 mmsA, iolA, ALDH6A1; malonate-semialdehyde dehydrogenase (acetylating) / methylmalonate-semialdehyde dehydrogenase [EC:1.2.1.18 1.2.1.27]
6. tr|A0A0E2W7S2|A0A0E2W7S2_MYCAV; K15371 GDH2; glutamate dehydrogenase [EC:1.4.1.2]
7. tr|A0A0E2W830|A0A0E2W830_MYCAV; K13788 pta; phosphate acetyltransferase [EC:2.3.1.8]
8. tr|A0A0E2W1E8|A0A0E2W1E8_MYCAV; K00925 ackA; acetate kinase [EC:2.7.2.1]
9. tr|A0A0E2WH64|A0A0E2WH64_MYCAV; K00549 metE; 5-methyltetrahydropteroyltriglutamate--homocysteine methyltransferase [EC:2.1.1.14]
10. tr|A0A0E2W6T8|A0A0E2W6T8_MYCAV; K11717 sufS; cysteine desulfurase / selenocysteine lyase [EC:2.8.1.7 4.4.1.16]
11. tr|A0A0E2W8Y9|A0A0E2W8Y9_MYCAV; K00957 cysD; sulfate adenylyltransferase subunit 2 [EC:2.7.7.4]
12. tr|A0A0E2W056|A0A0E2W056_MYCAV; K05349 bglX; beta-glucosidase [EC:3.2.1.21]
13. tr|A0A0E2W7H6|A0A0E2W7H6_MYCAV; K01776 murI; glutamate racemase [EC:5.1.1.3]
14. tr|A0A0E2WS70|A0A0E2WS70_MYCAV; K01924 murC; UDP-N-acetylmuramate--alanine ligase [EC:6.3.2.8]
15. tr|A0A0E2W8Y7|A0A0E2W8Y7_MYCAV; K01925 murD; UDP-N-acetylmuramoylalanine--D-glutamate ligase EC:6.3.2.9]
16. tr|A0A0E2W990|A0A0E2W990_MYCAV; K21672 ord; 2,4-diaminopentanoate dehydrogenase [EC:1.4.1.12 1.4.1.26]
17. tr|A0A0E2WAH7|A0A0E2WAH7_MYCAV; K21672 ord; 2,4-diaminopentanoate dehydrogenase [EC:1.4.1.12 1.4.1.26]
18. tr|A0A0E2WCI0|A0A0E2WCI0_MYCAV; K21672 ord; 2,4-diaminopentanoate dehydrogenase [EC:1.4.1.12 1.4.1.26]
19. tr|A0A0E2WCH1|A0A0E2WCH1_MYCAV; K21672 ord; 2,4-diaminopentanoate dehydrogenase [EC:1.4.1.12 1.4.1.26]
20. tr|A0A0E2W7Z1|A0A0E2W7Z1_MYCAV; K21672 ord; 2,4-diaminopentanoate dehydrogenase [EC:1.4.1.12 1.4.1.26]
21. tr|A0A0E2W6P9|A0A0E2W6P9_MYCAV; K21672 ord; 2,4-diaminopentanoate dehydrogenase [EC:1.4.1.12 1.4.1.26]
22. tr|A0A0E2W1R0|A0A0E2W1R0_MYCAV; K01775 alr; alanine racemase [EC:5.1.1.1]
23. tr|A0A0E2WIN5|A0A0E2WIN5_MYCAV; K01919 gshA; glutamate--cysteine ligase [EC:6.3.2.2]
24. tr|A0A0E2WHK3|A0A0E2WHK3_MYCAV; K00031 IDH1, IDH2, icd; isocitrate dehydrogenase [EC:1.1.1.42]

8. Glycan Biosynthesis and Metabolism (Lipopolysaccharide biosynthesis, Peptidoglycan biosynthesis, Lipoarabinomannan (LAM) biosynthesis, Arabinogalactan biosynthesis)

1. tr|A0A0E2WTY5|A0A0E2WTY5_MYCAV; K03271 gmhA, lpcA; D-sedoheptulose 7-phosphate isomerase [EC:5.3.1.28]
2. tr|A0A0E2W2D9|A0A0E2W2D9_MYCAV; K03271 gmhA, lpcA; D-sedoheptulose 7-phosphate isomerase [EC:5.3.1.28]
3. tr|A0A0E2WC90|A0A0E2WC90_MYCAV; K03273 gmhB; D-glycero-D-manno-heptose 1,7-bisphosphate phosphatase [EC:3.1.3.82 3.1.3.83]
4. tr|A0A0E2W7C2|A0A0E2W7C2_MYCAV; K00790 murA; UDP-N-acetylglucosamine 1-carboxyvinyltransferase [EC:2.5.1.7]
5. tr|A0A0E2W1W0|A0A0E2W1W0_MYCAV; K00075 murB; UDP-N-acetylmuramate dehydrogenase [EC:1.3.1.98]
6. tr|A0A0E2WS70|A0A0E2WS70_MYCAV; K01924 murC; UDP-N-acetylmuramate--alanine ligase [EC:6.3.2.8]
7. tr|A0A0E2W8Y7|A0A0E2W8Y7_MYCAV; K01925 murD; UDP-N-acetylmuramoylalanine--D-glutamate ligase [EC:6.3.2.9]
8. tr|A0A0E2W8S1|A0A0E2W8S1_MYCAV; K01928 murE; UDP-N-acetylmuramoyl-L-alanyl-D-glutamate--2,6-diaminopimelate ligase [EC:6.3.2.13]
9. tr|A0A0E2WS61|A0A0E2WS61_MYCAV; K01929 murF; UDP-N-acetylmuramoyl-tripeptide--D-alanyl-D-alanine ligase [EC:6.3.2.10]
10. tr|A0A0E2W6U8|A0A0E2W6U8_MYCAV; K06153 bacA; undecaprenyl-diphosphatase [EC:3.6.1.27]
11. tr|A0A0E2WAI2|A0A0E2WAI2_MYCAV; K01000 mraY; phospho-N-acetylmuramoyl-pentapeptide-transferase [EC:2.7.8.13]
12. tr|A0A0E2W8X9|A0A0E2W8X9_MYCAV; K02563 murG; UDP-N-acetylglucosamine--N-acetylmuramyl-(pentapeptide) pyrophosphoryl-undecaprenol N-acetylglucosamine transferase [EC:2.4.1.227]
13. tr|A0A0E2WF36|A0A0E2WF36_MYCAV; K03587 ftsI; cell division protein FtsI (penicillin-binding protein 3) [EC:3.4.16.4]
14. tr|A0A0E2WWW1|A0A0E2WWW1_MYCAV; K05364 pbpA; penicillin-binding protein A
15. tr|A0A0E2W2U8|A0A0E2W2U8_MYCAV; K07258 dacC, dacA, dacD; serine-type D-Ala-D-Ala carboxypeptidase (penicillin-binding protein 5/6) [EC:3.4.16.4]
16. tr|A0A0E2W332|A0A0E2W332_MYCAV; K07258 dacC, dacA, dacD; serine-type D-Ala-D-Ala carboxypeptidase (penicillin-binding protein 5/6) [EC:3.4.16.4]
17. tr|A0A0E2WDK8|A0A0E2WDK8_MYCAV; K07259 dacB; serine-type D-Ala-D-Ala carboxypeptidase/endopeptidase (penicillin-binding protein 4) [EC:3.4.16.4 3.4.21.-]
18. tr|A0A0E2W3K2|A0A0E2W3K2_MYCAV; K22311 ptfP1; phosphatidylinositol dimannoside acyltransferase [EC:2.3.1.265]
19. tr|A0A0E2WB62|A0A0E2WB62_MYCAV; K13669 pimE; alpha-1,2-mannosyltransferase [EC:2.4.1.-]
20. tr|A0A0E2W5H5|A0A0E2W5H5_MYCAV; K14340 K14340; mannosyltransferase [EC:2.4.1.-]
21. tr|A0A0E2WS27|A0A0E2WS27_MYCAV; K14337 mptA; alpha-1,6-mannosyltransferase [EC:2.4.1.-]
22. tr|A0A0E2W6U7|A0A0E2W6U7_MYCAV; K14339 mptB; alpha-1,6-mannosyltransferase [EC:2.4.1.-]
23. tr|A0A0E2WF23|A0A0E2WF23_MYCAV; K13671 K13671; alpha-1,2-mannosyltransferase [EC:2.4.1.-]
24. tr|A0A0E2WVU5|A0A0E2WVU5_MYCAV; K11387 embC; arabinosyltransferase C [EC:2.4.2.-]
25. tr|A0A0E2WC62|A0A0E2WC62_MYCAV; K13687 aftB; arabinofuranosyltransferase [EC:2.4.2.-]
26. tr|A0A0E2WMK0|A0A0E2WMK0_MYCAV; K16647 aftC; arabinofuranan 3-O-arabinosyltransferase [EC:2.4.2.47]
27. tr|A0A0E2WIR7|A0A0E2WIR7_MYCAV; K16648 aftD; arabinofuranan 3-O-arabinosyltransferase [EC:2.4.2.-]
28. tr|A0A0E2W3C1|A0A0E2W3C1_MYCAV; K23176 mtxT; methylthioxylose transferase
29. tr|A0A0E2W1Y1|A0A0E2W1Y1_MYCAV; K16870 wbbL; N-acetylglucosaminyl-diphospho-decaprenol L-rhamnosyltransferase [EC:2.4.1.289]
30. tr|A0A0E2WIG0|A0A0E2WIG0_MYCAV; K16649 glft1; rhamnopyranosyl-N-acetylglucosaminyl-diphospho-decaprenol beta-1,3/1,4-galactofuranosyltransferase [EC:2.4.1.287]
31. tr|A0A0E2WDT4|A0A0E2WDT4_MYCAV; K16650 glft2; galactofuranosylgalactofuranosylrhamnosyl-N-acetylglucosaminyl-diphospho-decaprenol beta-1,5/1,6-galactofuranosyltransferase [EC:2.4.1.288]
32. tr|A0A0E2WDU8|A0A0E2WDU8_MYCAV; K13686 aftA; galactan 5-O-arabinofuranosyltransferase [EC:2.4.2.46]
33. tr|A0A0E2WC62|A0A0E2WC62_MYCAV; K13687 aftB; arabinofuranosyltransferase [EC:2.4.2.-]
34. tr|A0A0E2WMK0|A0A0E2WMK0_MYCAV; K16647 aftC; arabinofuranan 3-O-arabinosyltransferase [EC:2.4.2.47]
35. tr|A0A0E2WDU4|A0A0E2WDU4_MYCAV; K11385 embA; arabinosyltransferase A [EC:2.4.2.-]
36. tr|A0A0E2WIR7|A0A0E2WIR7_MYCAV; K16648 aftD; arabinofuranan 3-O-arabinosyltransferase [EC:2.4.2.-]
37. tr|A0A0E2W8I5|A0A0E2W8I5_MYCAV; K18851 fbp; diacylglycerol O-acyltransferase / trehalose O-mycolyltransferase [EC:2.3.1.20 2.3.1.122]
38. tr|A0A0E2W6U1|A0A0E2W6U1_MYCAV; K18851 fbp; diacylglycerol O-acyltransferase / trehalose O-mycolyltransferase [EC:2.3.1.20 2.3.1.122]

9. Metabolism of Cofactors and Vitamins (Thiamine metabolism, Riboflavin metabolism, Vitamin B6 metabolism, Nicotinate and Nicotinamide metabolism Pantothenate and CoA biosynthesis, Biotin metabolism Folate biosynthesis, Porphyrin and chlorophyll metabolism Ubiquinone and other Terpenoid-quinone biosynthesis)

1. tr|A0A0E2WKD4|A0A0E2WKD4_MYCAV; K03147 thiC; phosphomethylpyrimidine synthase [EC:4.1.99.17]
2. tr|A0A0E2W3N8|A0A0E2W3N8_MYCAV; K00941 thiD; hydroxymethylpyrimidine/phosphomethylpyrimidine kinase [EC:2.7.1.49 2.7.4.7]
3. tr|A0A0E2WCS5|A0A0E2WCS5_MYCAV; K03151 thiI; tRNA uracil 4-sulfurtransferase [EC:2.8.1.4]
4. tr|A0A0E2W816|A0A0E2W816_MYCAV; K03149 thiG; thiazole synthase [EC:2.8.1.10]
5. tr|A0A0E2W3P6|A0A0E2W3P6_MYCAV; K00788 thiE; thiamine-phosphate pyrophosphorylase [EC:2.5.1.3]
6. tr|A0A0E2WM10|A0A0E2WM10_MYCAV; K00946 thiL; thiamine-monophosphate kinase [EC:2.7.4.16]
7. tr|A0A0E2W4S9|A0A0E2W4S9_MYCAV; K14652 ribBA; 3,4-dihydroxy 2-butanone 4-phosphate synthase / GTP cyclohydrolase II [EC:4.1.99.12 3.5.4.25]
8. tr|A0A0E2WA71|A0A0E2WA71_MYCAV; K00082 ribD2; 5-amino-6-(5-phosphoribosylamino)uracil reductase [EC:1.1.1.193]
9. tr|A0A0E2WAJ2|A0A0E2WAJ2_MYCAV; K11752 ribD; diaminohydroxyphosphoribosylaminopyrimidine deaminase / 5-amino-6-(5-phosphoribosylamino)uracil reductase [EC:3.5.4.26 1.1.1.193]
10. tr|A0A0E2WAI7|A0A0E2WAI7_MYCAV; K00794 ribH, RIB4; 6,7-dimethyl-8-ribityllumazine synthase [EC:2.5.1.78]
11. tr|A0A0E2WMV3|A0A0E2WMV3_MYCAV; K00793 ribE, RIB5; riboflavin synthase [EC:2.5.1.9]
12. tr|A0A0E2W772|A0A0E2W772_MYCAV; K04719 bluB; 5,6-dimethylbenzimidazole synthase [EC:1.13.11.79]
13. tr|A0A0E2W483|A0A0E2W483_MYCAV; K06215 pdxS, pdx1; pyridoxal 5'-phosphate synthase pdxS subunit [EC:4.3.3.6]
14. tr|A0A0E2W3J8|A0A0E2W3J8_MYCAV; K08681 pdxT, pdx2; 5'-phosphate synthase pdxT subunit [EC:4.3.3.6]
15. tr|A0A0E2W5M0|A0A0E2W5M0_MYCAV; K03517 nadA; quinolinate synthase [EC:2.5.1.72]
16. tr|A0A0E2WR18|A0A0E2WR18_MYCAV; K00969 nadD; nicotinate-nucleotide adenylyltransferase [EC:2.7.7.18]
17. tr|A0A0E2WKJ1|A0A0E2WKJ1_MYCAV; K01239 iunH; purine nucleosidase [EC:3.2.2.1]
18. tr|A0A0E2WML9|A0A0E2WML9_MYCAV; K00322 sthA, udhA; NAD(P) transhydrogenase [EC:1.6.1.1]
19. tr|A0A0E2W383|A0A0E2W383_MYCAV; K01653 E2.2.1.6S, ilvH, ilvN; acetolactate synthase I/III small subunit [EC:2.2.1.6]
20. tr|A0A0E2W5P1|A0A0E2W5P1_MYCAV; K00053 ilvC; ketol-acid reductoisomerase [EC:1.1.1.86]
21. tr|A0A0E2W6I0|A0A0E2W6I0_MYCAV; K01687 ilvD; dihydroxy-acid dehydratase [EC:4.2.1.9]
22. tr|A0A0E2WEY1|A0A0E2WEY1_MYCAV; K00606 panB; 3-methyl-2-oxobutanoate hydroxymethyltransferase [EC:2.1.2.11]
23. tr|A0A0E2W0Z4|A0A0E2W0Z4_MYCAV; K00077 panE, apbA; 2-dehydropantoate 2-reductase [EC:1.1.1.169]
24. tr|A0A0E2WBP6|A0A0E2WBP6_MYCAV; K01918 panC; pantoate--beta-alanine ligase [EC:6.3.2.1]
25. tr|A0A0E2WBI8|A0A0E2WBI8_MYCAV; K03525 coaX; type III pantothenate kinase [EC:2.7.1.33]
26. tr|A0A0E2WM00|A0A0E2WM00_MYCAV; K00954 E2.7.7.3A, coaD, kdtB; pantetheine-phosphate adenylyltransferase [EC:2.7.7.3]
27. tr|A0A0E2WQU3|A0A0E2WQU3_MYCAV; K00997 acpS; holo-[acyl-carrier protein] synthase [EC:2.7.8.7]
28. tr|A0A0E2WHQ4|A0A0E2WHQ4_MYCAV; K01579 panD; aspartate 1-decarboxylase [EC:4.1.1.11]
29. tr|A0A0E2W7C9|A0A0E2W7C9_MYCAV; K01935 bioD; dethiobiotin synthetase [EC:6.3.3.3]
30. tr|A0A0E2W5M6|A0A0E2W5M6_MYCAV; K01012 bioB; biotin synthase [EC:2.8.1.6]
31. tr|A0A0E2W8L1|A0A0E2W8L1_MYCAV; K03644 lipA; lipoyl synthase [EC:2.8.1.8]
32. tr|A0A0E2WDM9|A0A0E2WDM9_MYCAV; K01633 folB; 7,8-dihydroneopterin aldolase/epimerase/oxygenase [EC:4.1.2.25 5.1.99.8 1.13.11.81]
33. tr|A0A0E2WC50|A0A0E2WC50_MYCAV; K00950 folK; 2-amino-4-hydroxy-6-hydroxymethyldihydropteridine diphosphokinase [EC:2.7.6.3]
34. tr|A0A0E2W9L5|A0A0E2W9L5_MYCAV; K00796 folP; dihydropteroate synthase [EC:2.5.1.15]
35. tr|A0A0E2WVN6|A0A0E2WVN6_MYCAV; K00796 folP; dihydropteroate synthase [EC:2.5.1.15]
36. tr|A0A0E2W4S9|A0A0E2W4S9_MYCAV; K14652 ribBA; 3,4-dihydroxy 2-butanone 4-phosphate synthase / GTP cyclohydrolase II [EC:4.1.99.12 3.5.4.25]
37. tr|A0A0E2WAN4|A0A0E2WAN4_MYCAV; K01665 pabB; para-aminobenzoate synthetase component I [EC:2.6.1.85]
38. tr|A0A0E2WUS6|A0A0E2WUS6_MYCAV; K02619 pabC; 4-amino-4-deoxychorismate lyase [EC:4.1.3.38]
39. tr|A0A0E2WMN8|A0A0E2WMN8_MYCAV; K03465 thyX, thy1; thymidylate synthase (FAD) [EC:2.1.1.148]
40. tr|A0A0E2W3B4|A0A0E2W3B4_MYCAV; K00121 frmA, ADH5, adhC; S-(hydroxymethyl)glutathione dehydrogenase / alcohol dehydrogenase [EC:1.1.1.284 1.1.1.1]
41. tr|A0A0E2W110|A0A0E2W110_MYCAV; K02492 hemA; glutamyl-tRNA reductase [EC:1.2.1.70]
42. tr|A0A0E2W0E3|A0A0E2W0E3_MYCAV; K01719 hemD, UROS; uroporphyrinogen-III synthase [EC:4.2.1.75]
43. tr|A0A0E2WK15|A0A0E2WK15_MYCAV; K13542 cobA-hemD; uroporphyrinogen III methyltransferase / synthase [EC:2.1.1.107 4.2.1.75]
44. tr|A0A0E2WLS4|A0A0E2WLS4_MYCAV; K02302 cysG; uroporphyrin-III C-methyltransferase / precorrin-2 dehydrogenase / sirohydrochlorin ferrochelatase [EC:2.1.1.107 1.3.1.76 4.99.1.4]
45. tr|A0A0E2W5A3|A0A0E2W5A3_MYCAV; K02259 COX15, ctaA; cytochrome c oxidase assembly protein subunit 15
46. tr|A0A0E2W7X6|A0A0E2W7X6_MYCAV; K13540 cobIJ; precorrin-2 C20-methyltransferase / precorrin-3B C17-methyltransferase [EC:2.1.1.130 2.1.1.131]
47. tr|A0A0E2WCD5|A0A0E2WCD5_MYCAV; K05936 cobM, cbiF; precorrin-4/cobalt-precorrin-4 C11-methyltransferase [EC:2.1.1.133 2.1.1.271]
48. tr|A0A0E2WTT3|A0A0E2WTT3_MYCAV; K02228 cobF; precorrin-6A synthase [EC:2.1.1.152]
49. tr|A0A0E2W638|A0A0E2W638_MYCAV; K05895 cobK-cbiJ; precorrin-6A/cobalt-precorrin-6A reductase [EC:1.3.1.54 1.3.1.106]
50. tr|A0A0E2W687|A0A0E2W687_MYCAV; K00595 cobL; precorrin-6Y C5,15-methyltransferase (decarboxylating) [EC:2.1.1.132]
51. tr|A0A0E2W693|A0A0E2W693_MYCAV; K06042 cobH-cbiC; precorrin-8X/cobalt-precorrin-8 methylmutase [EC:5.4.99.61 5.4.99.60]
52. tr|A0A0E2W5E6|A0A0E2W5E6_MYCAV; K02224 cobB-cbiA; cobyrinic acid a,c-diamide synthase [EC:6.3.5.9 6.3.5.11]
53. tr|A0A0E2W696|A0A0E2W696_MYCAV; K02230 cobN; cobaltochelatase CobN [EC:6.6.1.2]
54. tr|A0A0E2W2X7|A0A0E2W2X7_MYCAV; K19221 cobA, btuR; cob(I)alamin adenosyltransferase [EC:2.5.1.17]
55. tr|A0A0E2W5F5|A0A0E2W5F5_MYCAV; K02232 cobQ, cbiP; adenosylcobyric acid synthase [EC:6.3.5.10]
56. tr|A0A0E2WA98|A0A0E2WA98_MYCAV; K02227 cbiB, cobD; adenosylcobinamide-phosphate synthase [EC:6.3.1.10]
57. tr|A0A0E2WAC9|A0A0E2WAC9_MYCAV; K02231 cobP, cobU; adenosylcobinamide kinase / adenosylcobinamide-phosphate guanylyltransferase [EC:2.7.1.156 2.7.7.62]
58. tr|A0A0E2W8M2|A0A0E2W8M2_MYCAV; K02233 E2.7.8.26, cobS, cobV; adenosylcobinamide-GDP ribazoletransferase [EC:2.7.8.26]
59. tr|A0A0E2W9K2|A0A0E2W9K2_MYCAV; K03404 chlD, bchD; magnesium chelatase subunit D [EC:6.6.1.1]
60. tr|A0A0E2W2X5|A0A0E2W2X5_MYCAV; K02361 entC; isochorismate synthase [EC:5.4.4.2]
61. tr|A0A0E2W7I2|A0A0E2W7I2_MYCAV; K02551 menD; 2-succinyl-5-enolpyruvyl-6-hydroxy-3-cyclohexene-1-carboxylate synthase [EC:2.2.1.9]
62. tr|A0A0E2WJX6|A0A0E2WJX6_MYCAV; K01661 menB; naphthoate synthase [EC:4.1.3.36]
63. tr|A0A0E2WM20|A0A0E2WM20_MYCAV; K03809 wrbA; NAD(P)H dehydrogenase (quinone) [EC:1.6.5.2]

9. Metabolism of Terpenoids and Polyketides (Terpenoid backbone biosynthesis, Limonene and pinene degradation, Polyketide sugar unit biosynthesis, Biosynthesis of siderophore group nonribosomal peptides)

1. tr|A0A0E2W2Z6|A0A0E2W2Z6_MYCAV; K00099 dxr; 1-deoxy-D-xylulose-5-phosphate reductoisomerase [EC:1.1.1.267]
2. tr|A0A0E2WGW7|A0A0E2WGW7_MYCAV; K00919 ispE; 4-diphosphocytidyl-2-C-methyl-D-erythritol kinase [EC:2.7.1.148]
3. tr|A0A0E2WHT0|A0A0E2WHT0_MYCAV; K01770 ispF; 2-C-methyl-D-erythritol 2,4-cyclodiphosphate synthase [EC:4.6.1.12]
4. tr|A0A0E2WLU0|A0A0E2WLU0_MYCAV; K03526 gcpE, ispG; (E)-4-hydroxy-3-methylbut-2-enyl-diphosphate synthase [EC:1.17.7.1 1.17.7.3]
5. tr|A0A0E2WH47|A0A0E2WH47_MYCAV; K03527 ispH, lytB; 4-hydroxy-3-methylbut-2-en-1-yl diphosphate reductase [EC:1.17.7.4]
6. tr|A0A0E2W9C1|A0A0E2W9C1_MYCAV; K01823 idi, IDI; isopentenyl-diphosphate Delta-isomerase [EC:5.3.3.2]
7. tr|A0A0E2WRD6|A0A0E2WRD6_MYCAV; K14215 E2.5.1.86; trans,polycis-decaprenyl diphosphate synthase [EC:2.5.1.86]
8. tr|A0A0E2WVN5|A0A0E2WVN5_MYCAV; K14733 limB; limonene 1,2-monooxygenase [EC:1.14.13.107]
9. tr|A0A0E2W6N4|A0A0E2W6N4_MYCAV; K14733 limB; limonene 1,2-monooxygenase [EC:1.14.13.107]
10. tr|A0A0E2WBH3|A0A0E2WBH3_MYCAV; K01790 rfbC, rmlC; dTDP-4-dehydrorhamnose 3,5-epimerase [EC:5.1.3.13]
11. tr|A0A0E2W2X5|A0A0E2W2X5_MYCAV; K02361 entC; isochorismate synthase [EC:5.4.4.2]
12. tr|A0A0E2W821|A0A0E2W821_MYCAV; K04781 mbtI, irp9, ybtS; salicylate synthetase [EC:5.4.4.2 4.2.99.21]
13. tr|A0A0E2WSE5|A0A0E2WSE5_MYCAV; K04787 mbtA; mycobactin salicyl-AMP ligase [EC:6.3.2.-]
14. tr|A0A0E2W851|A0A0E2W851_MYCAV; K04793 mbtG; mycobactin lysine-N-oxygenase

10. Biosynthesis of Other Secondary Metabolites

1. tr|A0A0E2W056|A0A0E2W056_MYCAV; K05349 bglX; beta-glucosidase [EC:3.2.1.21]
2. tr|A0A0E2WPM6|A0A0E2WPM6_MYCAV; K03782 katG; catalase-peroxidase [EC:1.11.1.21]
3. tr|A0A0E2WRV3|A0A0E2WRV3_MYCAV; K00817 hisC; histidinol-phosphate aminotransferase [EC:2.6.1.9]
4. tr|A0A0E2W5L5|A0A0E2W5L5_MYCAV; K00817 hisC; histidinol-phosphate aminotransferase [EC:2.6.1.9]
5. tr|A0A0E2W8Y9|A0A0E2W8Y9_MYCAV; K00957 cysD; sulfate adenylyltransferase subunit 2 [EC:2.7.7.4]
6. tr|A0A0E2WIN2|A0A0E2WIN2_MYCAV; K00928 lysC; aspartate kinase [EC:2.7.2.4]
7. tr|A0A0E2W7K2|A0A0E2W7K2_MYCAV; K01714 dapA; 4-hydroxy-tetrahydrodipicolinate synthase [EC:4.3.3.7]
8. tr|A0A0E2W3G8|A0A0E2W3G8_MYCAV; K00215 dapB; 4-hydroxy-tetrahydrodipicolinate reductase [EC:1.17.1.8]
9. tr|A0A0E2W6L0|A0A0E2W6L0_MYCAV; K00215 dapB; 4-hydroxy-tetrahydrodipicolinate reductase [EC:1.17.1.8]
10. tr|A0A0E2W5V4|A0A0E2W5V4_MYCAV; K00215 dapB; 4-hydroxy-tetrahydrodipicolinate reductase [EC:1.17.1.8]
11. tr|A0A0E2WCU5|A0A0E2WCU5_MYCAV; K01858 INO1, ISYNA1; myo-inositol-1-phosphate synthase [EC:5.5.1.4]
12. tr|A0A0E2WBH3|A0A0E2WBH3_MYCAV; K01790 rfbC, rmlC; dTDP-4-dehydrorhamnose 3,5-epimerase [EC:5.1.3.13]
13. tr|A0A0E2WIJ1|A0A0E2WIJ1_MYCAV; K04517 tyrA2; prephenate dehydrogenase [EC:1.3.1.12]
14. tr|A0A0E2WRV3|A0A0E2WRV3_MYCAV; K00817 hisC; histidinol-phosphate aminotransferase [EC:2.6.1.9]
15. tr|A0A0E2W5L5|A0A0E2W5L5_MYCAV; K00817 hisC; histidinol-phosphate aminotransferase [EC:2.6.1.9]
16. tr|A0A0E2WNU3|A0A0E2WNU3_MYCAV; K01657 trpE; anthranilate synthase component I [EC:4.1.3.27]
17. tr|A0A0E2WPV2|A0A0E2WPV2_MYCAV; K02078 acpP; acyl carrier protein

11. Xenobiotics Biodegradation and Metabolism (Benzoate degradation, Aminobenzoate degradation, Xylene degradation, Ethylbenzene degradation, Atrazine degradation Dioxin degradation)

1. tr|A0A0E2W855|A0A0E2W855_MYCAV; K01607 pcaC; 4-carboxymuconolactone decarboxylase [EC:4.1.1.44]
2. tr|A0A0E2W3H9|A0A0E2W3H9_MYCAV; K00074 paaH, hbd, fadB, mmgB; 3-hydroxybutyryl-CoA dehydrogenase [EC:1.1.1.157]
3. tr|A0A0E2W3B4|A0A0E2W3B4_MYCAV; K00121 frmA, ADH5, adhC; S-(hydroxymethyl)glutathione dehydrogenase / alcohol dehydrogenase [EC:1.1.1.284 1.1.1.1]
4. tr|A0A0E2WDV2|A0A0E2WDV2_MYCAV; K06912 tfdA; alpha-ketoglutarate-dependent 2,4-dichlorophenoxyacetate dioxygenase [EC:1.14.11.-]
5. tr|A0A0E2W5N2|A0A0E2W5N2_MYCAV; K03379 chnB; cyclohexanone monooxygenase [EC:1.14.13.22]
6. tr|A0A0E2W7M0|A0A0E2W7M0_MYCAV; K06446 DCAA; acyl-CoA dehydrogenase [EC:1.3.99.-]
7. tr|A0A0E2W3B4|A0A0E2W3B4_MYCAV; K00121 frmA, ADH5, adhC; S-(hydroxymethyl)glutathione dehydrogenase / alcohol dehydrogenase [EC:1.1.1.284 1.1.1.1]
8. tr|A0A0E2WBU7|A0A0E2WBU7_MYCAV; K15981 CYP125A; cholest-4-en-3-one 26-monooxygenase [EC:1.14.15.29]
9. tr|A0A0E2W5V2|A0A0E2W5V2_MYCAV; K15982 kshA; 3-ketosteroid 9alpha-monooxygenase subunit A [EC:1.14.15.30]
10. tr|A0A0E2WBM3|A0A0E2WBM3_MYCAV; K16048 hsaB; 3-hydroxy-9,10-secoandrosta-1,3,5(10)-triene-9,17-dione monooxygenase reductase component [EC:1.5.1.-]
11. tr|A0A0E2WHW2|A0A0E2WHW2_MYCAV; K16049 hsaC; 3,4-dihydroxy-9,10-secoandrosta-1,3,5(10)-triene-9,17-dione 4,5-dioxygenase [EC:1.13.11.25]
12. tr|A0A0E2W7I0|A0A0E2W7I0_MYCAV; K16051 tesI; 3-oxo-5alpha-steroid 4-dehydrogenase [EC:1.3.99.5]
13. tr|A0A0E2W3B4|A0A0E2W3B4_MYCAV; K00121 frmA, ADH5, adhC; S-(hydroxymethyl)glutathione dehydrogenase / alcohol dehydrogenase [EC:1.1.1.284 1.1.1.1]
14. tr|A0A0E2W3B4|A0A0E2W3B4_MYCAV; K00121 frmA, ADH5, adhC; S-(hydroxymethyl)glutathione dehydrogenase / alcohol dehydrogenase [EC:1.1.1.284 1.1.1.1]
15. tr|A0A0E2W1P4|A0A0E2W1P4_MYCAV; K00088 IMPDH, guaB; IMP dehydrogenase [EC:1.1.1.205]
16. tr|A0A0E2WPM6|A0A0E2WPM6_MYCAV; K03782 katG; catalase-peroxidase [EC:1.11.1.21]

Genetic Information Processing:

12. Transcription (RNA polymerase)

1. tr|A0A0E2WT34|A0A0E2WT34_MYCAV; K03040 rpoA; DNA-directed RNA polymerase subunit alpha [EC:2.7.7.6]
2. tr|A0A0E2WMW4|A0A0E2WMW4_MYCAV; K03060 rpoZ; DNA-directed RNA polymerase subunit omega [EC:2.7.7.6]

13. Translation (Ribosome)

1. tr|A0A0E2WSX8|A0A0E2WSX8_MYCAV; K02982 RP-S3, rpsC; small subunit ribosomal protein S3
2. tr|A0A0E2W9X7|A0A0E2W9X7_MYCAV; K02986 RP-S4, rpsD; small subunit ribosomal protein S4
3. tr|A0A0E2WEF5|A0A0E2WEF5_MYCAV; K02990 RP-S6, MRPS6, rpsF; small subunit ribosomal protein S6
4. tr|A0A0E2WFU8|A0A0E2WFU8_MYCAV; K02961 RP-S17, MRPS17, rpsQ; small subunit ribosomal protein S17
5. tr|A0A0E2WR33|A0A0E2WR33_MYCAV; K02968 RP-S20, rpsT; small subunit ribosomal protein S20
6. tr|A0A0E2WJQ4|A0A0E2WJQ4_MYCAV; K02864 RP-L10, MRPL10, rplJ; large subunit ribosomal protein L10
7. tr|A0A0E2WBD9|A0A0E2WBD9_MYCAV; K02881 RP-L18, MRPL18, rplR; large subunit ribosomal protein 18
8. tr|A0A0E2W7U1|A0A0E2W7U1_MYCAV; K02888 RP-L21, MRPL21, rplU; large subunit ribosomal protein L21
9. tr|A0A0E2W9P6|A0A0E2W9P6_MYCAV; K02890 RP-L22, MRPL22, rplV; large subunit ribosomal protein L22
10. tr|A0A0E2WCC1|A0A0E2WCC1_MYCAV; K02897 RP-L25, rplY; large subunit ribosomal protein L25
11. tr|A0A0E2W408|A0A0E2W408_MYCAV; K02902 RP-L28, MRPL28, rpmB; large subunit ribosomal protein L28
12. tr|A0A0E2W698|A0A0E2W698_MYCAV; K02902 RP-L28, MRPL28, rpmB; large subunit ribosomal protein L28
13. tr|A0A0E2W9W9|A0A0E2W9W9_MYCAV; K02904 RP-L29, rpmC; large subunit ribosomal protein L29
14. tr|A0A0E2WCM9|A0A0E2WCM9_MYCAV; K02907 RP-L30, MRPL30, rpmD; large subunit ribosomal protein L30
15. tr|A0A0E2WDN1|A0A0E2WDN1_MYCAV; K02909 RP-L31, rpmE; large subunit ribosomal protein L31
16. tr|A0A0E2W1I6|A0A0E2W1I6_MYCAV; K02913 RP-L33, MRPL33, rpmG; large subunit ribosomal protein L33
17. tr|A0A0E2W745|A0A0E2W745_MYCAV; K02914 RP-L34, MRPL34, rpmH; large subunit ribosomal protein L34
18. tr|A0A0E2W741|A0A0E2W741_MYCAV; K02916 RP-L35, MRPL35, rpmI; large subunit ribosomal protein L35
19. tr|A0A0E2WBH7|A0A0E2WBH7_MYCAV; K02919 RP-L36, MRPL36, rpmJ; large subunit ribosomal protein L36
20. tr|A0A0E2WM41|A0A0E2WM41_MYCAV; K02435 gatC, GATC; aspartyl-tRNA(Asn)/glutamyl-tRNA(Gln) amidotransferase subunit C [EC:6.3.5.6 6.3.5.7]
21. tr|A0A0E2WBK7|A0A0E2WBK7_MYCAV; K01883 CARS, cysS; cysteinyl-tRNA synthetase [EC:6.1.1.16]

14. Folding, Sorting and Degradation (Protein export, Sulfur relay system, Proteasome, RNA degradation )

1. tr|A0A0E2WJR7|A0A0E2WJR7_MYCAV; K03073 secE; preprotein translocase subunit SecE
2. tr|A0A0E2WN40|A0A0E2WN40_MYCAV; K03072 secD; preprotein translocase subunit SecD
3. tr|A0A0E2W538|A0A0E2W538_MYCAV; K03074 secF; preprotein translocase subunit SecF
4. tr|A0A0E2W472|A0A0E2W472_MYCAV; K03210 yajC; preprotein translocase subunit YajC
5. tr|A0A0E2WJH1|A0A0E2WJH1_MYCAV; K03217 yidC, spoIIIJ, OXA1, ccfA; YidC/Oxa1 family membrane protein insertase
6. tr|A0A0E2WPX2|A0A0E2WPX2_MYCAV; K03070 secA; preprotein translocase subunit SecA [EC:7.4.2.8]
7. tr|A0A0E2WCC7|A0A0E2WCC7_MYCAV; K03116 tatA; sec-independent protein translocase protein TatA
8. tr|A0A0E2W633|A0A0E2W633_MYCAV; K03118 tatC; sec-independent protein translocase protein TatC
9. tr|A0A0E2WBR7|A0A0E2WBR7_MYCAV; K03101 lspA; signal peptidase II [EC:3.4.23.36]
10. tr|A0A0E2WCS5|A0A0E2WCS5_MYCAV; K03151 thiI; tRNA uracil 4-sulfurtransferase [EC:2.8.1.4]
11. tr|A0A0E2W280|A0A0E2W280_MYCAV; K03154 thiS; sulfur carrier protein
12. tr|A0A0E2W629|A0A0E2W629_MYCAV; K03432 psmA, prcA; proteasome alpha subunit [EC:3.4.25.1]
13. tr|A0A0E2WKX6|A0A0E2WKX6_MYCAV; K05592 deaD, cshA; ATP-dependent RNA helicase DeaD [EC:3.6.4.13]
14. tr|A0A0E2W2B5|A0A0E2W2B5_MYCAV; K04043 dnaK, HSPA9; molecular chaperone DnaK
15. tr|A0A0E2W413|A0A0E2W413_MYCAV; K00937 ppk; polyphosphate kinase [EC:2.7.4.1]
16. tr|A0A0E2W2L0|A0A0E2W2L0_MYCAV; K22468 ppk2; polyphosphate kinase [EC:2.7.4.1]

15. Replication and Repair (DNA replication, Base excision repair, Nucleotide excision repair, Mismatch repair, Homologous recombination)

1. tr|A0A0E2WE41|A0A0E2WE41_MYCAV; K02342 dnaQ; DNA polymerase III subunit epsilon [EC:2.7.7.7]
2. tr|A0A0E2W8U0|A0A0E2W8U0_MYCAV; K02342 dnaQ; DNA polymerase III subunit epsilon [EC:2.7.7.7]
3. tr|A0A0E2W7E7|A0A0E2W7E7_MYCAV; K02337 dnaE; DNA polymerase III subunit alpha [EC:2.7.7.7]
4. tr|A0A0E2W7W6|A0A0E2W7W6_MYCAV; K02340 holA; DNA polymerase III subunit delta [EC:2.7.7.7]
5. tr|A0A0E2WX83|A0A0E2WX83_MYCAV; K02338 dnaN; DNA polymerase III subunit beta [EC:2.7.7.7]
6. tr|A0A0E2WRE7|A0A0E2WRE7_MYCAV; K02316 dnaG; DNA primase [EC:2.7.7.101]
7. tr|A0A0E2WCY0|A0A0E2WCY0_MYCAV; K03111 ssb; single-strand DNA-binding protein
8. tr|A0A0E2WC39|A0A0E2WC39_MYCAV; K10563 mutM, fpg; formamidopyrimidine-DNA glycosylase [EC:3.2.2.23 4.2.99.18]
9. tr|A0A0E2WSN0|A0A0E2WSN0_MYCAV; K01246 tag; DNA-3-methyladenine glycosylase I [EC:3.2.2.20]
10. tr|A0A0E2WFK3|A0A0E2WFK3_MYCAV; K21929 udg; uracil-DNA glycosylase [EC:3.2.2.27]
11. tr|A0A0E2W2E3|A0A0E2W2E3_MYCAV; K21929 udg; uracil-DNA glycosylase [EC:3.2.2.27]
12. tr|A0A0E2W799|A0A0E2W799_MYCAV; K01151 nfo; deoxyribonuclease IV [EC:3.1.21.2]
13. tr|A0A0E2WAW3|A0A0E2WAW3_MYCAV; K03703 uvrC; excinuclease ABC subunit C
14. tr|A0A0E2W8W1|A0A0E2W8W1_MYCAV; K03657 uvrD, pcrA; DNA helicase II / ATP-dependent DNA helicase crA [EC:3.6.4.12]
15. tr|A0A0E2WTT7|A0A0E2WTT7_MYCAV; K03657 uvrD, pcrA; DNA helicase II / ATP-dependent DNA helicase crA [EC:3.6.4.12]
16. tr|A0A0E2W8W1|A0A0E2W8W1_MYCAV; K03657 uvrD, pcrA; DNA helicase II / ATP-dependent DNA helicase crA [EC:3.6.4.12]
17. tr|A0A0E2WTT7|A0A0E2WTT7_MYCAV; K03657 uvrD, pcrA; DNA helicase II / ATP-dependent DNA helicase crA [EC:3.6.4.12]
18. tr|A0A0E2WCI1|A0A0E2WCI1_MYCAV; K03601 xseA; exodeoxyribonuclease VII large subunit [EC:3.1.11.6]
19. tr|A0A0E2WUC2|A0A0E2WUC2_MYCAV; K03602 xseB; exodeoxyribonuclease VII small subunit [EC:3.1.11.6]
20. tr|A0A0E2WCY0|A0A0E2WCY0_MYCAV; K03111 ssb; single-strand DNA-binding protein
21. tr|A0A0E2W7E7|A0A0E2W7E7_MYCAV; K02337 dnaE; DNA polymerase III subunit alpha [EC:2.7.7.7]
22. tr|A0A0E2WX83|A0A0E2WX83_MYCAV; K02338 dnaN; DNA polymerase III subunit beta [EC:2.7.7.7]
23. tr|A0A0E2W7W6|A0A0E2W7W6_MYCAV; K02340 holA; DNA polymerase III subunit delta [EC:2.7.7.7]
24. tr|A0A0E2WE41|A0A0E2WE41_MYCAV; K02342 dnaQ; DNA polymerase III subunit epsilon [EC:2.7.7.7]
25. tr|A0A0E2W8U0|A0A0E2W8U0_MYCAV; K02342 dnaQ; DNA polymerase III subunit epsilon [EC:2.7.7.7]
26. tr|A0A0E2WCY0|A0A0E2WCY0_MYCAV; K03111 ssb; single-strand DNA-binding protein
27. tr|A0A0E2WES3|A0A0E2WES3_MYCAV; K03629 recF; DNA replication and repair protein RecF
28. tr|A0A0E2W857|A0A0E2W857_MYCAV; K03584 recO; DNA repair protein RecO (recombination protein O)
29. tr|A0A0E2WIN0|A0A0E2WIN0_MYCAV; K06187 recR; recombination protein RecR
30. tr|A0A0E2W476|A0A0E2W476_MYCAV; K03550 ruvA; holliday junction DNA helicase RuvA [EC:3.6.4.12]
31. tr|A0A0E2WMC5|A0A0E2WMC5_MYCAV; K01159 ruvC; crossover junction endodeoxyribonuclease RuvC [EC:3.1.22.4]
32. tr|A0A0E2W0U5|A0A0E2W0U5_MYCAV; K03582 recB; exodeoxyribonuclease V beta subunit [EC:3.1.11.5]
33. tr|A0A0E2W385|A0A0E2W385_MYCAV; K03583 recC; exodeoxyribonuclease V gamma subunit [EC:3.1.11.5]
34. tr|A0A0E2W7D7|A0A0E2W7D7_MYCAV; K03581 recD; exodeoxyribonuclease V alpha subunit [EC:3.1.11.5]
35. tr|A0A0E2W7E7|A0A0E2W7E7_MYCAV; K02337 dnaE; DNA polymerase III subunit alpha [EC:2.7.7.7]
36. tr|A0A0E2WX83|A0A0E2WX83_MYCAV; K02338 dnaN; DNA polymerase III subunit beta [EC:2.7.7.7]
37. tr|A0A0E2W7W6|A0A0E2W7W6_MYCAV; K02340 holA; DNA polymerase III subunit delta [EC:2.7.7.7]
38. tr|A0A0E2WE41|A0A0E2WE41_MYCAV; K02342 dnaQ; DNA polymerase III subunit epsilon [EC:2.7.7.7]
39. tr|A0A0E2W8U0|A0A0E2W8U0_MYCAV; K02342 dnaQ; DNA polymerase III subunit epsilon [EC:2.7.7.7]
40. tr|A0A0E2W4V8|A0A0E2W4V8_MYCAV; K04066 priA; primosomal protein N' (replication factor Y) (superfamily I helicase) [EC:3.6.4.-]
41. tr|A0A0E2WGN5|A0A0E2WGN5_MYCAV; K10979 ku; DNA end-binding protein Ku

Environmental Information Processing:

16. Membrane Transport (transporters Phosphotransferase system, Bacterial secretion system)

1. tr|A0A0E2W7X3|A0A0E2W7X3_MYCAV; K23163 sbp; sulfate/thiosulfate transport system substrate-binding protein
2. tr|A0A0E2WR38|A0A0E2WR38_MYCAV; K02046 cysU; sulfate/thiosulfate transport system permease protein
3. tr|A0A0E2W9S5|A0A0E2W9S5_MYCAV; K02047 cysW; sulfate/thiosulfate transport system permease protein
4. tr|A0A0E2WPD9|A0A0E2WPD9_MYCAV; K15553 ssuA; sulfonate transport system substrate-binding protein
5. tr|A0A0E2WCE0|A0A0E2WCE0_MYCAV; K15554 ssuC; sulfonate transport system permease protein
6. tr|A0A0E2WX33|A0A0E2WX33_MYCAV; K15554 ssuC; sulfonate transport system permease protein
7. tr|A0A0E2W6P1|A0A0E2W6P1_MYCAV; K02018 modB; molybdate transport system permease protein
8. tr|A0A0E2WDY0|A0A0E2WDY0_MYCAV; K05846 opuBD; osmoprotectant transport system permease protein
9. tr|A0A0E2W814|A0A0E2W814_MYCAV; K10112 msmX, msmK, malK, sugC, ggtA, msiK; multiple sugar transport system ATP-binding protein
10. tr|A0A0E2WRF7|A0A0E2WRF7_MYCAV; K02066 mlaE, linK; phospholipid/cholesterol/gamma-HCH transport system permease protein
11. tr|A0A0E2WG74|A0A0E2WG74_MYCAV; K02066 mlaE, linK; phospholipid/cholesterol/gamma-HCH transport system permease protein
12. tr|A0A0E2WCA4|A0A0E2WCA4_MYCAV; K02066 mlaE, linK; phospholipid/cholesterol/gamma-HCH transport system permease protein
13. tr|A0A0E2W618|A0A0E2W618_MYCAV; K02066 mlaE, linK; phospholipid/cholesterol/gamma-HCH transport system permease protein
14. tr|A0A0E2WA79|A0A0E2WA79_MYCAV; K02066 mlaE, linK; phospholipid/cholesterol/gamma-HCH transport system permease protein
15. tr|A0A0E2WJ23|A0A0E2WJ23_MYCAV; K02066 mlaE, linK; phospholipid/cholesterol/gamma-HCH transport system permease protein
16. tr|A0A0E2WVK6|A0A0E2WVK6_MYCAV; K02066 mlaE, linK; phospholipid/cholesterol/gamma-HCH transport system permease protein
17. tr|A0A0E2W0P6|A0A0E2W0P6_MYCAV; K02066 mlaE, linK; phospholipid/cholesterol/gamma-HCH transport system permease protein
18. tr|A0A0E2W0V1|A0A0E2W0V1_MYCAV; K02066 mlaE, linK; phospholipid/cholesterol/gamma-HCH transport system permease protein
19. tr|A0A0E2W391|A0A0E2W391_MYCAV; K02066 mlaE, linK; phospholipid/cholesterol/gamma-HCH transport system permease protein
20. tr|A0A0E2WJ03|A0A0E2WJ03_MYCAV; K02066 mlaE, linK; phospholipid/cholesterol/gamma-HCH transport system permease protein
21. tr|A0A0E2W876|A0A0E2W876_MYCAV; K02066 mlaE, linK; phospholipid/cholesterol/gamma-HCH transport system permease protein
22. tr|A0A0E2WDH2|A0A0E2WDH2_MYCAV; K02066 mlaE, linK; phospholipid/cholesterol/gamma-HCH transport system permease protein
23. tr|A0A0E2WTR2|A0A0E2WTR2_MYCAV; K02040 pstS; phosphate transport system substrate-binding protein
24. tr|A0A0E2WSY3|A0A0E2WSY3_MYCAV; K02040 pstS; phosphate transport system substrate-binding protein
25. tr|A0A0E2WKR3|A0A0E2WKR3_MYCAV; K02040 pstS; phosphate transport system substrate-binding protein
26. tr|A0A0E2WC25|A0A0E2WC25_MYCAV; K02037 pstC; phosphate transport system permease protein
27. tr|A0A0E2WBI9|A0A0E2WBI9_MYCAV; K02038 pstA; phosphate transport system permease protein
28. tr|A0A0E2WAM4|A0A0E2WAM4_MYCAV; K02038 pstA; phosphate transport system permease protein
29. tr|A0A0E2WCP0|A0A0E2WCP0_MYCAV; K15580 oppA, mppA; oligopeptide transport system substrate-binding protein
30. tr|A0A0E2WWT5|A0A0E2WWT5_MYCAV; K15581 oppB; oligopeptide transport system permease protein
31. tr|A0A0E2WE99|A0A0E2WE99_MYCAV; K15582 oppC; oligopeptide transport system permease protein
32. tr|A0A0E2WA09|A0A0E2WA09_MYCAV; K16785 ecfT; energy-coupling factor transport system permease protein
33. tr|A0A0E2W8X5|A0A0E2W8X5_MYCAV; K09811 ftsX; cell division transport system permease protein
34. tr|A0A0E2WN40|A0A0E2WN40_MYCAV; K03072 secD; preprotein translocase subunit SecD
35. tr|A0A0E2W538|A0A0E2W538_MYCAV; K03074 secF; preprotein translocase subunit SecF
36. tr|A0A0E2WJR7|A0A0E2WJR7_MYCAV; K03073 secE; preprotein translocase subunit SecE
37. tr|A0A0E2W472|A0A0E2W472_MYCAV; K03210 yajC; preprotein translocase subunit YajC
38. tr|A0A0E2WJH1|A0A0E2WJH1_MYCAV; K03217 yidC, spoIIIJ, OXA1, ccfA; YidC/Oxa1 family membrane protein insertase
39. tr|A0A0E2WPX2|A0A0E2WPX2_MYCAV; K03070 secA; preprotein translocase subunit SecA [EC:7.4.2.8]
40. tr|A0A0E2WCC7|A0A0E2WCC7_MYCAV; K03116 tatA; sec-independent protein translocase protein TatA
41. tr|A0A0E2W633|A0A0E2W633_MYCAV; K03118 tatC; sec-independent protein translocase protein TatC

17. Signal Transduction (Two-component system, Phosphatidylinositol signaling system)

1. tr|A0A0E2W131|A0A0E2W131_MYCAV; K07768 senX3; two-component system, OmpR family, sensor histidine kinase SenX3 [EC:2.7.13.3]
2. tr|A0A0E2W3F7|A0A0E2W3F7_MYCAV; K07776 regX3; two-component system, OmpR family, response regulator RegX3
3. tr|A0A0E2WTR2|A0A0E2WTR2_MYCAV; K02040 pstS; phosphate transport system substrate-binding protein
4. tr|A0A0E2WSY3|A0A0E2WSY3_MYCAV; K02040 pstS; phosphate transport system substrate-binding protein
5. tr|A0A0E2WKR3|A0A0E2WKR3_MYCAV; K02040 pstS; phosphate transport system substrate-binding protein
6. tr|A0A0E2WAY2|A0A0E2WAY2_MYCAV; K07646 kdpD; two-component system, OmpR family, sensor histidine kinase KdpD [EC:2.7.13.3]
7. tr|A0A0E2WCD8|A0A0E2WCD8_MYCAV; K07667 kdpE; two-component system, OmpR family, KDP operon response regulator KdpE
8. tr|A0A0E2WU70|A0A0E2WU70_MYCAV; K01546 kdpA; potassium-transporting ATPase potassium-binding subunit
9. tr|A0A0E2W5A3|A0A0E2W5A3_MYCAV; K02259 COX15, ctaA; cytochrome c oxidase assembly protein subunit 15
10. tr|A0A0E2WC59|A0A0E2WC59_MYCAV; K07653 mprB; two-component system, OmpR family, sensor histidine kinase MprB [EC:2.7.13.3]
11. tr|A0A0E2WTV7|A0A0E2WTV7_MYCAV; K07669 mprA; two-component system, OmpR family, response regulator MprA
12. tr|A0A0E2W8H9|A0A0E2W8H9_MYCAV; K07654 mtrB; two-component system, OmpR family, sensor histidine kinase MtrB [EC:2.7.13.3]
13. tr|A0A0E2W2M0|A0A0E2W2M0_MYCAV; K07670 mtrA; two-component system, OmpR family, response regulator MtrA
14. tr|A0A0E2WD50|A0A0E2WD50_MYCAV; K02313 dnaA; chromosomal replication initiator protein
15. tr|A0A0E2WAK5|A0A0E2WAK5_MYCAV; K07655 prrB; two-component system, OmpR family, sensor histidine kinase PrrB [EC:2.7.13.3]
16. tr|A0A0E2WGL5|A0A0E2WGL5_MYCAV; K07671 prrA; two-component system, OmpR family, response regulator PrrA
17. tr|A0A0E2WAY6|A0A0E2WAY6_MYCAV; K07656 trcS; two-component system, OmpR family, sensor histidine kinase TrcS [EC:2.7.13.3]
18. tr|A0A0E2WH03|A0A0E2WH03_MYCAV; K07672 trcR; two-component system, OmpR family, response regulator TrcR
19. tr|A0A0E2WG23|A0A0E2WG23_MYCAV; K07684 narL; two-component system, NarL family, nitrate/nitrite response regulator NarL
20. tr|A0A0E2WSS4|A0A0E2WSS4_MYCAV; K00370 narG, narZ, nxrA; nitrate reductase / nitrite oxidoreductase, alpha subunit [EC:1.7.5.1 1.7.99.-]
21. tr|A0A0E2WB68|A0A0E2WB68_MYCAV; K00371 narH, narY, nxrB; nitrate reductase / nitrite oxidoreductase, beta subunit [EC:1.7.5.1 1.7.99.-]
22. tr|A0A0E2W9S2|A0A0E2W9S2_MYCAV; K00373 narJ, narW; nitrate reductase molybdenum cofactor assembly chaperone NarJ/NarW
23. tr|A0A0E2W2F6|A0A0E2W2F6_MYCAV; K07695 devR; two-component system, NarL family, response regulator DevR
24. tr|A0A0E2W3Z1|A0A0E2W3Z1_MYCAV; K00990 glnD; [protein-PII] uridylyltransferase [EC:2.7.7.59]
25. tr|A0A0E2WLY9|A0A0E2WLY9_MYCAV; K04751 glnB; nitrogen regulatory protein P-II 1
26. tr|A0A0E2W919|A0A0E2W919_MYCAV; K03412 cheB; two-component system, chemotaxis family, protein-glutamate methylesterase/glutaminase [EC:3.1.1.61 3.5.1.44]
27. tr|A0A0E2W552|A0A0E2W552_MYCAV; K00425 cydA; cytochrome bd ubiquinol oxidase subunit I [EC:7.1.1.7]
28. tr|A0A0E2WBJ5|A0A0E2WBJ5_MYCAV; K00426 cydB; cytochrome bd ubiquinol oxidase subunit II [EC:7.1.1.7]

Cellular Processes:

18. Transport and Catabolism (Peroxisome)

1. tr|A0A0E2WRS9|A0A0E2WRS9_MYCAV; K00803 AGPS, agpS; alkyldihydroxyacetonephosphate synthase [EC:2.5.1.26]
2. tr|A0A0E2WHK3|A0A0E2WHK3_MYCAV; K00031 IDH1, IDH2, icd; isocitrate dehydrogenase [EC:1.1.1.42]

19. Cell Growth and Death (Caulobacter)

1. tr|A0A0E2WD50|A0A0E2WD50_MYCAV; K02313 dnaA; chromosomal replication initiator protein
2. tr|A0A0E2W8Z7|A0A0E2W8Z7_MYCAV; K03531 ftsZ; cell division protein FtsZ
3. tr|A0A0E2WAI5|A0A0E2WAI5_MYCAV; K03589 ftsQ; cell division protein FtsQ
4. tr|A0A0E2WF60|A0A0E2WF60_MYCAV; K03588 ftsW, spoVE; cell division protein FtsW
5. tr|A0A0E2W8X9|A0A0E2W8X9_MYCAV; K02563 murG; UDP-N-acetylglucosamine--N-acetylmuramyl-(pentapeptide) pyrophosphoryl-undecaprenol N-acetylglucosamine transferase [EC:2.4.1.227]

20. Cellular community – prokaryotes (Quorum sensing, Biofilm formation of *Vibrio cholera, Pseudomonas aeruginosa, Escherichia coli*)

1. tr|A0A0E2WNU3|A0A0E2WNU3_MYCAV; K01657 trpE; anthranilate synthase component I [EC:4.1.3.27]D tr|A0A0E2WR93|A0A0E2WR93_MYCAV; K01626 E2.5.1.54, aroF, aroG, aroH; 3-deoxy-7-phosphoheptulonate synthase [EC:2.5.1.54]
2. tr|A0A0E2WAF0|A0A0E2WAF0_MYCAV; K01626 E2.5.1.54, aroF, aroG, aroH; 3-deoxy-7-phosphoheptulonate synthase [EC:2.5.1.54]
3. tr|A0A0E2WCD8|A0A0E2WCD8_MYCAV; K07667 kdpE; two-component system, OmpR family, KDP operon response regulator KdpE
4. tr|A0A0E2WAJ2|A0A0E2WAJ2_MYCAV; K11752 ribD; diaminohydroxyphosphoribosylaminopyrimidine deaminase / 5-amino-6-(5-phosphoribosylamino)uracil reductase [EC:3.5.4.26 1.1.1.193]
5. tr|A0A0E2WCP0|A0A0E2WCP0_MYCAV; K15580 oppA, mppA; oligopeptide transport system substrate-binding protein
6. tr|A0A0E2WWT5|A0A0E2WWT5_MYCAV; K15581 oppB; oligopeptide transport system permease protein
7. tr|A0A0E2WE99|A0A0E2WE99_MYCAV; K15582 oppC; oligopeptide transport system permease protein
8. tr|A0A0E2WAR7|A0A0E2WAR7_MYCAV; K02035 ABC.PE.S; peptide/nickel transport system substrate-binding protein
9. tr|A0A0E2WQA2|A0A0E2WQA2_MYCAV; K02035 ABC.PE.S; peptide/nickel transport system substrate-binding protein
10. tr|A0A0E2W6H8|A0A0E2W6H8_MYCAV; K02035 ABC.PE.S; peptide/nickel transport system substrate-binding protein
11. tr|A0A0E2WMZ6|A0A0E2WMZ6_MYCAV; K02033 ABC.PE.P; peptide/nickel transport system permease protein
12. tr|A0A0E2WDI6|A0A0E2WDI6_MYCAV; K02033 ABC.PE.P; peptide/nickel transport system permease protein
13. tr|A0A0E2W509|A0A0E2W509_MYCAV; K02034 ABC.PE.P1; peptide/nickel transport system permease protein
14. tr|A0A0E2W7D1|A0A0E2W7D1_MYCAV; K02034 ABC.PE.P1; peptide/nickel transport system permease protein
15. tr|A0A0E2WJH1|A0A0E2WJH1_MYCAV; K03217 yidC, spoIIIJ, OXA1, ccfA; YidC/Oxa1 family membrane protein insertase
16. tr|A0A0E2WJR7|A0A0E2WJR7_MYCAV; K03073 secE; preprotein translocase subunit SecE
17. tr|A0A0E2W472|A0A0E2W472_MYCAV; K03210 yajC; preprotein translocase subunit YajC
18. tr|A0A0E2WPX2|A0A0E2WPX2_MYCAV; K03070 secA; preprotein translocase subunit SecA [EC:7.4.2.8]
19. tr|A0A0E2W9Y9|A0A0E2W9Y9_MYCAV; K00640 cysE; serine O-acetyltransferase [EC:2.3.1.30]
20. tr|A0A0E2W449|A0A0E2W449_MYCAV; K03087 rpoS; RNA polymerase nonessential primary-like sigma factor
21. tr|A0A0E2WNU3|A0A0E2WNU3_MYCAV; K01657 trpE; anthranilate synthase component I [EC:4.1.3.27]
22. tr|A0A0E2W449|A0A0E2W449_MYCAV; K03087 rpoS; RNA polymerase nonessential primary-like sigma factor
23. tr|A0A0E2WD28|A0A0E2WD28_MYCAV; K04761 oxyR; LysR family transcriptional regulator, hydrogen peroxide-inducible genes activator

21. Cell motility (Bacterial chemotaxis)

1. tr|A0A0E2W919|A0A0E2W919_MYCAV; K03412 cheB; two-component system, chemotaxis family, protein-glutamate methylesterase/glutaminase [EC:3.1.1.61 3.5.1.44]

Organismal Systems

22. Immune system

1. tr|A0A0E2W738|A0A0E2W738_MYCAV; K03671 trxA; thioredoxin 1

23. Endocrine system

1. tr|A0A0E2WBJ9|A0A0E2WBJ9_MYCAV; K00873 PK, pyk; pyruvate kinase [EC:2.7.1.40]
2. tr|A0A0E2WMM5|A0A0E2WMM5_MYCAV; K00255 ACADL; long-chain-acyl-CoA dehydrogenase [EC:1.3.8.8]
3. tr|A0A0E2W6T2|A0A0E2W6T2_MYCAV; K00249 ACADM, acd; acyl-CoA dehydrogenase [EC:1.3.8.7]
4. tr|A0A0E2WPJ7|A0A0E2WPJ7_MYCAV; K00249 ACADM, acd; acyl-CoA dehydrogenase [EC:1.3.8.7]

24. Environmental adaptation

1. tr|A0A0E2W5A3|A0A0E2W5A3_MYCAV; K02259 COX15, ctaA; cytochrome c oxidase assembly protein subunit 15

Human Diseases

25. Cancers (Chemical and Viral carcinogenesis)

1. tr|A0A0E2WBJ9|A0A0E2WBJ9_MYCAV; K00873 PK, pyk; pyruvate kinase [EC:2.7.1.40]
2. tr|A0A0E2W3B4|A0A0E2W3B4_MYCAV; K00121 frmA, ADH5, adhC; S-(hydroxymethyl)glutathione dehydrogenase / alcohol dehydrogenase [EC:1.1.1.284 1.1.1.1]
3. tr|A0A0E2WBJ9|A0A0E2WBJ9_MYCAV; K00873 PK, pyk; pyruvate kinase [EC:2.7.1.40]

26. Cardiovascular diseases

1. tr|A0A0E2W738|A0A0E2W738_MYCAV; K03671 trxA; thioredoxin 1
27. Endocrine and metabolic diseases
1. tr|A0A0E2WBJ9|A0A0E2WBJ9_MYCAV; K00873 PK, pyk; pyruvate kinase [EC:2.7.1.40]
28. Infectious diseases (Bacterial and Viral)
1. tr|A0A0E2WID1|A0A0E2WID1_MYCAV; K14952 namH; UDP-MurNAc hydroxylase
2. tr|A0A0E2WTR2|A0A0E2WTR2_MYCAV; K02040 pstS; phosphate transport system substrate-binding protein
3. tr|A0A0E2WSY3|A0A0E2WSY3_MYCAV; K02040 pstS; phosphate transport system substrate-binding protein
4. tr|A0A0E2WKR3|A0A0E2WKR3_MYCAV; K02040 pstS; phosphate transport system substrate-binding protein
5. tr|A0A0E2W2B5|A0A0E2W2B5_MYCAV; K04043 dnaK, HSPA9; molecular chaperone DnaK
6. tr|A0A0E2WBJ9|A0A0E2WBJ9_MYCAV; K00873 PK, pyk; pyruvate kinase [EC:2.7.1.40]
29. Drug resistance: Antimicrobial (beta-Lactam resistance, Vancomycin resistance)
1. tr|A0A0E2WCP0|A0A0E2WCP0_MYCAV; K15580 oppA, mppA; oligopeptide transport system substrate-binding protein
2. tr|A0A0E2WWT5|A0A0E2WWT5_MYCAV; K15581 oppB; oligopeptide transport system permease protein
3. tr|A0A0E2WE99|A0A0E2WE99_MYCAV; K15582 oppC; oligopeptide transport system permease protein
4. tr|A0A0E2WF36|A0A0E2WF36_MYCAV; K03587 ftsI; cell division protein FtsI (penicillin-binding protein 3) [EC:3.4.16.4]
5. tr|A0A0E2W1R0|A0A0E2W1R0_MYCAV; K01775 alr; alanine racemase [EC:5.1.1.1]
6. tr|A0A0E2WS61|A0A0E2WS61_MYCAV; K01929 murF; UDP-N-acetylmuramoyl-tripeptide--D-alanyl-D-alanine ligase [EC:6.3.2.10]
7. tr|A0A0E2WAI2|A0A0E2WAI2_MYCAV; K01000 mraY; phospho-N-acetylmuramoyl-pentapeptide-transferase [EC:2.7.8.13]
8. tr|A0A0E2W8X9|A0A0E2W8X9_MYCAV; K02563 murG; UDP-N-acetylglucosamine--N-acetylmuramyl-(pentapeptide) pyrophosphoryl-undecaprenol N-acetylglucosamine transferase [EC:2.4.1.227]
30. Drug resistance: Antineoplastic
1. tr|A0A0E2W6Z5|A0A0E2W6Z5_MYCAV; K17686 copA, ctpA, ATP7; P-type Cu+ transporter [EC:7.2.2.8]
